# Supplementary material for: Combined Experimental and System-Level Analyses Reveal the Complex Regulatory Network of miR-124 during Human Neurogenesis
Source: Cell Syst. 2018 Oct 24;7(4):438–452.e8. doi: 10.1016/j.cels.2018.08.011 (PMC6205824; doi:10.1016/j.cels.2018.08.011)
Supplement: Document S1. Figures S1–S7 and Tables S2–S5 [file mmc1.pdf]

Cell Systems, Volume 7

Supplemental Information

Combined Experimental and System-Level Analyses Reveal the Complex Regulatory Network of miR-124 during Human Neurogenesis

Lisa K. Kutsche, Deisy M. Gysi, Joerg Fallmann, Kerstin Lenk, Rebecca Petri, Anka Swiersy, Simon D. Klapper, Karolina Pircs, Shahryar Khattak, Peter F. Stadler, Johan Jakobsson, Katja Nowick, and Volker Busskamp

Supplemental Information

Supplemental Tables S2 – S5 (Tables S1, S6-15 are provided as separate Excel files).

Supplemental Figures S1 – S7 including legends.

Table S2. Oligos for sgRNA cloning. Related to Figure 1.

sgRNA	Sequence (PAM)	Oligos
**miR-124-5'-1/2**	ATCAAGGTCCGCTGTGAACA (CGG)	CACCGATCAAGGTCCGCTGTGAACA, AAACTGTTCACAGCGGACCTTGATC
**miR-124-5'-3**	GTCTATACAATTAAGGCACG (CGG)	CACCGTCTATACAATTAAGGCACG, AAACCGTGCCTTAATTGTATAGAC
**miR-124-3'-1**	CGCGGTGAATGCCAAGAATG (GGG)	CACCGCGCGGTGAATGCCAAGAATG, AAACCATTCTTGGCATTCACCGCGC
**miR-124-3'-2/3**	CACGCGGTGAATGCCAAGAG (AGG)	CACCGCACGCGGTGAATGCCAAGAG, AAACCTCTTGGCATTCACCGCGTGC

Table S3. Genotyping primers for miR-124 expressing loci. Related to Figure 1.

Primer/Locus	Sequence
**miR-124-1_For**	TTATCTCATTGTCTGTGTGATTGGG
**miR-124-1_Rev**	CCCTGAGTCTGTTTGCATCTCTAAG
**miR-124-2_For**	AGGCTTATGTATGTTTTTAGGCGTG
**miR-124-2_Rev**	AAGAGATGGGTTCCTTTTACTACCC
**miR-124-3_For**	GAAAGGGGAGAAGTGTGGGCTCCTC
**miR-124-3_Rev**	GCCATTTCCATGAGAAAGGAGCGG

Table S4. Off-target primers to detect top predicted off-targets. Related to Figure S1 and STAR Methods.

Gene	Alternative sgRNA (PAM)	Primers used
**SNAPC4, NM_003086**	GACCAGGTCCGCAGTGAACA (GAG)	For: CTTGGTTCCTGTTTGTCTGTCCC<br>Rev: TGTCCAGCTCCACAATTAAAAACA

**THRA, NM_199334**	ATCTAGAACCACTGTGAACA (GAG)	For: GGATATTCGAGTAACCACCGTCA<br>Rev: AATTTCCGCCTCTGTTTCCAATG
**ASB12, NM_130388**	ATCACGGGCAGCTGTGAGCA (CGG)	For: AAACGTTTCATCAACAGCAGGAG<br>Rev: GAAGCTATGTTTGATGCCCAGAC
**C1orf87, NM_152377**	GTCTATAGCAATAAGGCACA (GGG)	For: CTTGGGTGGATAACCTTTTCATAGC<br>Rev: TCCTTTTCTGCCTTGTTTTTCACTT
**PARD6B, NM_032521**	GTCTTTACAAATACGGCAC G(GAG)	For: TATTGCGTCCTGACAACCATAGAAA<br>Rev: TCTCACTGTTATGATGAGGTTACGG
**PCYOX1L, NM_024028**	GGCCGTGGCTGCCAAGAATG (TGG)	For: CTAAAGACCCTGTTCCGTTCCTATT<br>Rev: TGATACATGAGGGGTCAGAAAGAAG
**GIPR, NM_000164**	ACAGGAGAATGCCAAGAATG (CGG)	For: ATTTGGTGGATTATACGGACCCC<br>Rev: TGGAAGGAGCTGAGGAAGATCTCAA
**ZAN, NM_173059**	CAGGGTGACAGCCAAGAATG (AGG)	For: CAGTGGTAGTGGTTAGGGAGCA<br>Rev: GTGGAAAGGTTCAGGGATTGGAAGG
**ZNF540, NM_001172226**	CCCTATGAATGTCAAGAATG (TGG)	For: TCAACTGATAGAAAACGTCCCTCTT<br>Rev: CCCTTATCACATTTCTTACACATATAGGG
**ZNF331, NM_001253800**	CCCTATGAATGTCAAGAATG (TGG)	For: GGAAGACCTTTAGCCGTGTGT<br>Rev: TCTGTGCACTTGTACGGCTT
**CLN3, NM_001042432**	CAGCAGGTGAAGGCCAAGAG (GAG)	For: CTCTGCGGTCTCACTCTATTCTC<br>Rev: GACAGAATGAATCCCTTTCCTCTGG
**SNX20, NM_153337**	TACGGGTTGAAAGCCAAGAG (TGG)	For: ATAAGCATTAGAGACCTGCAATCAC<br>Rev: GGGAGGGCATTTTCTTGATCTTAC
**CCDC62, NM_201435**	CTTGCGATGAATGCAAAGAG (AAG)	For: CACCCAAAAGTCGATATTAAGAGGG<br>Rev: TGACATTTTGGTGATTTTGTGTGGA

Table S5. Luciferase assay primers for 3‘ UTR amplification. Isoforms are discriminated according to Ensembl. Related to Figure 4.

Gene	Primers used
**CTDSP1-201**	For: TGAGGGTGATGGGGCCAG<br>Rev: TAGGGGTCTGGCTTTCTCTTTAGTT
**CD164-201**	For: ACAGACCCATTGAATTAATAAGGACTG<br>Rev: TTCAATAAACATGTAGATTTATTTTAAGTCAGTTTG
**CERS2-201**	For: ACCATTACTCCAGCTGCCTCC<br>Rev: TGCAAGGAAGGCATAAGAAAGACTC
**GLIS2-201**	For: GCCCATCCTGCGGACAGTTGTGGTG<br>Rev: CATCTTTGCAACTCACTTTTATTGT
**TP73-211**	For: AGATCCCCGAGCAGTACCGCATGAC<br>Rev: TCATTGGAACATACACTTTATTATG
**BAX-201**	For: TCAATCCCCGATTCATCTACCCTGC<br>Rev: TGTGTCCCGAAGGAGGTTTATTACC
**PPM1F-201**	For: GTGGTTTCCAGGCCCCTGCCCTCCC<br>Rev: CAGAGTTCAGAACTTGTGGTTTATT
**CDIP1-201**	For: CGGAGCTGGGACTCGGGACTCCCCC<br>Rev: TTTAAACAATTCATTCTCTAAAAGA
**ADGRL1-201**	For: GGGCACCTCATGGACCAGGGG<br>Rev: TCTTAATTTCTTTATTGTTTGACTTTTTGAC
**ARF1-214**	For: ACGCGACCCCCCTCCCTCT<br>Rev: GAAATAGTTAAGAGATTTATTCTAATAGCTATAA
**ARHGAP4-203**	For: GGTGCCGCTGCTGGAGATGC<br>Rev: GGTGCCGCTGCTGGAGATGC

**ASIC1**	For: GCCCCGCAGGCCGCT<br>Rev: TGGTTGAAACCCAGTTTATTAGACCAGAGGCATG
**ATF5-201**	For: AAGGGCAGGGGTGTGGCTTCTG<br>Rev: CCAGAGGAAATGTTTCGTTTTATTTTTGCTCAT
**CALM1-201**	For: AGACCTACTTTCAACTCCTTTTTCCCC<br>Rev: TGAAAGGCTGCAGAAATGTTTATTGAATACAGTG
**CAMK2B-209**	For: AGCTGCGCCCTGGTTTCG<br>Rev: TGTCCTGTTTTTCTTTTATTTGCAGTTTCCCGAG
**CRTAP-201**	For: CCCACAGCAACCAAAGAGACTTC<br>Rev: TAAGCAATGTAAAACATTTAATGTGATTTAAAAAG
**FAM102A-203**	For: GGAGCAGGTGTCCGGGCTG<br>Rev: AAAGAAACAAAAACCGAAACATTTAATTTTTTCTCCCC
**FAM104A-202**	For: TGTGCCGGCAGTTTCTTGCCTTC<br>Rev: TGAATGACAAAATGCACTAATGCTGCTTTTAATG
**FKBP1A-204**	For: ATCTGCCATGGAGGGATCTGGTG<br>Rev: TGAGAAAAGCCGGCATAAAGCACTTTTATTG
**GRM4-202**	For: AGTCCATGGAGCTGAGCAGCAG<br>Rev: ATGGTGACATGCTTTAATTATCCACCGAG
**KIAA2013-201**	For: GTGAACTAACAGTCCTGCTTT<br>Rev: TGAAAGTTTGAAAGATATTTATTTAAAAACAGAA
**LEMD2-201**	For: GCCCCGGGCGGGGAC<br>Rev: GGCAAGAAACATCCGTTTTAATAAATAGTTTATTC
**LFNG-201_UTR**	For: TGGCCATGGCTGAGACCCAA<br>Rev: TTGCTGCAAAGAGCACCTTTATTCACAG
**LFNG-203**	For: GGCACGGAGCAGCTGATGCC<br>Rev: TTTGAGGAAGGAAAAGCACCGCAGAGC
**MAFG-201**	For: GGACGCGCGTCTGC<br>Rev: CTCTTTAAAAGGTTTATTGATCATATACA
**MTMR10-201**	For: AATAGGGTGTTTTCTGAACATTTTGAG<br>Rev: AACAGTCGCTGTGGAATTTTATTAAG
**PDK2-203**	For: GGGCCGCCGTGCATCTG<br>Rev: TGGTCTTTGACCCATTCTCCAAGC
**PLEKHG5-212**	For: GCAGAGGGAGGCCCCCAAGAG<br>Rev: GCGGCCGCTGCTCCATAGCC
**POLR2E-213**	For: CCCCTAGAGGCGGACACACAG<br>Rev: TTCTTTCACAGGCACGTTTATTTTGCTGAAA
**PTPN18-201**	For: GTCTAACGCCAGTTCCTGCCTG<br>Rev: TGAATCGGAGAAATGGACAGTTTATTACTCACAG
**RASSF4-201**	For: CTGGCCAACACCTGCCTCTTCC<br>Rev: TTTGCTTTATCTTCGGATACGTTTATTACCTGAG
**RGS3-201**	For: GCAAACCCAAGGGCGGGAC<br>Rev: CCCTCTTAAAGAAATCTGTGTTTTTTCTTTC
**RPAIN-202**	For: AGCCAGCTTGGACTCACATC<br>Rev: GTTTTGTGTTCTCATTTATTATCATTTTTTTCTG
**RPLP1-201**	For: ACCTCTTTTATAACATGTTCAATAAAAAGC<br>Rev: GAACACACAATACCCACGGTTTATTATATAAATC
**SEMA5B-202**	For: TACCGCCGTCCTGGGGACT<br>Rev: GAGACTGGATATCATCTTTAATTAATAATGC
**SEMA5B-211**	For: TACCGCCGTCCTGGGGACT<br>Rev: GGAGACTGGATATCATCTTTAATTAATAATGC
**SERINC2-202**	For: GGCAGCCTCACAGCCTGC<br>Rev: GGTACACGCACTGGCTTGTTTATTAG
**SH3GLB2-203**	For: GCAGGTGCCCCCATCCC<br>Rev: TTTGCAGTTTTGGTAATTCTGTGGTCT
**SPRED2-201**	For: CTCAGTTTCCCTCCCTTCTCCC<br>Rev: TCTGATAGGATGTGTTTATATTTACACGGTAC
**SRM-201**	For: GCCCAGGCGCCACCACTG<br>Rev: TTTGCTATAAATACACGTGTTTGGTGAGTG

**STK11-201**	For: CCCGTGTCCAGGAGCCCC<br>Rev: CAGGTCCAAGCTTCCCAAGCTT
**SUMF1-201**	For: CAACCAAGGAAAGTCTTCCCCAG<br>Rev: CCTGATTTAGCAGATACGTTTATTCAACACA
**TACC3-201_UTR**	For: ACCTCCACGGAGCCGCTGT<br>Rev: TTTAAATTGAAAGGAAACTTTTATTGAGTC
**TMEM134-201**	For: TCGCGGCGCAGCGTGGACC<br>Rev: TGTGGCTATATCAGAAATTTTAATAAATAACAGTA
**TMEM63C-201**	For: CCGGGACCTGAGGCCTCCA<br>Rev: GAGGTTGGGAGGACTTGTTTATTCAAGG
**TNFRSF10B**	For: GTGTGATTCTCTTCAGGAAGTCAGAC<br>Rev: TTTTTATATAAGGTTTCATATTTAATTTGGTCATGG
**TRIT1-201**	For: GAGACATGTCCAGTGGCCTTTG<br>Rev: TCTTGTAAATTTTTTCTTTATTTAAACTTCAATAAAAATATAG
**TSPAN15-201**	For: GGCCCAGCCTGCCATGGCA<br>Rev: AAAAAAAAAAAAAAAAAACATGTTTTTATTGTTTGATTA
**UBE2G2-202**	For: GACCTGGCCTCGCACAGGC<br>Rev: TCACTGACACAGGAATCTGCCTCTTTATTGAC
**UBE2I-202**	For: GCAGCGACCTTGTGGCATCGT<br>Rev: GTCTGTGCAAATGCTTTAATGGTGG
**ZNF787-203**	For: GGGAGGGGCCCGGGGGGGGGG<br>Rev: GACATTTGGATAGTGCCGTTTATTG

A

iNGN **WT**, Karyotype: 46, XY [cp20] iNGN **ΔmiR-124**, Karyotype: 46, XY [cp20]

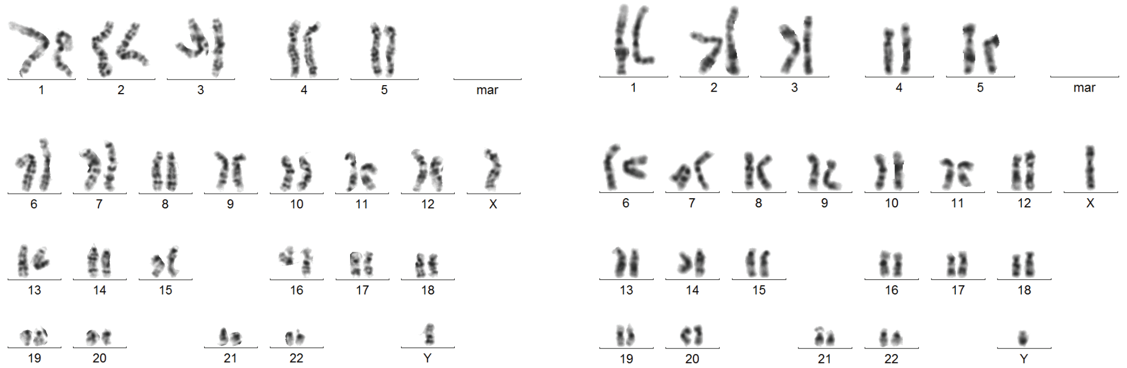

B

Gene	Score	Alternative sgRNA (PAM)	Sequence from Sanger sequencing
**SNAPC4, NM_003086**	0.6	GACCAGGTCCGCAGTGAACA (GAG)	G A C C A G G T C C G C A G T G A A C A
**THRA, NM_199334**	0.5	ATCTAGAACCACTGTGAACA (GAG)	T G T T C A C A G T G G T T C T A G A T
**ASB12, NM_130388**	0.3	ATCACGGGCAGCTGTGAGCA (CGG)	T G C T C A C A G C T G C C C G T G A T
**C1orf87, NM_152377**	0.2	GTCTATAGCAATAAGGCACA (GGG)	G T C T A T A G C A A T A A G G C A C A
**PARD6B, NM_032521**	0.2	GTCTTTACAAATACGGCAC G (GAG)	G T C T T T A C A A A T A C G G C A C G
**PCYOX1L, NM_024028**	0.9	GGCCGTGGCTGCCAAGAATG (TGG)	G G C C G T G G C T G C C A A G A A T G
**GIPR, NM_000164**	0.8	ACAGGAGAATGCCAAGAATG (CGG)	C A T T C T T G G C A T T C T C C T G T
**ZAN, NM_173059**	0.7	CAGGGTGACAGCCAAGAATG (AGG)	C A G G G T G A C A G C C A A G A A T G
**ZNF540, NM_001172226**	0.7	CCCTATGAATGTCAAGAATG (TGG)	C C C T A T G A A T G T C A A G A A T G
**ZNF331, NM_001253800**	0.7	CCCTATGAATGTCAAGAATG (TGG)	C C C T A T G A A T G T C A A G A A T G
**CLN3, NM_001042432**	0.7	CAGCAGGTGAAGGCCAAGAG (GAG)	C T C T T G G C C T T C A C C T G C T G
**SNX20, NM_153337**	0.5	TACGGGTTGAAAGCCAAGAG (TGG)	C T C T T G G C T T T C A A C C C G T A
**CCDC62, NM_201435**	0.3	CTTGCGATGAATGCAAAGAG (AAG)	C T T G C G A T G A A T G C A A A G A G

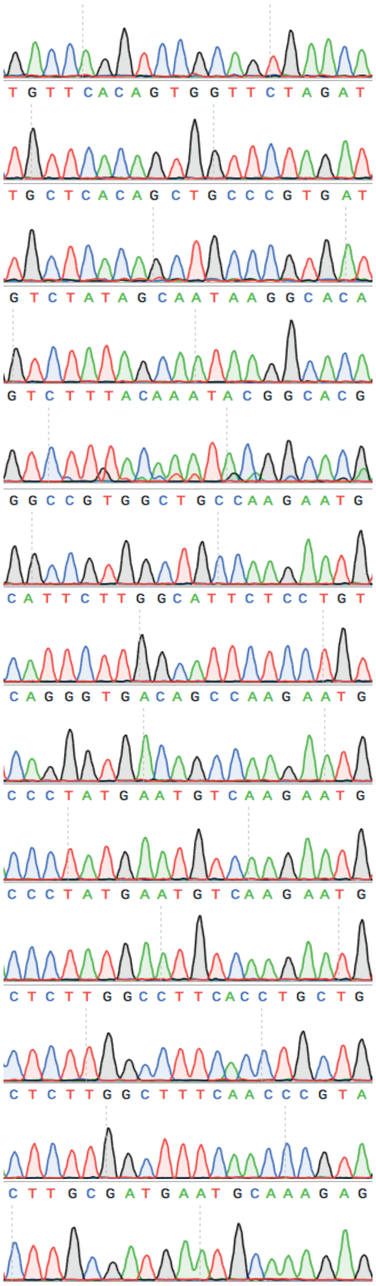

Figure S1. Karyotyping and off-target analysis of gene-edited human iPSCs. Related to Figure 1.

(A) The karyotypes were examined using G-banding of 20 metaphase chromosomal spreads.

(B) Top predicted off-targets and corresponding Sanger sequencing results indicate the lack of off-target gene editing. All on-target scores were > 80.

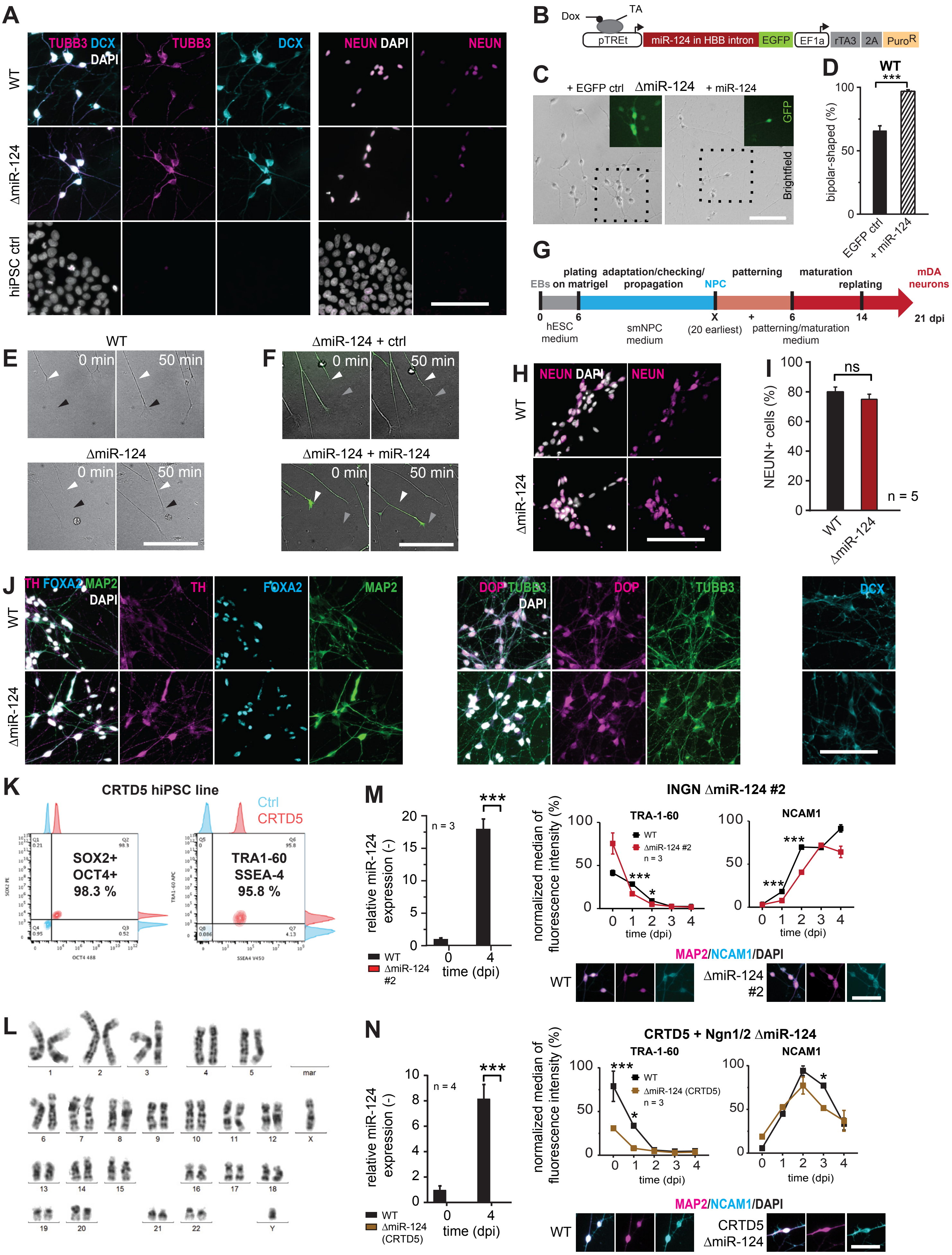
A
TUBB3 DCX DAPI
TUBB3
DCX
NEUN DAPI
NEUN
WT
ΔmiR-124
hiPSC ctrl
B
Dox
TA
pTREt
miR-124 in HBB intron
EGFP
EF1a
rTA3
2A
PuroR
C
+ EGFP ctrl
ΔmiR-124
+ miR-124
GFP
Brightfield
D
WT
***
bipolar-shaped (%)
EGFP ctrl
+ miR-124
G
plating on matrigel
adaptation/checking/ propagation
patterning
maturation
replating
EBs
NPC
mDA neurons
hESC medium
smNPC medium
(20 earliest)
patterning/maturation medium
21 dpi
E
WT
0 min
50 min
ΔmiR-124
F
ΔmiR-124 + ctrl
ΔmiR-124 + miR-124
H
NEUN DAPI
NEUN
I
ns
NEUN+ cells (%)
n = 5
J
TH FOXA2 MAP2 DAPI
TH
FOXA2
MAP2
DOP TUBB3 DAPI
DOP
TUBB3
DCX
K
CRTD5 hiPSC line
Ctrl
CRTD5
SOX2+ OCT4+ 98.3 %
TRA1-60 SSEA-4 95.8 %
M
n = 3
***
relative miR-124 expression (-)
time (dpi)
WT
ΔmiR-124 #2
INGN ΔmiR-124 #2
TRA-1-60
NCAM1
normalized median of fluorescence intensity (%)
n = 3
MAP2/NCAM1/DAPI
L
mar
N
n = 4
ΔmiR-124 (CRTD5)
CRTD5 + Ngn1/2 ΔmiR-124
CRTD5 ΔmiR-124

Figure S2. Extended phenotypic analyses of WT and ΔmiR-124 neurons. Related to Figures 1 and 2.

(A) Immunofluorescence staining for the indicated neuronal markers at 4 dpi with hiPSC as control (0 dpi).

(B) Scheme of the miR-124 overexpression PiggyBac vector. miR-124 was expressed from a human β-globin intron that was upstream of an EGFP reporter sequence and driven by a TetOn promoter. Dox – doxycycline, TA – transcriptional activator. The EGFP control vector (EGFP ctrl) lacked the miR-124 sequence.

(C) Representative images of miR-124-overexpressing ΔmiR-124 cells at 4 dpi. Corresponding quantification is shown in Figure 2F. Of note, EGFP ctrl expression was very high.

(D) Bipolar shape quantification of EGFP ctrl and miR-124-overexpressing WT iNGN cells at 4 dpi. The EGFP ctrl expression led to decreased bipolar morphology that was rescued by miR-124 overexpression.

(E) Representative examples for neurite outgrowth capability at 5 dpi, as quantified in Figure 2E.

(F) Representative examples of neurite outgrowth capability of miR-124-overexpressing ΔmiR-124 cells at 5 dpi, as quantified in Figure 2G.

(G) Scheme of the small-molecule-based protocol. EB – embryoid bodies, NPC – neural precursor cells, mDA – midbrain dopaminergic neurons.

(H) Representative images showing NEUN expression in WT and ΔmiR-124 cells, induced by the small-molecule-based protocol shown in (G).

(I) Quantification of NEUN expression of WT and ΔmiR-124 cells, induced by the small-molecule-based protocol as percentage of NEUN-positive nuclei. Statistical analysis, unpaired Student's t-test. Data are represented as mean ± SEM.

(J) Alternative differentiation using small molecules yields dopaminergic neurons for both WT and ΔmiR-124. TH, dopamine, and FOXA2 as dopaminergic markers are expressed, as well as MAP2, TUBB3, and DCX as pan-neuronal markers. Scale bar, 100μm.

(K) Pluripotency flow cytometry analysis of CRTD5 hiPSCs with Alexa Flour 488 anti-Oct3/4, PE anti-SOX2, V450-SSEA-4 and Alexa Flour 647 anti TRA-1-60 antibody markers.

(L) The karyotype of CRTD5 human iPSCs was examined using G-banding of 20 metaphase chromosomal spreads (one example shown).

(M, N) Analysis of a second ΔmiR-124 iNGN clone (M) and a ΔmiR-124 iNGN within the CRTD5 hiPSC background (N). Left, qRT-PCR analysis indicated the lack of miR-124 expression in ΔmiR-124 cells, tested in iPSCs (0 dpi) and neurons (4 dpi). Right, loss of pluripotency (TRA-1-60) and neural lineage commitment (NCAM1) from 0 – 4 dpi measured by flow cytometry using the surface markers indicated. Bottom, representative immunostainings of indicated neuronal markers at 4 dpi reveal miR-124-independent neurogenesis. Scale bar, 50 μm.

Statistical analysis was performed using unpaired Student's t-tests with *$p \leq 0.05$, ***$p \leq 0.001$.

See also Figure S1.

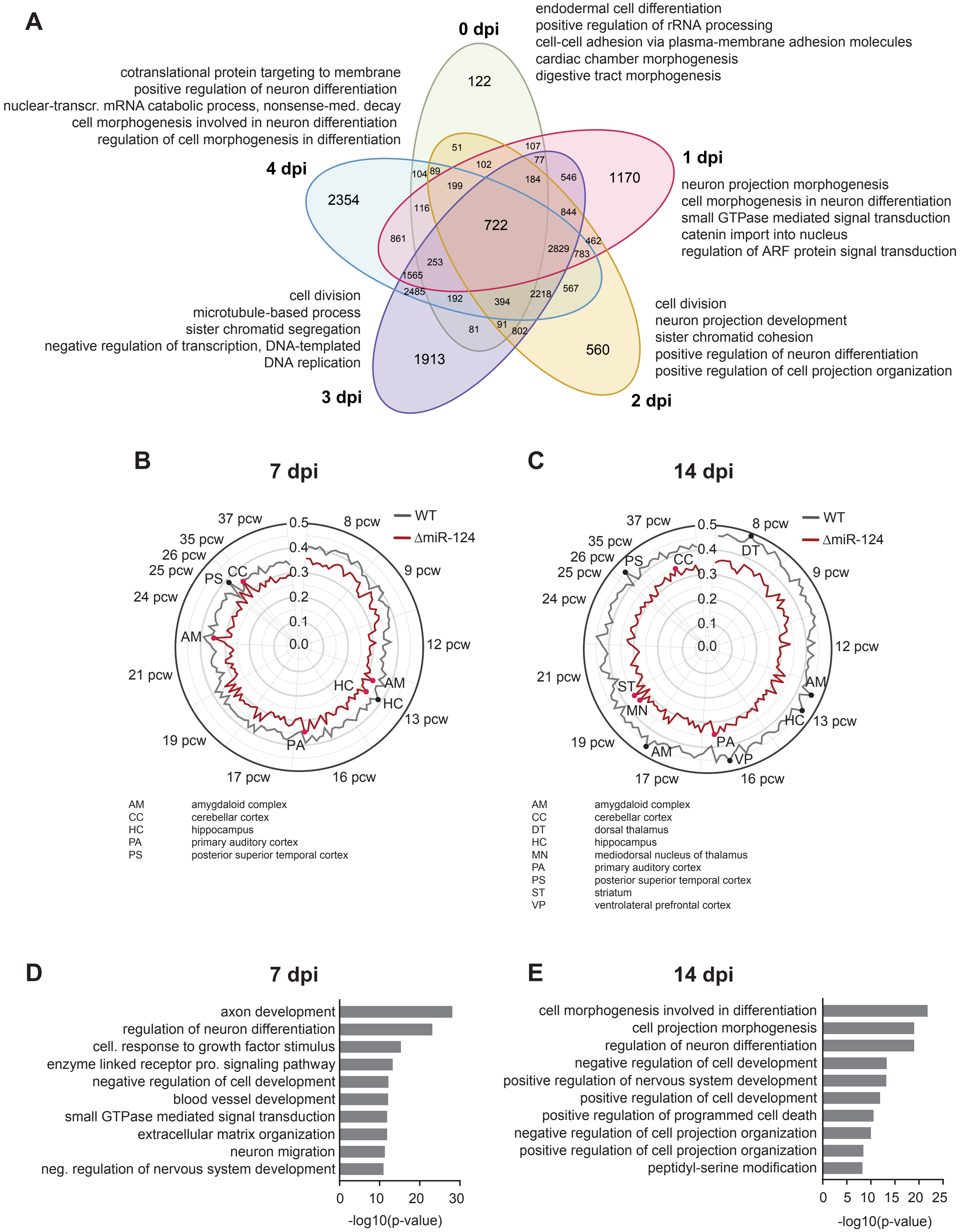
A
0 dpi
endodermal cell differentiation
positive regulation of rRNA processing
cell-cell adhesion via plasma-membrane adhesion molecules
cardiac chamber morphogenesis
digestive tract morphogenesis
cotranslational protein targeting to membrane
positive regulation of neuron differentiation
nuclear-transcr. mRNA catabolic process, nonsense-med. decay
cell morphogenesis involved in neuron differentiation
regulation of cell morphogenesis in differentiation
4 dpi
1 dpi
neuron projection morphogenesis
cell morphogenesis in neuron differentiation
small GTPase mediated signal transduction
catenin import into nucleus
regulation of ARF protein signal transduction
122
51
107
77
102
104
89
184
546
1170
2354
199
116
722
844
861
462
2829
783
253
1565
567
2485
192
394
2218
81
91
802
1913
560
cell division
microtubule-based process
sister chromatid segregation
negative regulation of transcription, DNA-templated
DNA replication
3 dpi
cell division
neuron projection development
sister chromatid cohesion
positive regulation of neuron differentiation
positive regulation of cell projection organization
2 dpi
B
7 dpi
WT
ΔmiR-124
37 pcw
8 pcw
35 pcw
26 pcw
25 pcw
9 pcw
24 pcw
12 pcw
21 pcw
13 pcw
19 pcw
17 pcw
16 pcw
0.5
0.4
0.3
0.2
0.1
0.0
PS
CC
AM
HC
AM
HC
PA
AM amygdaloid complex
CC cerebellar cortex
HC hippocampus
PA primary auditory cortex
PS posterior superior temporal cortex
C
14 dpi
WT
ΔmiR-124
37 pcw
8 pcw
35 pcw
26 pcw
25 pcw
9 pcw
24 pcw
12 pcw
21 pcw
13 pcw
19 pcw
17 pcw
16 pcw
DT
PS
CC
AM
ST
MN
HC
PA
VP
AM
AM amygdaloid complex
CC cerebellar cortex
DT dorsal thalamus
HC hippocampus
MN mediodorsal nucleus of thalamus
PA primary auditory cortex
PS posterior superior temporal cortex
ST striatum
VP ventrolateral prefrontal cortex
D
7 dpi
axon development
regulation of neuron differentiation
cell. response to growth factor stimulus
enzyme linked receptor pro. signaling pathway
negative regulation of cell development
blood vessel development
small GTPase mediated signal transduction
extracellular matrix organization
neuron migration
neg. regulation of nervous system development
0 10 20 30
-log10(p-value)
E
14 dpi
cell morphogenesis involved in differentiation
cell projection morphogenesis
regulation of neuron differentiation
negative regulation of cell development
positive regulation of nervous system development
positive regulation of cell development
positive regulation of programmed cell death
negative regulation of cell projection organization
positive regulation of cell projection organization
peptidyl-serine modification
0 5 10 15 20 25
-log10(p-value)

Figure S3. Extended transcriptome analysis. Related to Figure 3.

(A) Venn diagram for differential gene expression over the time course of differentiation (0 – 4 dpi) comparing WT and ΔmiR-124 with associated GO terms.

(B, C) Correlation analysis of WT and ΔmiR-124 neurons at 7 dpi (B) and 14 dpi (C) to the developing human brain. Pearson correlation of 1000 most differentially expressed genes compared to the transcriptomic Allen Brain Span Atlas dataset for the developmental stages from 8 to 37 postconceptional weeks (pcw).

(D, E) GO term enrichment analysis of differentially expressed genes between WT and ΔmiR-124 samples at 7 dpi (D) and 14 dpi (E) indicating their involvement in neuronal development, maturation and apoptosis.

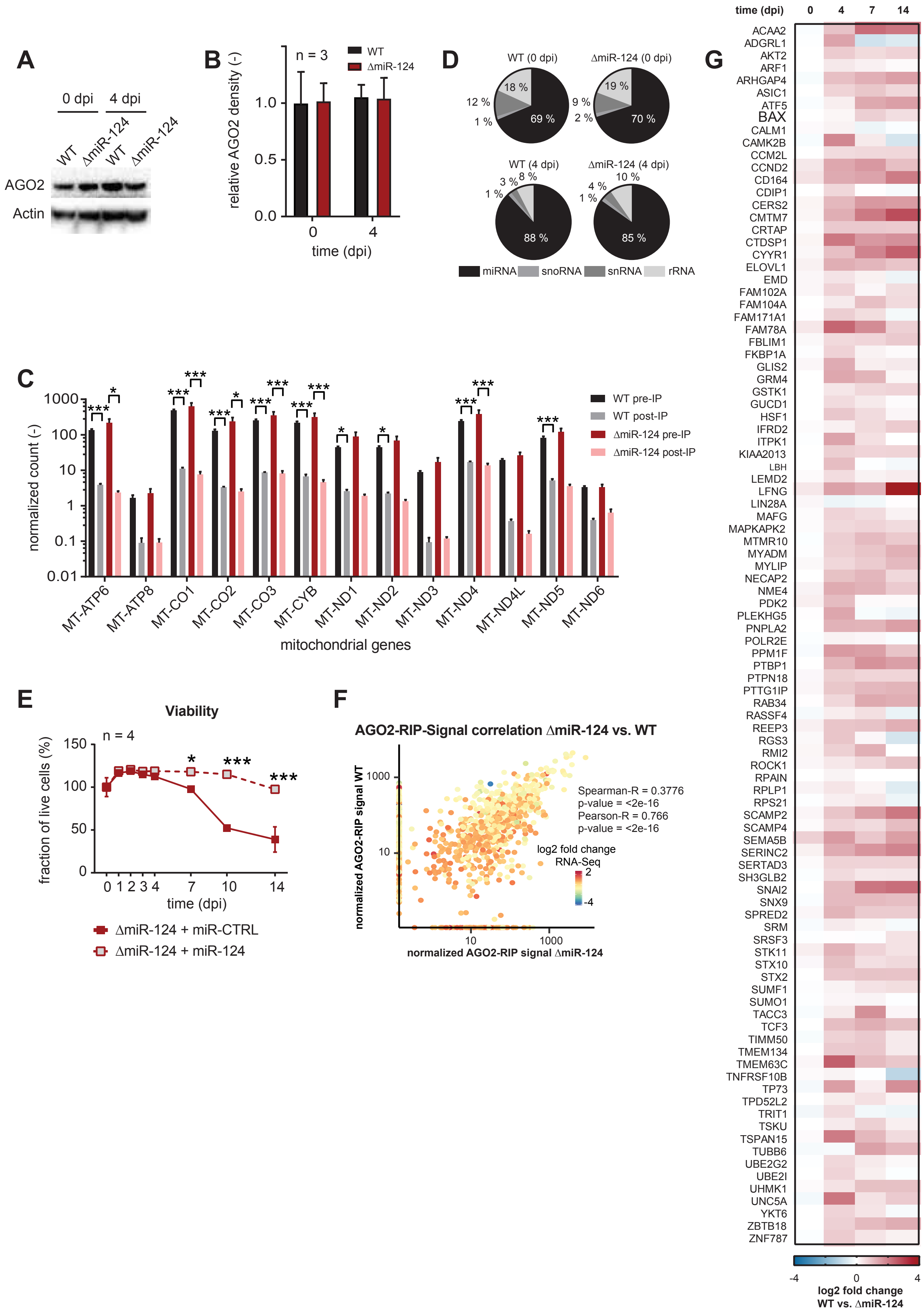

A
0 dpi
4 dpi
WT
ΔmiR-124
WT
ΔmiR-124
AGO2
Actin
B
n = 3
WT
ΔmiR-124
relative AGO2 density (-)
time (dpi)
D
WT (0 dpi)
ΔmiR-124 (0 dpi)
WT (4 dpi)
ΔmiR-124 (4 dpi)
miRNA
snoRNA
snRNA
rRNA
G
time (dpi)
0 4 7 14
ACAA2
ADGRL1
AKT2
ARF1
ARHGAP4
ASIC1
ATF5
BAX
CALM1
CAMK2B
CCM2L
CCND2
CD164
CDIP1
CERS2
CMTM7
CRTAP
CTDSP1
CYYR1
ELOVL1
EMD
FAM102A
FAM104A
FAM171A1
FAM78A
FBLIM1
FKBP1A
GLIS2
GRM4
GSTK1
GUCD1
HSF1
IFRD2
ITPK1
KIAA2013
LBH
LEMD2
LFNG
LIN28A
MAFG
MAPKAPK2
MTMR10
MYADM
MYLIP
NECAP2
NME4
PDK2
PLEKHG5
PNPLA2
POLR2E
PPM1F
PTBP1
PTPN18
PTTG1IP
RAB34
RASSF4
REEP3
RGS3
RMI2
ROCK1
RPAIN
RPLP1
RPS21
SCAMP2
SCAMP4
SEMA5B
SERINC2
SERTAD3
SH3GLB2
SNAI2
SNX9
SPRED2
SRM
SRSF3
STK11
STX10
STX2
SUMF1
SUMO1
TACC3
TCF3
TIMM50
TMEM134
TMEM63C
TNFRSF10B
TP73
TPD52L2
TRIT1
TSKU
TSPAN15
TUBB6
UBE2G2
UBE2I
UHMK1
UNC5A
YKT6
ZBTB18
ZNF787
log2 fold change
WT vs. ΔmiR-124
C
normalized count (-)
WT pre-IP
WT post-IP
ΔmiR-124 pre-IP
ΔmiR-124 post-IP
MT-ATP6
MT-ATP8
MT-CO1
MT-CO2
MT-CO3
MT-CYB
MT-ND1
MT-ND2
MT-ND3
MT-ND4
MT-ND4L
MT-ND5
MT-ND6
mitochondrial genes
E
Viability
n = 4
fraction of live cells (%)
time (dpi)
ΔmiR-124 + miR-CTRL
ΔmiR-124 + miR-124
F
AGO2-RIP-Signal correlation ΔmiR-124 vs. WT
normalized AGO2-RIP signal WT
normalized AGO2-RIP signal ΔmiR-124
Spearman-R = 0.3776
p-value = <2e-16
Pearson-R = 0.766
p-value = <2e-16
log2 fold change
RNA-Seq

Figure S4. Target validation and AGO2 precipitation quality control. Related to Figure 4.

(A) Western blot for AGO2 from WT and ΔmiR-124 samples at 0 and 4 dpi.

(B) Quantification of AGO2 western blots (C) using densiometry. n = 3 biological samples.

(C) Normalized count of mitochondrial genes pre- and post-AGO2-IP as indication for IP quality.

(D) Quantification of miRNA, snoRNA, and snRNA species from whole-cell samples. The fraction of miRNAs is significantly increasing from 0 dpi to 4 dpi. Comparisons between WT and ΔmiR-124 are non-significant.

(E) Assessment of cell viability from 0 – 14 dpi for ΔmiR-124 and rescue with miR-124 substitution. The percentage of live cells was measured using the ApoToxGlo assay. The ratio between fluorescence signal for live cells (AFC) and total cell fluorescence (AFC + R110) was examined and normalized to 100 % for 0 dpi.

(F) Correlation of AGO2-RIP signal for ΔmiR-124 and WT indicating differential expression in whole-cell samples as color code (log2 fold change).

(G) Heatmap showing the log2 fold changes of filtered miR-124 targets at 0, 4, 7 and 14 dpi (See also Figure 4C).

Statistical analysis with unpaired Student's t-tests with Holm-Sidak correction for multiple comparisons or 2-way ANOVA with Tukey's multiple comparison test with $^{*}p \leq 0.05$, $^{**}p \leq 0.01$, $^{***}p \leq 0.001$. Data are represented as mean ± SEM

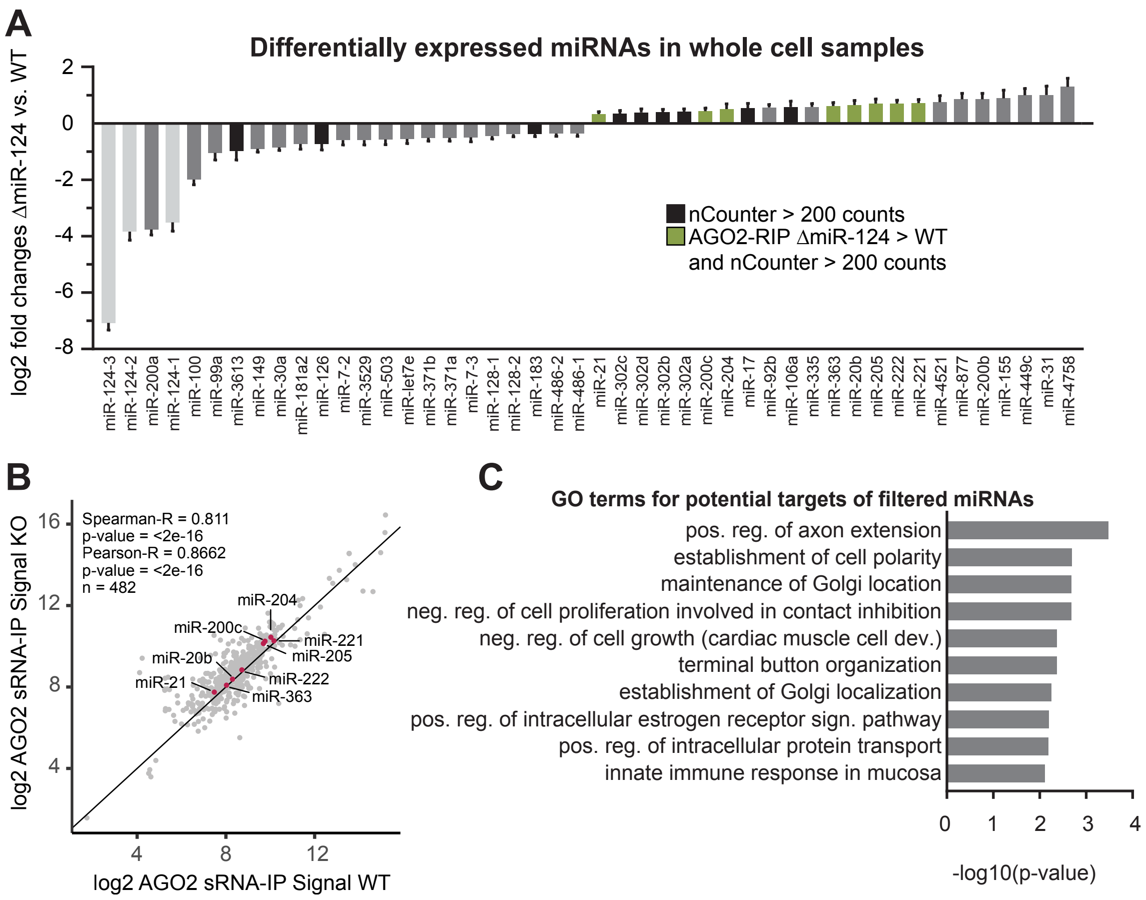

A
Differentially expressed miRNAs in whole cell samples
log2 fold changes ΔmiR-124 vs. WT
nCounter > 200 counts
AGO2-RIP ΔmiR-124 > WT
and nCounter > 200 counts
miR-124-3
miR-124-2
miR-200a
miR-124-1
miR-100
miR-99a
miR-3613
miR-149
miR-30a
miR-181a2
miR-126
miR-7-2
miR-3529
miR-503
miR-let7e
miR-371b
miR-371a
miR-7-3
miR-128-1
miR-128-2
miR-183
miR-486-2
miR-486-1
miR-21
miR-302c
miR-302d
miR-302b
miR-302a
miR-200c
miR-204
miR-17
miR-92b
miR-106a
miR-335
miR-363
miR-20b
miR-205
miR-222
miR-221
miR-4521
miR-877
miR-200b
miR-155
miR-449c
miR-31
miR-4758
B
Spearman-R = 0.811
p-value = <2e-16
Pearson-R = 0.8662
p-value = <2e-16
n = 482
log2 AGO2 sRNA-IP Signal KO
log2 AGO2 sRNA-IP Signal WT
miR-204
miR-200c
miR-221
miR-205
miR-20b
miR-21
miR-222
miR-363
C
GO terms for potential targets of filtered miRNAs
pos. reg. of axon extension
establishment of cell polarity
maintenance of Golgi location
neg. reg. of cell proliferation involved in contact inhibition
neg. reg. of cell growth (cardiac muscle cell dev.)
terminal button organization
establishment of Golgi localization
pos. reg. of intracellular estrogen receptor sign. pathway
pos. reg. of intracellular protein transport
innate immune response in mucosa
-log10(p-value)

Figure S5. Differentially expressed miRNAs. Related to Figure 5.

(A) Differentially expressed miRNAs from the whole-cell samples as revealed by small RNA-Sequencing at 4 dpi. Data are presented as mean ± lfcSE. Color code indicates miRNAs with more than 200 nCounter counts.

(B) Correlation of AGO2-RIP for ΔmiR-124 and WT at 4 dpi. The eight miRNAs derived from the analysis shown in Figure 5D are marked in red.

(C) GO term analysis for targets of the eight filtered miRNAs (Figure 5D) indicating an involvement in neuronal biological processes.

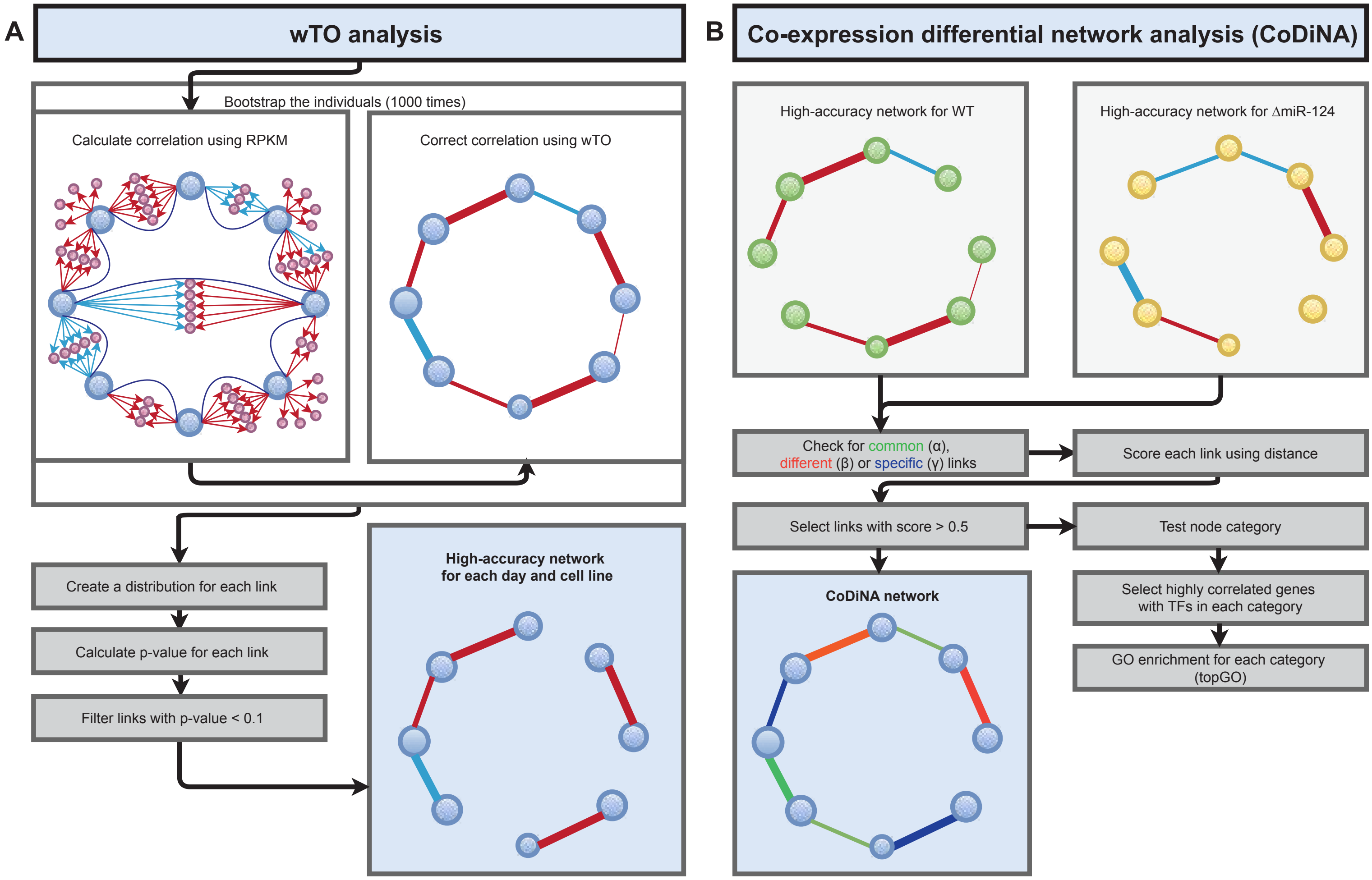
A
wTO analysis
Bootstrap the individuals (1000 times)
Calculate correlation using RPKM
Correct correlation using wTO
Create a distribution for each link
Calculate p-value for each link
Filter links with p-value < 0.1
High-accuracy network
for each day and cell line
B
Co-expression differential network analysis (CoDiNA)
High-accuracy network for WT
High-accuracy network for ΔmiR-124
Check for common (α),
different (β) or specific (γ) links
Score each link using distance
Select links with score > 0.5
Test node category
CoDiNA network
Select highly correlated genes
with TFs in each category
GO enrichment for each category
(topGO)

Figure S6. Illustration of computational network analyses. Related to Figures 6 and 7.

The workflows for the wTO (A) and co-expression differential network (CoDiNA) (B) analyses are shown.

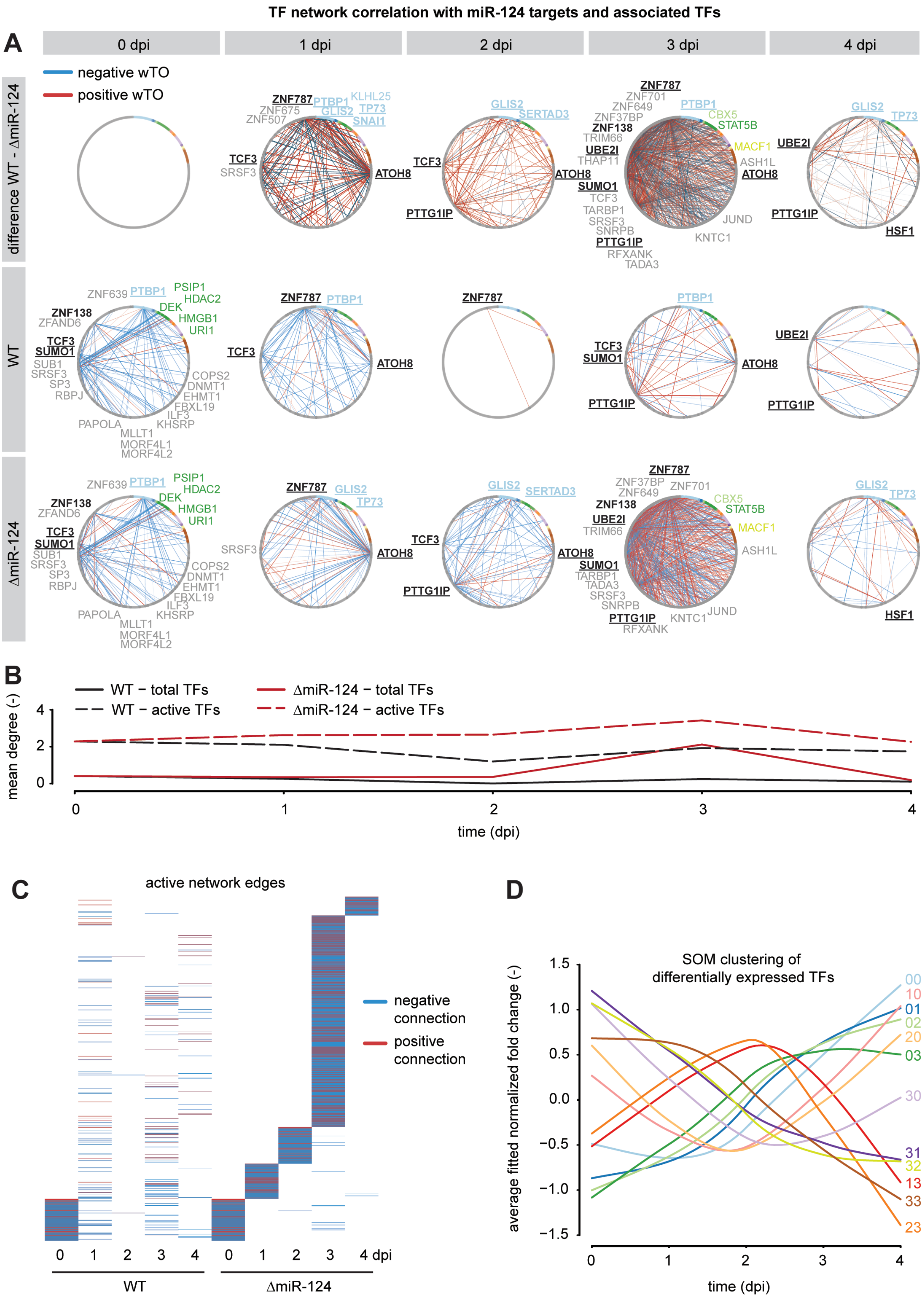

TF network correlation with miR-124 targets and associated TFs
A
0 dpi
1 dpi
2 dpi
3 dpi
4 dpi
negative wTO
positive wTO
difference WT - ΔmiR-124
WT
ΔmiR-124
B
WT − total TFs
WT − active TFs
ΔmiR-124 − total TFs
ΔmiR-124 − active TFs
mean degree (-)
time (dpi)
C
active network edges
negative connection
positive connection
WT
ΔmiR-124
D
SOM clustering of differentially expressed TFs
average fitted normalized fold change (-)
time (dpi)

Figure S7. Timecourse wTO analysis, edge activation and SOM classification of target-TF-network. Related to Figure 6.

(A) Expression correlation (as weighted topological overlap, wTO) between TFs that were differentially expressed on at least one day between 1 dpi and 4 dpi, but not on 0 dpi. Differences in interaction ($|wTO^{WT} - wTO^{\Delta miR\text{-}124}| > 0.2$) are shown in the top panel. Every panel shows the development of the network during differentiation for the difference (top), WT (middle), and ΔmiR-124 (bottom). The opacity of the line indicates the wTO value. Colored gene names represent a specific SOM cluster as shown in Figure S7D. Underlined TFs are miR-124 targets (Figure 4C).

(B) Degree of nodes for each network for active genes and full set of genes over time, as a measure for network strength.

(C) Edge activation of the network depicted gene relationships reappearing on different days.

(D) Loess regression from Self Organizing Maps calculated on the basis of normalized fold changes of permanently (1 dpi – 4 dpi) differentially expressed TFs. Color code represents the SOM categories.
