# Supplementary material for: Combined Experimental and System-Level Analyses Reveal the Complex Regulatory Network of miR-124 during Human Neurogenesis
Source: Cell Syst. 2018 Oct 24;7(4):438–452.e8. doi: 10.1016/j.cels.2018.08.011 (PMC6205824; doi:10.1016/j.cels.2018.08.011)
Supplement: Document S2. Article plus Supplemental Information [file mmc13.pdf]

Cell Systems

Article

Combined Experimental and System-Level Analyses Reveal the Complex Regulatory Network of miR-124 during Human Neurogenesis

Graphical Abstract

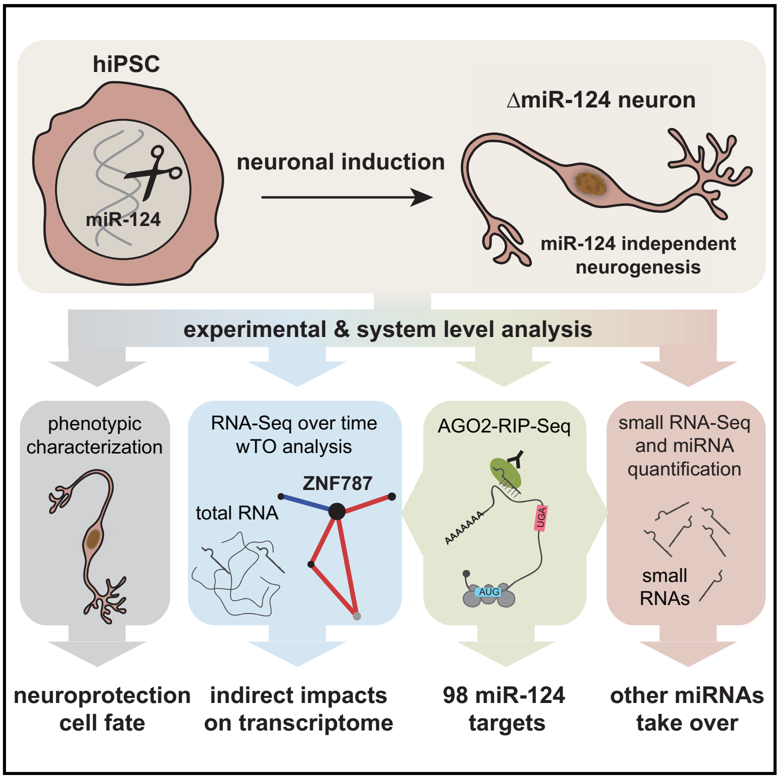

Highlights

- miR-124 is not essential for neurogenesis from human iPSCs
- miR-124 regulation mediates neuroprotection and refines neuronal cell fates
- miRNA knockout characterization by experimental and advanced computational analyses
- Identification of 98 targets including the neuronal feature repressor ZNF787

Authors

Lisa K. Kutsche, Deisy M. Gysi, Joerg Fallmann, ..., Johan Jakobsson, Katja Nowick, Volker Busskamp

Correspondence

volker.busskamp@tu-dresden.de

In Brief

Kutsche et al. studied the impact of miR-124 deletion in human stem cells during neurogenesis. Sophisticated experimental and systems biology analyses revealed multiple concurrent regulatory modes of miRNA regulation.

Kutsche et al., 2018, Cell Systems 7, 438–452
October 24, 2018 © 2018 The Author(s). Published by Elsevier Inc.
https://doi.org/10.1016/j.cels.2018.08.011

OPEN ACCESS
CellPress

Cell Systems
Article

Combined Experimental and System-Level Analyses Reveal the Complex Regulatory Network of miR-124 during Human Neurogenesis

Lisa K. Kutsche,[1,8] Deisy M. Gysi,[2,3,4,8] Joerg Fallmann,[2] Kerstin Lenk,[1] Rebecca Petri,[5] Anka Swiersy,[1] Simon D. Klapper,[1] Karolina Pircs,[5] Shahryar Khattak,[1] Peter F. Stadler,[2,6,7] Johan Jakobsson,[5] Katja Nowick,[4] and Volker Busskamp[1,9,*]
[1]Technische Universität Dresden, DFG Research Center for Regenerative Therapies, Dresden 01307, Germany
[2]Department of Computer Science, Bioinformatics Group, Interdisciplinary Center for Bioinformatics, University of Leipzig, Leipzig 04107, Germany
[3]Faculty of Mathematics and Computer Science, Swarm Intelligence and Complex Systems Group, University of Leipzig, Leipzig 04109, Germany
[4]Faculty for Biology, Chemistry and Pharmacy, Freie Universität Berlin, Institute for Biology, Berlin 14195, Germany
[5]Department of Experimental Medical Science, Laboratory of Molecular Neurogenetics, Wallenberg Neuroscience Center and Lund Stem Cell Center, Lunds Universitet, Lund 22184, Sweden
[6]Max Planck Institute for Mathematics in the Sciences, Leipzig 04103, Germany
[7]Santa Fe Institute, 1399 Hyde Park Road, Santa Fe, NM 87501, USA
[8]These authors contributed equally
[9]Lead Contact
*Correspondence: volker.busskamp@tu-dresden.de
https://doi.org/10.1016/j.cels.2018.08.011

SUMMARY

Non-coding RNAs regulate many biological processes including neurogenesis. The brain-enriched miR-124 has been assigned as a key player of neuronal differentiation via its complex but little understood regulation of thousands of annotated targets. To systematically chart its regulatory functions, we used CRISPR/Cas9 gene editing to disrupt all six miR-124 alleles in human induced pluripotent stem cells. Upon neuronal induction, miR-124-deleted cells underwent neurogenesis and became functional neurons, albeit with altered morphology and neurotransmitter specification. Using RNA-induced-silencing-complex precipitation, we identified 98 high-confidence miR-124 targets, of which some directly led to decreased viability. By performing advanced transcription-factor-network analysis, we identified indirect miR-124 effects on apoptosis, neuronal subtype differentiation, and the regulation of previously uncharacterized zinc finger transcription factors. Our data emphasize the need for combined experimental- and system-level analyses to comprehensively disentangle and reveal miRNA functions, including their involvement in the neurogenesis of diverse neuronal cell types found in the human brain.

INTRODUCTION

The human brain comprises more than 300 neuronal cell types, with an undetermined number of subtypes. Their underlying developmental programs are mostly unknown. In recent years, miRNAs have been identified as an important part of neurogenesis (Åkerblom and Jakobsson, 2014). miRNAs bind in a sequence-specific manner to mRNA transcripts and thereby negatively interfere with the simultaneous translation of multiple target transcripts by annealing predominantly at the 3′ untranslated region (UTR). miR-124 is one of the most abundant miRNAs in the brain and is associated with processes such as neurogenesis (Lagos-Quintana et al., 2002; Landgraf et al., 2007), cancer (Silber et al., 2008; Taniguchi et al., 2015), and the control of synaptic functions in mature neurons in health (Dutta et al., 2013; Hou et al., 2015; Rajasethupathy et al., 2009) and disease (Fang et al., 2012; Lukiw, 2007) including cognitive impairment (Gascon et al., 2014; Yang et al., 2014).

It remains unclear if miR-124 is controlling the initiation of neuronal differentiation (Åkerblom et al., 2012; Cao et al., 2007; Cheng et al., 2009; Conaco et al., 2006; Makeyev et al., 2007; Visvanathan et al., 2007; Yoo et al., 2009, 2011) or the maturation and survival of the differentiated neurons (Franke et al., 2012; Gu et al., 2014; Li and Ling, 2017; Sanuki et al., 2011; Xue et al., 2016; Yu et al., 2008), or both. Knockdown studies using sponge approaches (Åkerblom et al., 2012) or antisense constructs (Cheng et al., 2009; Conaco et al., 2006) resulted in decreased neurogenesis. However, transcriptomic alterations from knockdown approaches are mild and their interpretation difficult. Gain-of-function experiments resulted in elevated neuronal differentiation of mouse embryonic stem cells (Krichevsky et al., 2006), HeLa cells (Lim et al., 2005), and glioblastoma cells (Silber et al., 2008). Forced expression of miR-124 in combination with miR-9 converts human fibroblasts into neuronal cell types (Yoo et al., 2011), highlighting miR-124's cell fate-determining potency. Therefore, miR-124 was suggested to play a key role during neuronal differentiation by promoting the exit from the cell cycle, downregulating pro-proliferative genes, and activating

 © 2018 The Author(s). Published by Elsevier Inc.
This is an open access article under the CC BY license (http://creativecommons.org/licenses/by/4.0/).

neuron-specific genes (Conaco et al., 2006; Makeyev et al., 2007; Visvanathan et al., 2007).

However, previous knockout studies in mouse models were incomplete as not all three miR-124 paralogs, six alleles in total, coding for identical mature miRNAs were ablated (Sanuki et al., 2011). There is also a high heterogeneity of neuronal progenitor cells *in vivo*, impeding studies of miR-124 in defined cell types (Yaworsky and Kappen, 1999). Pooling heterogeneous cell types that differ in their coding and non-coding transcriptome likely results in incomplete views on miR-124's complex regulatory role with 4,024 computationally predicted targets as well as additional potential non-canonical binding events (Chi et al., 2012; Moore et al., 2015). So far, most studies have experimentally validated only a single or very few miR-124 targets at once. It remains unclear how many miR-124 targets are simultaneously regulated within a cell and what their orchestrated impact is—direct and also indirect—via gene regulatory cascades. Therefore, it is essential to investigate miR-124's functions in a well-defined, homogeneous, and complete knockout model to overcome the above-mentioned limitations.

We used a human induced pluripotent stem cell (hiPSC)-based model system to mimic neurogenesis of bipolar neurons under controlled and reproducible conditions (Busskamp et al., 2014a). By CRISPR/Cas9 genome editing (Cong et al., 2013; Mali et al., 2013), a full miR-124 knockout of all six genomic copies was generated. By forced transcription factor (TF) induction, we rapidly and robustly induced neuronal differentiation in wild-type (WT) and ΔmiR-124 cells. Subsequently, we performed an in-depth molecular, cellular, and physiological characterization of the ΔmiR-124 and isogenic WT lines, which revealed altered morphological and functional features, different neurotransmitter specification, and decreased long-term viability. We performed RNA-interacting protein immunoprecipitation (IP) and subsequent sequencing (RIP-seq) (Malmevik et al., 2015; Petri et al., 2017) to capture active miRNAs and their mRNA targets bound to Argonaute-2 (AGO2), identifying 98 miR-124-regulated targets in parallel.

Cross-correlating RIP-seq experiments, miRNA quantifications, and time course RNA sequencing (RNA-seq) enabled us to identify other miRNA species with increased activity upon miR-124 deletion. Since we were mainly interested in functional differences between TF networks in WT and ΔmiR-124 neurons, we applied an advanced weighted topological overlap (wTO) network analysis (Berto et al., 2016; Nowick et al., 2009), followed by a co-expression differential network analysis (Gysi et al., 2018). Using this computational approach, we were able to detect similarities and specific differences in WT and ΔmiR-124 neurogenesis at the level of genes and their regulatory connections, including the impacts on neurogenesis of the uncharacterized TF *ZNF787*. Our results highlight the complexity of the downstream effects of experimental miRNA manipulations.

RESULTS

Generation of a Human iPSC-Based miRNA-124 Knockout Model

We wanted to study miR-124 functions with an emphasis on neurogenesis in a robust, well-defined, and inducible neuronal cell line derived from hiPSCs in which miR-124 is highly expressed (Busskamp et al., 2014a). This so-called inducible-neurogenin cell line (iNGN) consists of stably integrated expression cassettes coding for the reverse tetracycline-controlled transactivator 3 (rtTA3) and the TFs neurogenin-1 and -2 (Neurog1/2), which are driven from the doxycycline-inducible promoter (Figure 1A). Hence, iNGN cells are hiPSCs, in which doxycycline administration triggers neurogenesis into a homogenous population of bipolar-shaped neurons within 4 days post induction (dpi), which can be cultured for months and acquire robust functional properties (Lam et al., 2017).

To completely delete miR-124 in iNGN cells, we applied the CRISPR/Cas9 technology (Ran et al., 2013) to knock out all three genomic loci, six alleles in total, which encode for the identical mature miR-124 sequence. We designed four unique gRNAs, which in pairs flanked all miR-124 loci (Figure 1B; STAR Methods). Transient expression of the Cas9 protein and gRNA pairs in uninduced iNGN cells resulted in complete genomic deletions of all miR-124 loci (Figure 1C). We thoroughly compared a monoclonal homogeneous ΔmiR-124 line with an isogenic monoclonal iNGN control (WT) and detected no off-target effects in top-predicted genomic sites by sequencing or chromosomal alterations when karyotyping in the ΔmiR-124 cell line (Figure S1; STAR Methods). We observed miR-124 expression neither in the ΔmiR-124 line as iPSCs (0 dpi) nor in neurons at 4 dpi (Figure 1D), whereas in WT we measured an 18-fold increase in miR-124 expression: this demonstrates the successful generation of a complete miR-124 knockout cell line.

miR-124 Loss Leads to Molecular and Phenotypic Changes in Induced Neurons

We then characterized the effects of miR-124 deletion on neurogenesis by inducing neuronal differentiation in ΔmiR-124 and WT cells. In both lines, we detected specific neuronal markers such as MAP2, TUBB3, DCX, NEUN, and NCAM1 using immunocytochemistry at 4 dpi, indicating the presence of neurons (Figures 2A and S2A). Next, we quantified representative protein levels of pluripotency and neuronal lineage commitment by flow cytometry. In both cell lines, the TRA-1-60 signal, a marker for pluripotency, decreased, whereas the NCAM1 signal, a neuronal marker, increased during the course of differentiation. In ΔmiR-124 cells, signals for TRA-1-60 ($p < 0.01$ for 2 dpi and 3 dpi) and NCAM1 ($p < 0.05$ for 1 dpi, 2 dpi, and 3 dpi) were significantly lower (Figure 2B). Still, the ΔmiR-124 cells clearly differentiated into neurons after induction of neurogenesis, suggesting that neurogenesis in iNGN cells was independent of miR-124.

Microscopic analysis revealed that fewer ΔmiR-124 cells became bipolar-shaped (i.e., normal WT morphology): the bipolar neuronal fraction significantly decreased from 96.6% ± 0.3% in WT to 71.3% ± 4.3% ($p < 0.05$) in ΔmiR-124 neurons, with many ΔmiR-124 cells showing a more complex morphology at 4 dpi (Figures 2C and 2D). We analyzed neurite outgrowth, which can correlate with differences in morphology, by time-lapse microscopy: the outgrowth velocity was significantly reduced ($p < 0.05$) to 0.66 ± 0.13 μm/min in ΔmiR-124 neurons compared to 1.02 ± 0.11 in WT (Figures 2E and S2E), whereas overexpressing (OE) miR-124 in ΔmiR-124 neurons reset the morphology to 97.6% ± 0.8% bipolar shape ($p < 0.001$; Figures 2F and S2B–S2D). In addition, we found neurite outgrowth velocities comparable to WT levels in miR-124 supplemented ΔmiR-124 cells

OPEN
ACCESS
CellPress

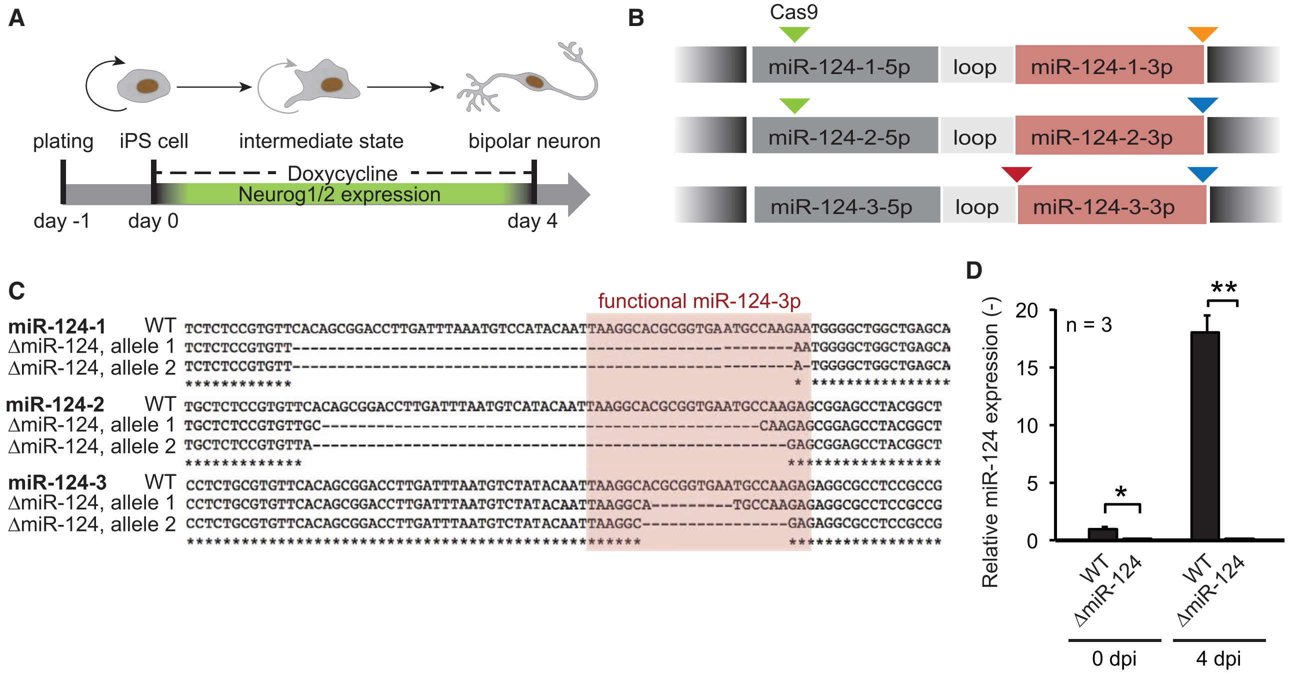

Figure 1. Generation of an miR-124 Knockout Stem Cell Line

(A) Schematic illustration of the inducible neurogenin-1/2 (iNGN) stem-cell line in which neurogenesis from hiPSCs to post-mitotic neurons can be triggered within 4 days.

(B) CRISPR-Cas9 miR-124 depletion strategy. All three miR-124 loci were targeted by four specific 5′- and 3′-flanking gRNAs as indicated by differently colored triangles.

(C) Sanger sequencing results of miR-124 genomic loci. All six alleles were disrupted.

(D) qRT-PCR analysis indicated the lack of miR-124 expression in ΔmiR-124 cells, tested in iPSCs (0 dpi) and neurons (4 dpi). Statistical analysis was performed using unpaired Student's t tests with *$p \leq 0.05$, **$p \leq 0.01$. Data are represented as mean ± SEM.

See also Figure S1.

(from 0.37 ± 0.11 μm/min to 1.11 ± 0.12 μm/min; $p < 0.001$; Figures 2G and S2F). Our data suggest that miR-124 deletion led to phenotypic differences, which were reversible. Thus, miR-124 was dispensable for the general neuronal differentiation program in WT and ΔmiR-124 iNGN cells but resulted in an increased and reversible morphological heterogeneity. We confirmed that neurogenesis was independent of the miR-124 knockout clone (Figure S2M), the underlying hiPSC line (Figures S2K–S2N), and the TF-overexpression neuronal induction protocol, since an alternative small molecule-based induction resulted in neurons with equal efficiency within 21 dpi (Figures S2H–S2J) (Reinhardt et al., 2013).

ΔmiR-124 Neurons Become Electrophysiologically Active

As general neurogenesis was unaffected, we questioned whether ΔmiR-124 iPSCs undergo functional neuronal maturation. We tested the electrophysiological properties of WT and ΔmiR-124 neurons and found that all WT cells (n = 12) fired action potentials (APs) and that one fired multiple APs upon current injection at 7 dpi (Figure 2H). At 14 dpi, 92% (n = 13) of WT cells generated APs, with 31% AP firing trains. ΔmiR-124 cells generated APs as well: 92% (n = 13) at 7 and 14 dpi, with one cell firing multiple APs at 14 dpi. Both cell lines showed similar capacitance and membrane potential, suggesting similar cell sizes and passive conductive properties (Figures 2I and 2J). However, ΔmiR-124 cells were characterized by a lower voltage-dependent peak current in a voltage range from −30 mV to +10 mV at 14 dpi ($p < 0.05$; Figures 3K–3M) but showed similar steady currents (Figures 2L and 2M) compared to WT cells. This peak current shift in ΔmiR-124 neurons (Figures 2L and 2M) indicated reduced sodium channel expression levels or altered channel properties. In summary, our data suggest that both WT and ΔmiR-124 neurons became functional, although with differences in specific properties, suggesting that miR-124 has only a minor impact on neuronal maturation.

Transcriptomic Differences in WT and ΔmiR-124 Neuronal Populations

In order to assess how the phenotypic differences were reflected at the transcriptomic level, we used RNA-seq to assess transcriptomic profiles of ΔmiR-124 and WT cells over the course of neuronal differentiation between 0 dpi and 4 dpi (n = 7 biological replicates for each cell line per time point). In line with our immunohistochemical analysis, key neuronal markers (*MAP2*, *TUBB3*, *PAX6*, *SOX1*, and *RBFOX3*) were expressed in ΔmiR-124, with small but significantly altered levels ($p_{adj} < 0.05$ and >1.5-fold) compared to WT (Figure 3A). As expected, pluripotency genes decreased over 4 days. Sodium channels were significantly decreased ($p_{adj} < 0.05$ and >1.5-fold)

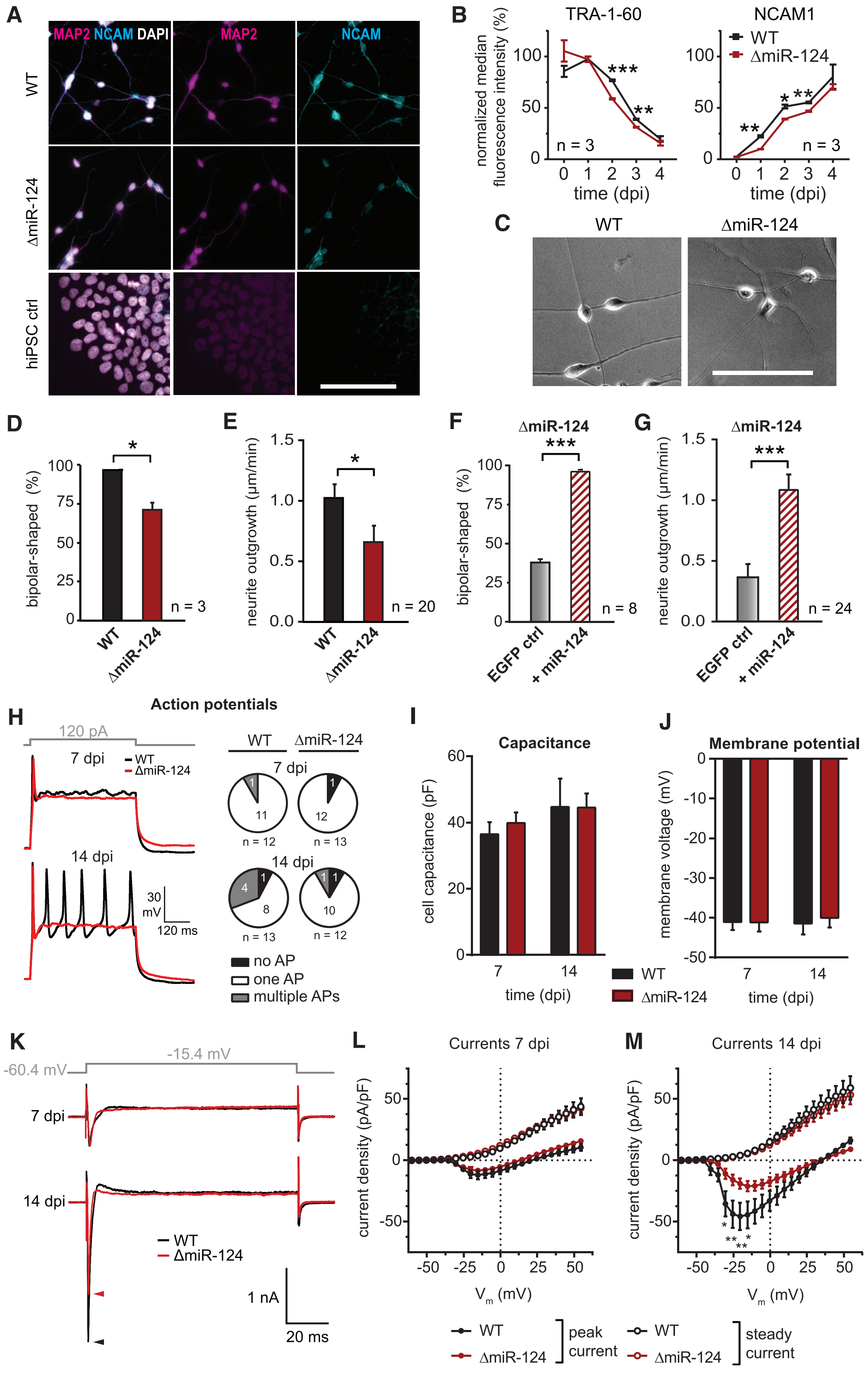

Figure 2. Phenotypic Characterization of ΔmiR-124 versus WT Cells

(A) Immunostainings of indicated neuronal markers at 4 dpi and 0 dpi (hiPSC control).

(B) Loss of pluripotency (TRA-1-60) and neural lineage commitment (NCAM1) 0–4 dpi measured by flow cytometry using the surface markers indicated.

(legend continued on next page)

(*SCN2A*/NaV1.2) or unchanged (*SCN8A*/NaV1.6), which may explain the differences in peak currents (Figure 2M). WT iNGN neurons have previously been characterized as co-transmitting glutamate and acetylcholine as both neurotransmitter synthesis and secretion pathways are upregulated at 4 dpi (Busskamp et al., 2014a). Here, subtype-specific markers differed significantly (p_{adj} < 0.05 and >1.5-fold) from a glutamatergic specification (*VGLU1*/*VGLU2*/*NMDA1*) in WT to cholinergic in ΔmiR-124 cells (*CHAT*; Figure 3A).

We studied the overall changes in gene expression in the absence of miR-124 by analyzing differential gene expression patterns between WT and ΔmiR-124 over the course of neurogenesis (Figure S3A). We found 2,884 genes to be differentially expressed at 0 dpi, 10,820 at 1 dpi, 10,897 at 2 dpi, 15,196 at 3 dpi, and 15,731 at 4 dpi (p_{adj} < 0.05; Figure S3A). These genes were involved in differentiation, cell adhesion, morphogenesis, and cell division.

Next, we analyzed the transcriptional differentiation course of WT and ΔmiR-124 cells using a principal-component analysis (PCA) of RNA-seq data (n = 7) (Figure 3B). Comparing the greatest variance dimensions, individual replicates showed minimal variance per time point. At 0 dpi, WT and ΔmiR-124 samples clustered together, suggesting that both cell lines started from a similar cellular ground state. From 1 dpi on, WT and ΔmiR-124 samples diverged and took different paths during differentiation. There was no common endpoint convergence at 4 dpi, suggesting the presence of two distinct populations.

Using their transcriptomic profiles, we categorized WT and ΔmiR-124 cells from different brain regions and developmental stages. The expression profiles of 4 dpi WT and ΔmiR-124 neurons were separately correlated with the Allen BrainSpan Atlas dataset, which includes RNA-seq data from mixed cell populations acquired from 16 brain structures between 8 post-conception weeks (pcw) and 40 years of age (Miller et al., 2014). We detected the highest correlation coefficients (Pearson correlations > 0.3) between 8 and 37 pcw, suggesting that WT and ΔmiR-124 neurons most closely represented prenatal neurons (Figures 3C–3E; STAR Methods). Focusing on spatial information, WT neurons correlated best with expression data from the amygdaloid complex, cerebellar cortex, and hippocampus, while ΔmiR-124 neurons were most similar to the cerebellar cortex, hippocampus, and striatum (Pearson correlations > 0.39) (Figures 3D and 3E). The differences between WT and ΔmiR-124 neurons were also detected at later time points (7 dpi and 14 dpi, n = 3 biological replicates for each cell line per time point). WT samples correlated also to prenatal cortical areas (Figures S3B and S3C) and had higher correlation scores, suggesting that these neurons are more likely to be found in the human brain.

AGO2 Complex Precipitation Identifies Active miRNA-mRNA Pairs

How is miR-124 linked to the aforementioned morphological and functional differences between WT and ΔmiR-124 cells? As miRNAs repress gene expression, we expected direct miR-124 targets to be upregulated after miR-124 deletion. Computational predictions of miRNA target genes necessarily remain inconclusive because one miRNA can bind to thousands of targets and most mRNAs have multiple miRNA binding sites. There are 4,024 annotated human transcripts with miR-124 binding sites (according to miRWalk with RNA22, Targetscan, and miRanda databases) (Dweep et al., 2011; John et al., 2004; Lewis et al., 2005; Miranda et al., 2006). Furthermore, the impact of miRNAs on target gene repression has been found to be mostly moderate (Baek et al., 2008; Guo et al., 2010b). Just focusing on mRNA expression data may most likely be insufficient for a systematic assessment of biologically active miR-124 targets as it would also deliver many secondary and therefore indirect targets. Thus, we used AGO2-RIP-seq to identify active miRNAs and their targets in parallel. The physical binding of miRNA to their mRNA targets occurs in the RNA-induced silencing complex (RISC), in which AGO2 is one of the central enzymes (Liu et al., 2004). IP of AGO2 includes bound miRNA and mRNA molecules that can be identified by subsequent RNA-seq: we performed this for both AGO2-IP and whole-cell samples (pre-IP whole-cell RNA samples). We then conducted a differential AGO2-precipitated concomitance analysis of mRNA and miRNA molecules from WT and ΔmiR-124 samples. Subsequently, we filtered for active miR-124 targets (Figure 4A). For quality control, we checked for equal amounts of AGO2 protein levels between samples (Figures S4A and S4B) and a decrease in mitochondrial mRNAs after IP (Figure S4C): these were expected as mitochondrial RNA is not bound to the RISC. Furthermore, miRNA, snoRNA, and snRNA levels were equal between WT and ΔmiR-124 in whole-cell samples (Figure S4D).

Reads from AGO2-RIP-seq were mapped to the reference genome, and regions surpassing a read threshold (see STAR

(C) Representative images of WT and ΔmiR-124 neurons at 4 dpi with differing morphology.
(D) Quantification of bipolar morphology for WT and ΔmiR-124 neurons at 4 dpi. n, number of independent samples.
(E) Quantification of neurite outgrowth velocities of WT and ΔmiR-124 neurons. n, number of neurites measured.
(F) Quantification of bipolarity of ΔmiR-124 with and without miR-124 overexpression at 4 dpi. The overexpression of miR-124 rescued the typical bipolar morphology (see also STAR Methods). n, number of independent samples.
(G) Quantification of neurite outgrowth velocities of ΔmiR-124 neurons at 5 dpi with and without miR-124 overexpression. The impairment was rescued by miR-124 overexpression. n, number of neurites measured.
(H) Example traces and action potential (AP) quantification for 7 and 14 dpi of WT and ΔmiR-124. APs were induced by current injection.
(I) Quantification of cell capacitance of WT and ΔmiR-124 neurons at 7 and 14 dpi, indicating similar cell sizes for each time point.
(J) Quantification of membrane voltage of WT and ΔmiR-124 at 7 and 14 dpi, an indicator for potassium and proton channel activity.
(K–M) Quantification of peak and steady currents with example traces (K) at 7 and 14 dpi. Peak currents are sodium-channel dominated, whereas steady currents are potassium-channel dominated.
Scale bars, 100 μm. Sample number, n, refers to the number of biological samples (B, D, and F), number of analyzed neurites (E and G), and number of recorded cells as stated in (H) (H–M). Statistical analysis, unpaired Student's t tests for two-group comparisons and Holm-Sidak correction for multiple comparisons with *p ≤ 0.05, **p ≤ 0.01, ***p ≤ 0.001. Data are represented as mean ± SEM.
See also Figure S2.

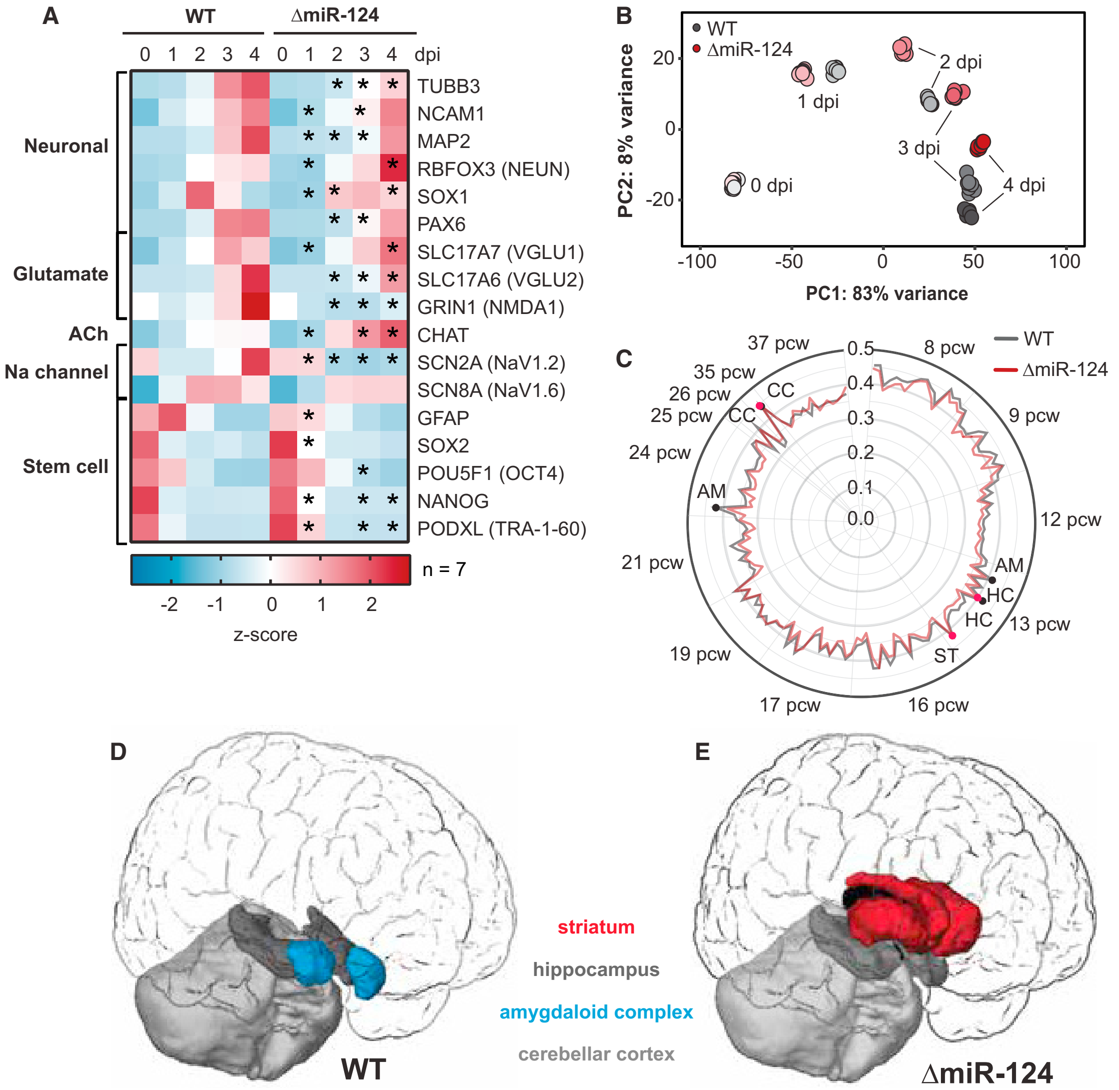

Figure 3. Transcriptomic Characterization of WT and ΔmiR-124 Neurons

(A) Heatmap of differentially expressed genes (RNA-seq, z scores, n = 7 biological replicates) for WT and ΔmiR-124 cells for selected neuronal and pluripotency markers over the time course of differentiation. Asterisks mark significant differences between the cell lines (*$p_{adj} < 0.05$ and fold difference ≥ 1.5).

(B) Principal-component analysis showing the greatest variance dimensions of time course expression data from WT and ΔmiR-124 cells.

(C) Correlation analysis of WT and ΔmiR-124 neurons to the developing human brain. Pearson correlation of the 1,000 most differentially expressed genes compared to the transcriptomic Allen BrainSpan Atlas dataset for the developmental stages from 8 to 37 postconceptional weeks (pcw). Abbreviations represent brain regions: AM, amygdaloid complex; CC, cerebellar cortex; ST, striatum; HC, hippocampus.

See also Figure S3.

(D) Graphical illustration of the Allen BrainSpan Atlas analysis (C) for WT neurons at 4 dpi.

(E) Graphical illustration of the Allen BrainSpan Atlas analysis (C) for ΔmiR-124 neurons at 4 dpi.

Methods) were considered as peaks. Peaks specific to WT samples were filtered out of the WT and ΔmiR-124 datasets. We used transcript expression levels from whole-cell samples to normalize RIP-seq signals (Sedlyarov et al., 2016; see STAR Methods). 2,338 genes had significant peak region signals in the WT but not in the ΔmiR-124 samples. We also analyzed for differential gene expression, finding 1,909 genes with significantly increased expression levels in WT compared to ΔmiR-124 cells ($p_{adj} < 0.05$; Figure 4B). The AGO2-RIP-seq signals did not recapitulate the distribution of WT and ΔmiR-124 whole cell RNA-seq samples, suggesting a different RISC-complex occupation of available mRNAs (Figure S4F). By intersecting these genes, we identified 127 transcripts with reduced 3′ UTR peaks (AGO2 IP) and increased expression in whole-cell ΔmiR-124 samples, of which 98 (77.2%) were predicted to be miR-124 targets (Figures 4B and 4C). 38 of the 98 targets had previously been validated (Figure 4C; taken from miRTarBase; marked in red) (Hsu et al., 2011). We used luciferase reporter assays to further test 45 3′ UTRs, confirming that 43 filtered targets were indeed miR-124 targets (Figures 4D and 4E). In summary,

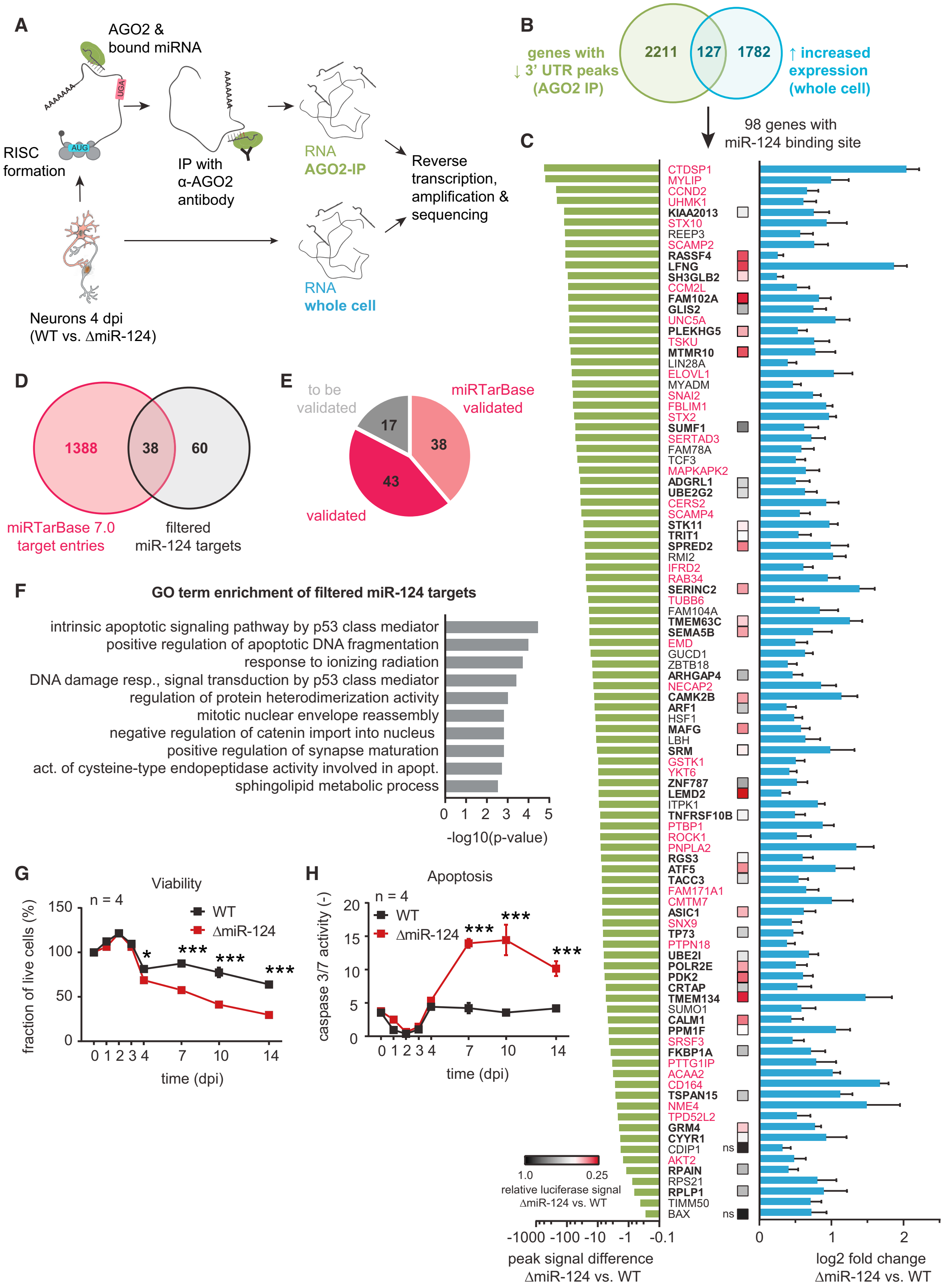

Figure 4. Parallel Identification of miR-124 Targets by AGO2-RIP-Seq Analysis

(A) Schematic representation of the AGO2-RIP-seq experiment using AGO2 precipitation and subsequent sequencing of mRNAs and miRNAs to identify active pairs.

(legend continued on next page)

the AGO2-RIP-seq analysis enabled a comprehensive parallel identification of 98 high-confidence active miR-124-targets (81/98 validated) from the 4,024 annotated targets in iNGN neurons (Figure 4E). However, this number most likely underestimated the complete target repertoire, as our filters were quite stringent and we did not capture any targets that were only regulated at the protein level.

Increased miR-124 Target Expression Leads to Decreased Neuronal Viability

We further analyzed the 98 high-probability targets to assess their involvement in biological processes using a Gene Ontology (GO) term analysis (Figure 4F). We detected that terms associated with synaptic maturation and apoptosis were enriched among those target genes at 4 dpi (Figure 4F). As ΔmiR-124 cells became electrically active over time but had significantly increased transcriptomic traces of apoptosis, we experimentally investigated cell viability and apoptosis rates (caspase 3/7 activity) (Walsh et al., 2008) in WT and ΔmiR-124 neurons over 14 days in culture (Figures 4G and 4H). ΔmiR-124 cell viability was significantly decreased from 4 dpi onward ($p < 0.05$), and caspase 3/7 activity was significantly enhanced ($p < 0.05$) in ΔmiR-124 compared to WT. The viability of ΔmiR-124 cells over time was significantly increased by miR-124 supplementation ($p < 0.05$; Figure S4E), suggesting that miR-124 expression is directly linked to neuronal survival. The 98 identified miR-124 targets remained increased in expression at 7 and 14 dpi (Figure S4F), and the associated GO terms further supported miR-124's impact on neuronal survival, specification, and maturation (Figures S3D and S3E).

miRNA Transcriptome Dynamics upon miR-124 Depletion

We next investigated how the global miRNA expression profile reacted to the depletion of a highly abundant miRNA species such as miR-124. Previously, we had found that miR-124 represented about 80% of the miRNA profile in iNGN cells at 4 dpi (Busskamp et al., 2014a). It has also been shown that a full miRNA depletion, by knocking out key enzymes of the miRNA processing machinery, can lead to downregulation of RISC protein members, including AGO2 protein levels (Busskamp et al., 2014b; Martinez and Gregory, 2013). This results in an overall decrease in miRNA-mediated regulation. Therefore, we analyzed whether the deletion of miR-124 in iNGN cells also resulted in an overall decrease in the miRNA machinery. However, we did not detect any significant decreases in AGO2 protein levels (Figures S4A and S4B). Therefore, miRNAs were likely still able to exert their functions. In addition, we performed nCounter miRNA quantifications of WT and ΔmiR-124 samples at 0 dpi and 4 dpi (n = 3 replicates per sample and time point) and found similar count distributions at 0 dpi (Figure 5A). However, at 4 dpi, we detected a shift toward higher counts in ΔmiR-124 samples, suggesting that other miRNAs in ΔmiR-124 neurons were increased in numbers (Figure 5B).

Next, we looked at miRNAs that were differentially expressed in AGO2-RIP-seq samples. As expected, we found miR-124 to be significantly downregulated at 4 dpi (Figure S5A). We put an emphasis on upregulated miRNA species as these molecules likely took over miRNA regulatory functions in ΔmiR-124 cells (Figures 5C and S5A). As amplification biases occur massively during small RNA-seq (sRNA-seq) library preparation (Hafner et al., 2011; Linsen et al., 2009; Tian et al., 2010), we aimed at combining our differential expression analysis of AGO2-RIP-seq and sRNA-seq data with nCounter quantifications to filter for both upregulated and sufficiently expressed miRNAs that took over regulatory functions in the absence of miR-124. Eight miRNA species had increased levels in AGO2-RIP-seq and whole cell sRNA-seq ΔmiR-124 samples compared to WT and more than 200 nCounter counts at 4 dpi (Figure 5D). Furthermore, these miRNAs showed enhanced association in the AGO2 complex by normalizing to whole-cell signals and activity ranking within the AGO2 complex (Figure S5B; STAR Methods). Differences in miRNA expression ranking between WT and ΔmiR-124 samples suggested new biologically active miRNA regulations within the RISC in the absence of miR-124. We looked for validated targets of these eight miRNAs and highlighted 12 genes with increased peak signal differences (AGO2-RIP-seq) and significantly decreased expression levels in whole cell samples in ΔmiR-124 samples compared to WT. Our data indicated that these genes became regulated by other miRNAs in the absence of miR-124. Performing a GO term analysis on their validated targets (miRTarBase) further suggested involvement in neuronal morphology (Figure S5C).

Taken together, we revealed that the depletion of miR-124 resulted in dynamic changes of actively expressed miRNAs taking over regulatory actions on their corresponding target repertoire, resulting in indirect effects on the coding and non-coding transcriptome during neurogenesis.

(B) Differentially expressed genes were filtered as shown in the Venn diagram. Transcripts with less 3′ UTR signal in AGO2-IP data in ΔmiR-124 samples were intersected with significantly upregulated transcripts in the whole-cell samples. 127 transcripts overlapped, of which 98 were annotated miR-124 targets.

(C) 98 high-probability miR-124 targets filtered for 3′ UTR peak signal decrease (left) and increase in expression (right). Data are presented as mean ± log_2-fold change standard error (lfcSE). Experimentally validated targets according to miRTarBase are indicated in red. 43 additional targets were validated by luciferase reporter assays; color code indicates relative luciferase signal reduction upon miR-124 overexpression.

(D) Venn diagram showing miRTarBase entries intersected with the 98 filtered miR-124 targets.

(E) Pie chart, summarizing previous (miRTarBase), experimentally validated (luciferase reporter assays) and untested miR-124 targets.

(F) GO term enrichment analysis of filtered miR-124 targets indicating their involvement in apoptosis and synaptic maturation.

(G) Assessment of cell viability from 0 to 14 dpi. The percentage of live cells was measured using the ApoToxGlo assay. The ratio between fluorescence signal for live cells (AFC) and total cell fluorescence (AFC + R110) was examined and normalized to 100% for 0 dpi.

(H) Assessment of apoptosis from 0 to 14 dpi. Luciferase activity corresponding to Caspase 3/7 activity was measured using the ApoToxGlo assay. Luciferase activity was normalized to the live cell fluorescence (AFC).

Statistical analysis, unpaired Student's t tests with Holm-Sidak correction for multiple comparisons with $^*p \leq 0.05$, $^{***}p \leq 0.001$. Data are represented as mean ± SEM.

See also Figure S4.

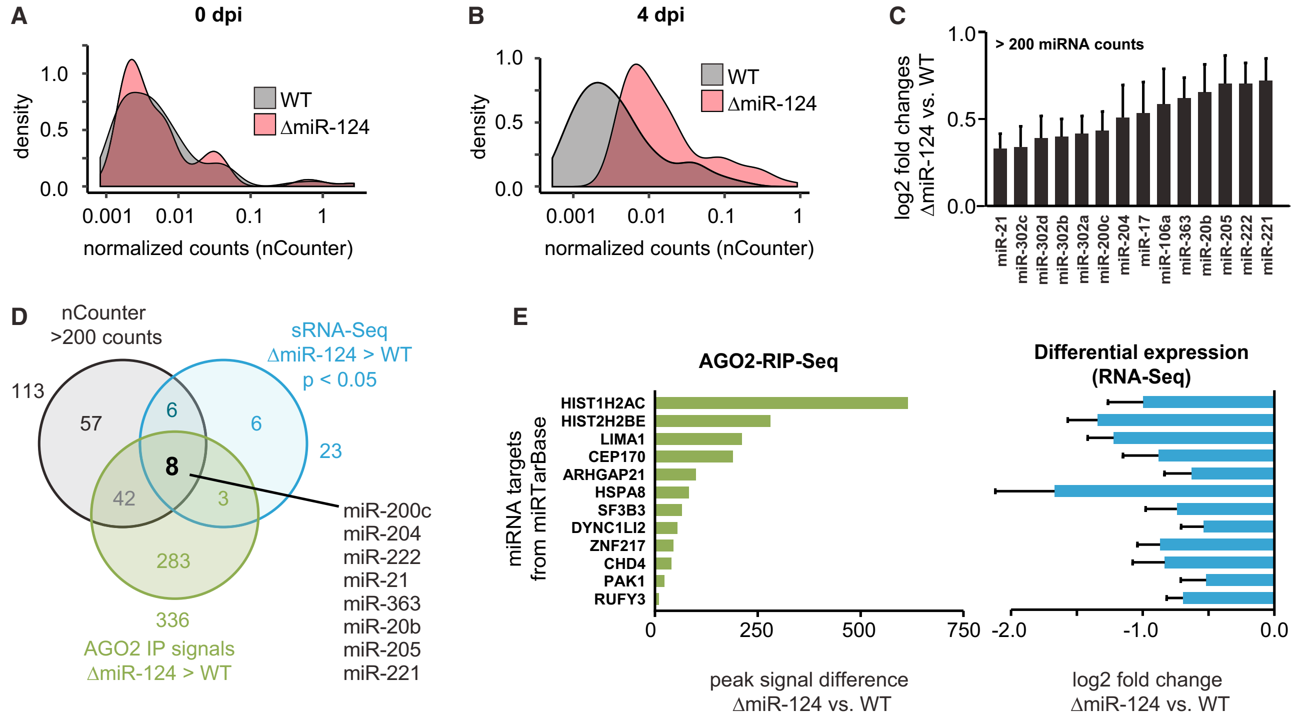

Figure 5. Dynamic Co-regulatory Effects of Other miRNAs in the Absence of miR-124

(A and B) Density plots of normalized nCounter counts for WT and ΔmiR-124 at 0 dpi (A) and 4 dpi (B).

(C) Significant differentially expressed miRNAs from sRNA-seq with nCounter > 200 counts.

(D) Venn diagram of highly abundant miRNAs (nCounter > 200 counts) that were upregulated in ΔmiR-124 and showed higher AGO2 signals in ΔmiR-124 compared to WT.

(E) Increased AGO2-RIP signals and decreased $\log_2$-fold changes for miRNA targets shown in (D) that were previously validated (miRTarBase). Data are represented as mean ± SEM.

A Network of Transcription Factors Is Influenced by miR-124

A significant fraction of the identified miR-124 targets (24/98, $p < 0.05$; χ^2 test) coded for TFs, suggesting that miRNA-124 exerts much of its impact via influencing gene regulatory cascades involving many TFs. Measuring indirect miRNA effects is not trivial (Hill et al., 2014) but essential to understanding the full spectrum of miRNA regulation. We conducted a time-lapse network analysis focusing on the 24 miR-124 targets coding for TFs to understand the miR-124 regulatory network underlying neurogenesis.

For each time point and, separately, for WT and ΔmiR-124 cells (n = 7 for each time point), we identified genes that correlated with the 24 TFs. Correlated genes represent potential target genes or interaction partners of TFs. To reveal how similar TFs were to their correlated target genes, we calculated wTO networks for all expressed TFs for each time point for WT and ΔmiR-124 cells separately (Figures 6A, S6A, S7A, and S7B). Nodes in these wTO networks represent TFs, and they are connected by a link if they share a significant number of correlated genes, that is, are likely acting together in regulating their target genes. One advantage of wTO networks is that they result in more robust definitions of connections and interactions among genes than simple correlation networks because they suppress false positive inferences (Carlson et al., 2006; Gysi et al., 2017; Nowick et al., 2009; Ravasz et al., 2002). In contrast to the widely used weighted gene co-expression network analysis (WGCNA) (Zhang and Horvath, 2005), the wTO network that we have developed accommodates both positive and negative correlations, which are essential for analyzing TFs as they have both enhancing and repressing functions (Gysi et al., 2017; Nowick et al., 2009). Furthermore, our method also assigns p values to each link, resulting in a high-accuracy network based on seven replicates, which is important for comparing networks with high confidence (Gysi et al., 2017). At 0 dpi, WT and ΔmiR-124 networks were identical. They started to differ from 1 dpi, peaked in differences at 3 dpi (Figure 6A), and decreased in differences at 4 dpi (Figures S7A and S7B). To reveal the contrast between the WT and ΔmiR-124 networks, we subtracted the wTO of ΔmiR-124 from the wTO of WT, resulting in differential networks (Figures 6A and S7A, top). In WT cells, a number of links were reactivated at different time points, while in the ΔmiR-124 cells most links were unique, suggesting the presence of different regulatory modes and global changes after miR-124 loss (Figure S7C). We classified the differentially expressed TFs (14.95%, n = 3,145) using a self-organizing map (SOM) algorithm (Figures 6B and S7D). The SOM categories with a steady increase in differential expression over time were considered to be the best candidates for also being influenced by miR-124, as they were expected to be upregulated in the absence of the negative regulator (Figures 6B and S7D). Most miR-124 targets obtained from RIP-seq filtering (Figures 4A–4C) appeared in

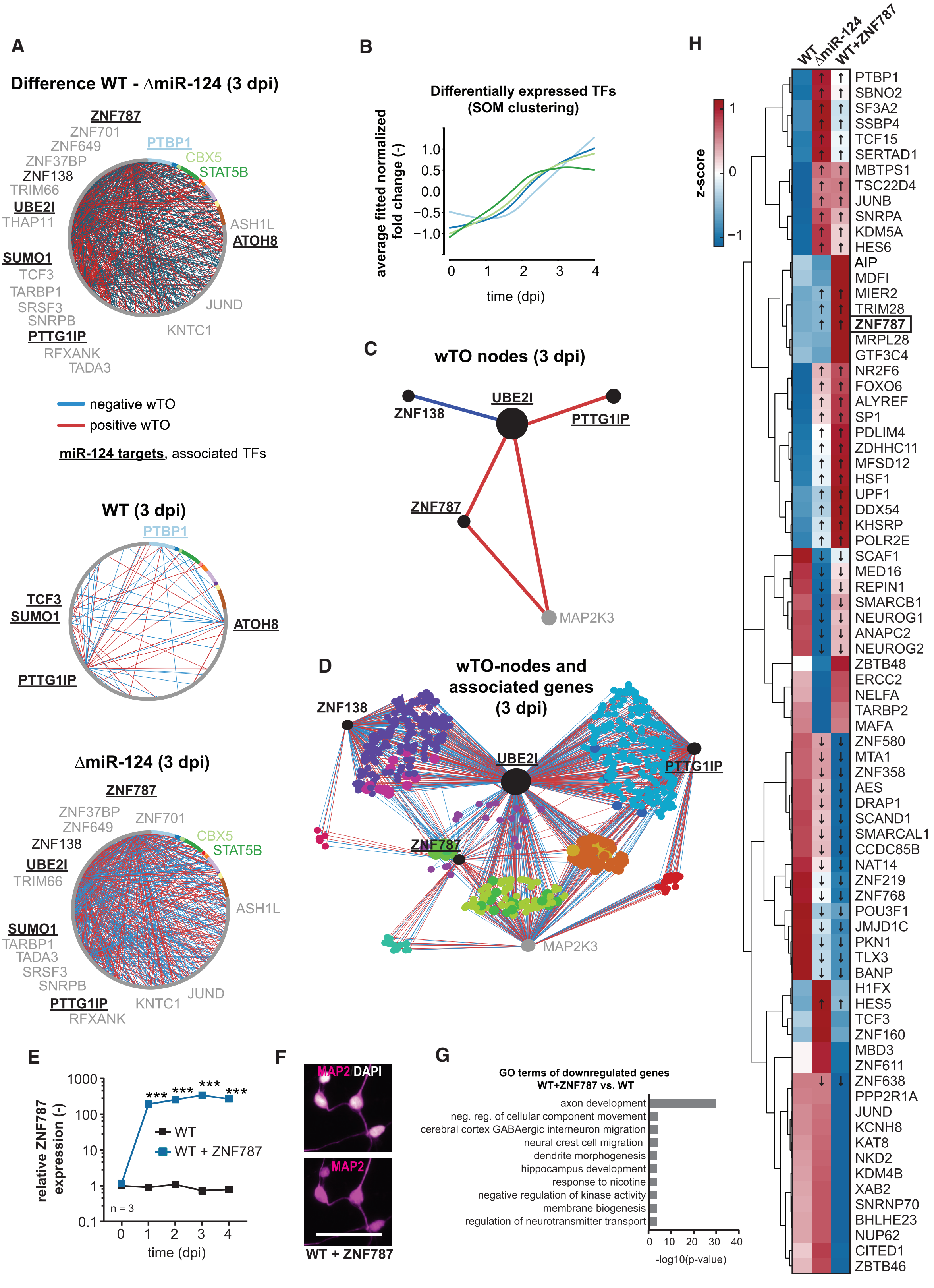

(legend on next page)

the monotonically increasing SOM categories, which is in line with the lack of repressing miR-124 (Figures 4B and S7D): the targets previously described included *PTBP1*, which was identified as an important factor within our co-expression network (Figure 6A). Nodes including *GLIS2*, *SERTAD3*, and *TP73* appeared to be very important as these genes fulfilled all criteria: they were filtered and validated targets (Figure 4C), were top hits in the network analysis, and followed a rising trend in the SOM clustering.

We focused specifically on 3 dpi, as we detected most network activity at this time point (Figures 6A, S7C, and S7D). We also found some miR-124 targeted TFs with unknown functions (*ZNF787*) as well as human-specific indirectly targeted ones (*ZNF138*) within the dense 3 dpi network of the ΔmiR-124 samples (according to Uniprot; Nowick et al., 2011). Exemplarily, individual nodes around the miR-124 target *ZNF787* and connected *ZNF138* (Figure 6C) as well as their associated genes (Figure 6D) were extracted from our wTO analysis. This visualization emphasizes how embedded *ZNF787* was within the gene regulatory network upon miR-124 deletion at 3 dpi. Next, we aimed at perturbing the *ZNF787* node by OE *ZNF787* robustly in WT iNGN cells (Figure 6E). WT-ZNF787-OE cells underwent neurogenesis and were positive for the neuronal marker MAP2 (Figure 6F). We performed GO term analyses on differentially expressed genes between WT and WT-ZNF787 OE ($n = 3$ biological replicates, 4 dpi). Specifically, focusing on downregulated genes, many neuronal biological processes were significantly inhibited (Figure 6G). Hence, our data indicated that *ZNF787* represents a neuronal feature repressor. Looking at *ZNF787*-associated genes derived from our wTO analysis, corresponding expression levels massively differed between WT, ΔmiR-124, and WT-ZNF787-OE (Figure 6H). In particular, 51 out of 78 ZNF787-associated genes showed a similar expression trend for ΔmiR-124 and WT-ZNF787-OE in comparison to WT (Spearman correlation, $R = 0.498$ with $p < 0.001$; Pearson correlation, $R = 0.277$ with $p = 0.01$). This further highlights ZNF787's impact on the gene regulatory network when its expression was increased due to miR-124 deletion or when overexpressed. However, ZNF787's overexpression did not hamper neurogenesis *per se* as the cells were still positive for MAP2 (Figure 6F).

In summary, our wTO analysis suggested that the TF networks were globally altered and differentially connected, especially at 3 dpi upon miR-124 depletion. In addition, our analysis identified uncharacterized TFs—of which *ZNF787* was experimentally validated—having regulatory functions during neurogenesis.

GO Term Analysis of wTO Network Nodes Reveals Indirect miR-124 Functions

We next investigated which biological functions were controlled by the TF networks of WT and ΔmiR-124 cells at each time point, especially which functions were common or different between the networks. To this end, we used our co-expression differential network analysis tool (Gysi et al., 2018) and classified each correlation into common or specific connections (Figures 7A–7C and S6B). Subsequently, we performed GO enrichment tests for these categories to reveal the biological processes the underlying genes were involved in.

GO groups that were common between the WT and ΔmiR-124 networks at 0 dpi included, for example, "mRNA processing," "cell division," and "mitotic cell cycle" (Figure 7A; Table S1). In particular, WT and ΔmiR-124 networks shared the groups "regulation of asymmetric cell division" and "regulation of extracellular matrix disassembly" at 1 dpi and "synapse assembly," "regulation of synapse organization," and "positive regulation of neurological system process" at 4 dpi, indicating that aspects of neurogenesis were also present in ΔmiR-124 cells.

However, we also found groups that were specific to the WT network. For example, the GO terms "layer formation in cerebral cortex" and "pyramidal neuron development" at 1 dpi, "regulation of exit from mitosis" and "positive regulation of long-term neuronal synaptic plasticity" at 2 dpi, "positive regulation of dendritic cell differentiation" at 3 dpi, and several "mRNA processing" groups at 4 dpi were different (Table S1). Many metabolic terms and cell signaling pathways (Wnt signaling at 3 dpi) were also specific to WT cells (Figure 7B). This indicates that particular functions related to cell-cycle control and neuronal differentiation started to be differentially controlled in WT and ΔmiR-124 cells right from the beginning of neurogenesis.

ΔmiR-124 groups differed in ion incorporation and diverse metabolic processes at 1 and 2 dpi and "signal transduction resulting in induction of apoptosis," "positive regulation of long-term neuronal synaptic plasticity," "regulation of dendritic spine morphogenesis," and "striatal medium spiny neuron differentiation" at 3 dpi (Figure 7C). In contrast to Wnt signaling in WT, we

Figure 6. Target-TF-Network Analysis Indicates Indirect—TF-Mediated—miR-124 Regulatory Actions

(A) Expression correlation as weighted topological overlap (wTO) between TFs that were differentially expressed at 3 dpi. Every panel shows the network at 3dpi for WT (middle), ΔmiR-124 (bottom), and the difference (top). The opacity of the lines indicates the wTO value of that link. Colored gene names represent a specific SOM cluster as shown in Figure 6B. Underlined TFs are filtered miR-124 targets (Figures 4B and 4C).

(B) Loess regression from self-organizing maps calculated on the basis of normalized fold changes of permanently (1 dpi–4 dpi) differentially expressed TFs. Color code represents the SOM clusters. Only four categories are shown (See also Figure S7D).

(C) Illustration of a miR-124 target-specific wTO subnetwork showing TF nodes at 3 dpi. Colored lines indicate negative or positive correlations of underlying associated genes.

(D) Illustration of the subnetwork shown in (C), including underlying associated genes.

(E) Quantification of *ZNF787* overexpression (OE) efficiency in WT neurons over time. $n = 3$ biological replicates. Significance was assessed with unpaired Student's t tests with Holm-Sidak correction for multiple comparisons with $^{***}p \leq 0.001$. Data are represented as mean ± SEM.

(F) Representative immunostainings for DAPI and the neuronal marker MAP2. Scale bar, 50 μm.

(G) GO term enrichment analysis of significantly downregulated transcripts ($p_{adj} < 0.05$, $\log_2$-fold change $< [-1]$) upon *ZNF787* overexpression indicating its impact on repressing neuronal differentiation and maturation.

(H) Heatmap of *ZNF787*-associated genes reappearing in target-wTO networks for WT, ΔmiR-124, and WT-ZNF787-OE (RNA-seq, z scores from rlog-transformed counts, $n = 3$ biological replicates). Arrows indicate similar expression trends for ΔmiR-124 versus WT and WT-ZNF787 overexpression versus WT.

See also Figures S6 and S7.

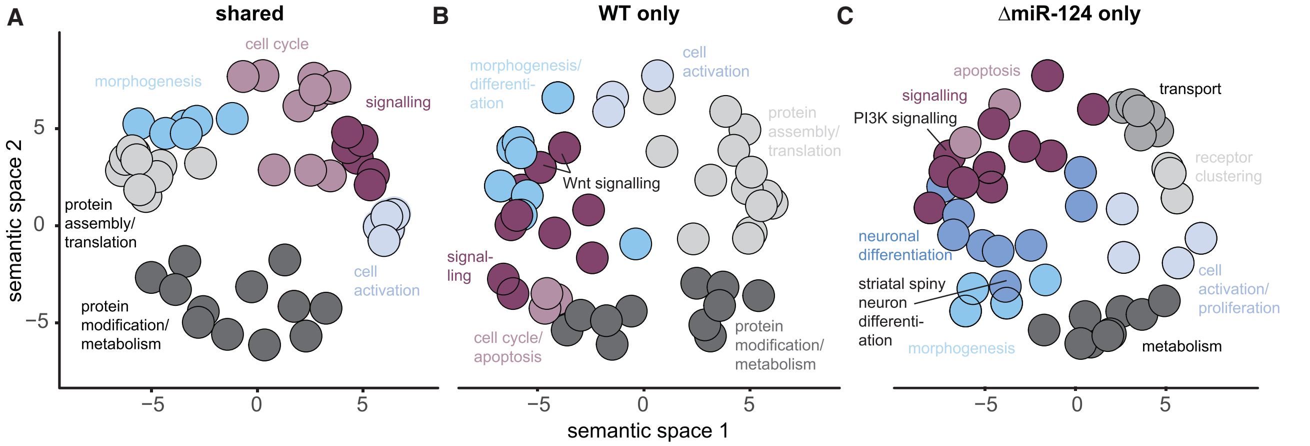

Figure 7. Semantic Clustering of GO Terms Reveals Shared and Different Biological Processes at 3 dpi in WT and ΔmiR-124 Cells
(A–C) Co-expression differential network analysis of shared (A), WT-specific (B), or ΔmiR-124-specific (C) regulated genes at 3 dpi. Underlying biological processes are grouped and highlighted. See also Figure S6.

detected phosphatidylinositol 3-kinase signaling in ΔmiR-124 groups. In particular, the GO term "striatal medium spiny neuron differentiation" was in line with the BrainSpan analysis (Figures 3C–3E). Our analysis further suggested that the differences in cell fate regulation were mediated by the altered miR-124-dysregulated TF networks in ΔmiR-124 cells at 3 dpi.

Our regulatory network analysis of the TFs, which are direct miR-124 targets, revealed clear differences in network architecture between WT and ΔmiR-124 samples: these increased until the cells become post-mitotic. These complex relationships, that is, indirect miR-124 functions, would have been impossible to detect by miR-124 target analysis alone. Moreover, biological functions underlying these regulatory network differences are in line with the phenotypic differences we observed when miR-124 was deleted.

DISCUSSION

Here, we present the first complete cellular human miR-124 knockout model. Previously, only a partial (one locus) miR-124 knockout mouse model had been studied, which showed small brain sizes, axonal mis-sprouting, and enhanced apoptosis (Sanuki et al., 2011), of which the latter is in line with our findings. However, by a partial knockout, compensatory effects driven by miR-124 paralogs could not be ruled out, and hence only very few of the potential miR-124 targets were identified. In light of previous studies, our findings are quite unexpected, as the initiation of neurogenesis appears to be miR-124-independent. We identified differences in metabolism and cell-fate regulation. For instance, we found that ΔmiR-124 neurons possess higher cholinergic neurotransmitter identity and are more similar to developing human striatum cells, which could be mechanistically explained through a co-expression differential network analysis. Hence, our systems-level analysis of regulatory miR-124-TF networks revealed that miR-124 impacts neuronal subtype differentiation.

Previous studies associated miR-124 with one or a few specific targets responsible for phenotypic changes. We experimentally verified 98 high-confidence targets, confirming that miR-124 acts at a "network-level" rather than controlling only one or a few genes. Still, the fact that we "only" verified 98 targets out of 2,338 genes with reduced 3′ UTR peaks in AGO2-IP samples is surprising and suggests that our approach may not be effective for filtering out all miR-124 targets. However, our data are completely in line with other studies using HITS-CLIP (Tan et al., 2013) and suggest that only a minority of targets bound by a unique miRNA display altered mRNA-levels upon miRNA-perturbation. Still, the list of miR-124 targets could increase since some targets are likely regulated only at the protein level, which could be interesting to study in the future using quantitative high-throughput proteomics, or because of regulatory compensation by other miRNAs or TFs. 38 of the identified miR-124 targets were already experimentally verified, and we further validated 43 using luciferase reporter assays, demonstrating the precision of our AGO2-RIP-seq approach for detecting miR-124 targets in parallel. We detected higher apoptosis rates in ΔmiR-124 neurons, which could be rescued by miR-124 overexpression. Hence, miR-124 regulation is essential for the long-term survival of terminally differentiated neurons by targeting directly but also indirectly a cascade of apoptosis-relevant genes. This provides context to previous studies that found miR-124 dysregulation in cases of Alzheimer disease (AD) or cognitive impairment (Fang et al., 2012; Gascon et al., 2014; Lukiw, 2007; Yang et al., 2014), suggesting that alterations in miR-124 may be linked to neurodegenerative disorders via control of apoptosis and neuronal survival, although these observations are controversial (De Strooper and Karran, 2016; Patrick et al., 2017). Our findings highlight the need for cell-type precision for linking certain miRNAs to cellular functions or pathologies. Sampling over many brain cell types might be misleading as neurons die in neurodegenerative diseases; therefore, reduced miR-124 levels are expected but not necessarily causally linked to primarily affected cell types. However, our data robustly support miR-124's neuroprotective function over time.

As 24 direct miR-124 targets were TFs, we were also interested in how the lack of miR-124 regulation affected their

networks. We achieved this by a wTO analysis of time course RNA-seq data. In contrast to previous miRNA-TF networks that were based on *in silico* predictions (Guo et al., 2010a), our target TF networks were computed from experimentally obtained RNA-seq data. Our recently developed wTO analysis allowed us to specifically account for both positive and negative correlations, which is crucial for analyzing regulatory networks that include transcriptional activators and repressors (Gysi et al., 2017; Nowick et al., 2009). Particularly, we found multiple yet uncharacterized zinc finger proteins such as *ZNF787* associated with miR-124 function. To start functionally characterizing *ZNF787*, we overexpressed *ZNF787* and found many biological processes for neuronal development and functions to be significantly reduced. We thus directly characterized this hereto uninvestigated ZNF as a neuronal feature repressor. Several other ZNFs targeted by miR-124 are human- or primate-specific (i.e., *ZNF138*, *ZNF37*, *ZNF701*, and *ZNF649*), suggesting that primate-specific regulators may have an impact on neuronal differentiation in humans. Besides impacts on coding transcripts, our AGO2-RIP-seq, sRNA-seq and nCounter data further enabled us to study miRNA species in ΔmiR-124 neurons that are normally very likely outcompeted by miR-124 for RISC incorporation in WT neurons. This dynamic change in miRNA regulation most likely masked and thereby reduced molecular outcomes in ΔmiR-124 cells and generally has to be taken into account when studying specific miRNA knockouts. Without studying indirect gene regulatory TF-network effects and dynamic miRNA profile changes, a sophisticated interpretation of miRNA manipulation data will be challenging and incomplete.

STAR★METHODS

Detailed methods are provided in the online version of this paper and include the following:

- KEY RESOURCES TABLE
- CONTACT FOR REAGENT AND RESOURCE SHARING
- EXPERIMENTAL MODEL AND SUBJECT DETAILS
  - Cell Lines
- METHOD DETAILS
  - Neurogenin-1/2-Induced Differentiation
  - iPSC Differentiation into mDA Neurons
  - Knockout of miR-124 Loci in Human iPSCs
  - Imaging and Image Analysis
  - miR-124 and ZNF787 Overexpression
  - Quantitative Real-Time PCR
  - SDS/Page Electrophoresis and Western Blot
  - Neurite Outgrowth Assay
  - Flow Cytometry
  - Apoptosis and Viability Assay
  - Luciferase Reporter Assays
  - Electrophysiological Measurements
  - Immunocytochemistry
  - RNA-Interacting Protein Immunoprecipitation
- QUANTIFICATION AND STATISTICAL ANALYSIS
  - Sequencing and Analysis of RIP Samples
  - RIP-Seq Binding Site Definition and Filtering
  - RNA-Seq from Total RNA, Processing and Data Analysis
  - Small RNA Quantification and Analysis
  - Neuronal Profiling Using BrainSpan Atlas
  - Self-Organizing Map Clustering
  - Weighted Topological Overlap (wTO)
  - GO Enrichment Analysis
  - Co-expression Differential Network Analysis (CoDiNA)
- DATA AND SOFTWARE AVAILABILITY

SUPPLEMENTAL INFORMATION

Supplemental Information includes 7 figures and 15 tables and can be found with this article online at https://doi.org/10.1016/j.cels.2018.08.011.

ACKNOWLEDGMENTS

We acknowledge technical support from the Center for Molecular and Cellular Bioengineering (CMCB) at TU Dresden Light Microscopy Facility, FACS Facility, iPSC Facility, and Sequencing Facility, specifically Andreas Dahl and Mathias Lesche. We thank Jared Sterneckert and Lydia Reinhardt for assistance with the small-molecule induction protocol and Frank Buchholz and Juliane Reh for sharing the nCounter equipment. The authors would also like to thank Eivind Almaas for the help and advise in developing CoDiNA and Alvaro Perdomo-Sabogal for sharing the manually curated general regulatory factors database. We thank Witold Filipowicz, Sara Oakeley, Alex H.M. Ng, and Jenna Hoersten for critical feedback; and the Busskamp, Nowick, and Jakobsson laboratories for support. L.K.K. was supported by the DIGS-BB program. D.M.G. was supported by a doctoral grant from the Brazilian government offices' Science without Borders program (GDE 204111/2014-5). J.F. was supported by a BMBF-funded project (FKZ 031A538A+B) within the German Network for Bioinformatics Infrastructure (de.NBI). J.J. was supported by the Swedish Research Council (# K2014-62X-22527-01-3), the Swedish Foundation for Strategic Research (# FFL12-0074), the Swedish Brain Foundation, and the Swedish Cancer Foundation. K.N. and P.F.S. were supported by the DFG within the SPP1738 on "Long Non-Coding RNAs in Primate Brain Evolution." The Volkswagen Foundation supported K.N. (initiative "Evolutionary Biology"). V.B. was supported by a Volkswagen Foundation Freigeist fellowship (A110720), by an ERC starting grant (678071-ProNeurons), and by the DFG Research Center (DFG FZT 111).

AUTHOR CONTRIBUTIONS

Conceptualization, L.K.K. and V.B.; Methodology, L.K.K., D.M.G., K.L., J.F., R.P., S.D.K., A.S., S.K., K.P., J.J., K.N., A.S., and V.B.; Software, L.K.K., D.M.G., K.L., J.F., and S.D.K.; Validation, L.K.K. and V.B.; Formal Analysis, L.K.K., D.M.G., K.L., J.F., S.D.K., K.P., and V.B.; Investigation, L.K.K., K.L., J.F., and V.B.; Resources, L.K.K., D.M.G., K.P., R.P., J.J., K.N., and V.B.; Data Curation, L.K.K., D.M.G., K.L., and J.F.; Writing – Original Draft, L.K.K. and V.B.; Writing – Review & Editing, all authors; Visualization, L.K.K., D.M.G., K.L., J.F., and S.D.K.; Supervision, P.F.S., J.J., K.N., and V.B.; Funding Acquisition, J.J., K.N., and V.B.

DECLARATION OF INTERESTS

The authors declare no competing interests.

Received: March 8, 2018
Revised: July 12, 2018
Accepted: August 23, 2018
Published: October 3, 2018

REFERENCES

Åkerblom, M., and Jakobsson, J. (2014). MicroRNAs as neuronal fate determinants. Neuroscientist *20*, 235–242.

Åkerblom, M., Sachdeva, R., Barde, I., Verp, S., Gentner, B., Trono, D., and Jakobsson, J. (2012). MicroRNA-124 is a subventricular zone neuronal fate determinant. J. Neurosci. *32*, 8879–8889.

Baek, D., Villén, J., Shin, C., Camargo, F.D., Gygi, S.P., and Bartel, D.P. (2008). The impact of microRNAs on protein output. Nature *455*, 64–71.

Benjamini, Y., and Hochberg, Y. (1995). Controlling the false discovery rate: a practical and powerful approach to multiple testing. J. R. Stat. Soc. Ser. B Methodol. *57*, 289–300.

Berto, S., Perdomo-Sabogal, A., Gerighausen, D., Qin, J., and Nowick, K. (2016). A consensus network of gene regulatory factors in the human frontal lobe. Front. Genet. *7*, 31.

Busskamp, V., Lewis, N.E., Guye, P., Ng, A.H., Shipman, S.L., Byrne, S.M., Sanjana, N.E., Murn, J., Li, Y., Li, S., et al. (2014a). Rapid neurogenesis through transcriptional activation in human stem cells. Mol. Syst. Biol. *10*, 760.

Busskamp, V., Krol, J., Nelidova, D., Daum, J., Szikra, T., Tsuda, B., Jüttner, J., Farrow, K., Scherf, B.G., Alvarez, C.P.P., et al. (2014b). MiRNAs 182 and 183 are necessary to maintain adult cone photoreceptor outer segments and visual function. Neuron *83*, 586–600.

Cao, X., Pfaff, S.L., and Gage, F.H. (2007). A functional study of miR-124 in the developing neural tube. Genes Dev. *21*, 531–536.

Carlson, M.R., Zhang, B., Fang, Z., Mischel, P.S., Horvath, S., and Nelson, S.F. (2006). Gene connectivity, function, and sequence conservation: predictions from modular yeast co-expression networks. BMC Genomics *7*, 40.

Chavez, A., Scheiman, J., Vora, S., Pruitt, B.W., Tuttle, M., Iyer, E.P.R., Lin, S., Kiani, S., Guzman, C.D., Wiegand, D.J., et al. (2015). Highly efficient Cas9-mediated transcriptional programming. Nat. Methods *12*, 326–328.

Cheng, L.C., Pastrana, E., Tavazoie, M., and Doetsch, F. (2009). miR-124 regulates adult neurogenesis in the subventricular zone stem cell niche. Nat. Neurosci. *12*, 399–408.

Chi, S.W., Hannon, G.J., and Darnell, R.B. (2012). An alternative mode of microRNA target recognition. Nat. Struct. Mol. Biol. *19*, 321–327.

Conaco, C., Otto, S., Han, J.J., and Mandel, G. (2006). Reciprocal actions of REST and a microRNA promote neuronal identity. Proc. Natl. Acad. Sci. USA *103*, 2422–2427.

Cong, L., Ran, F.A., Cox, D., Lin, S., Barretto, R., Habib, N., Hsu, P.D., Wu, X., Jiang, W., Marraffini, L.A., et al. (2013). Multiplex genome engineering using CRISPR/Cas systems. Science *339*, 819–823.

De Strooper, B., and Karran, E. (2016). The cellular phase of Alzheimer's disease. Cell *164*, 603–615.

Dutta, R., Chomyk, A.M., Chang, A., Ribaudo, M.V., Deckard, S.A., Doud, M.K., Edberg, D.D., Bai, B., Li, M., Baranzini, S.E., et al. (2013). Hippocampal demyelination and memory dysfunction are associated with increased levels of the neuronal microRNA miR-124 and reduced AMPA receptors. Ann. Neurol. *73*, 637–645.

Dweep, H., Sticht, C., Pandey, P., and Gretz, N. (2011). miRWalk–database: prediction of possible miRNA binding sites by "walking" the genes of three genomes. J. Biomed. Inform. *44*, 839–847.

Eichelser, C., Stückrath, I., Müller, V., Milde-Langosch, K., Wikman, H., Pantel, K., and Schwarzenbach, H. (2014). Increased serum levels of circulating exosomal microRNA-373 in receptor-negative breast cancer patients. Oncotarget *5*, 9650–9663.

Fang, M., Wang, J., Zhang, X., Geng, Y., Hu, Z., Rudd, J.A., Ling, S., Chen, W., and Han, S. (2012). The miR-124 regulates the expression of BACE1/β-secretase correlated with cell death in Alzheimer's disease. Toxicol. Lett. *209*, 94–105.

Franke, K., Otto, W., Johannes, S., Baumgart, J., Nitsch, R., and Schumacher, S. (2012). miR-124-regulated RhoG reduces neuronal process complexity via ELMO/Dock180/Rac1 and Cdc42 signalling. EMBO J. *31*, 2908–2921.

Gascon, E., Lynch, K., Ruan, H., Almeida, S., Verheyden, J.M., Seeley, W.W., Dickson, D.W., Petrucelli, L., Sun, D., Jiao, J., et al. (2014). Alterations in microRNA-124 and AMPA receptors contribute to social behavioral deficits in frontotemporal dementia. Nat. Med. *20*, 1444–1451.

Gibson, D.G., Young, L., Chuang, R.Y., Venter, J.C., Hutchison, C.A., and Smith, H.O. (2009). Enzymatic assembly of DNA molecules up to several hundred kilobases. Nat. Methods *6*, 343–345.

Grey, F., Tirabassi, R., Meyers, H., Wu, G., McWeeney, S., Hook, L., and Nelson, J.A. (2010). A viral microRNA down-regulates multiple cell cycle genes through mRNA 5′UTRs. PLoS Pathog. *6*, e1000967.

Gu, X., Meng, S., Liu, S., Jia, C., Fang, Y., Li, S., Fu, C., Song, Q., Lin, L., and Wang, X. (2014). miR-124 represses ROCK1 expression to promote neurite elongation through activation of the PI3K/Akt signal pathway. J. Mol. Neurosci. *52*, 156–165.

Guo, A.Y., Sun, J., Jia, P., and Zhao, Z. (2010a). A Novel microRNA and transcription factor mediated regulatory network in schizophrenia. BMC Syst. Biol. *4*, 10.

Guo, H., Ingolia, N.T., Weissman, J.S., and Bartel, D.P. (2010b). Mammalian microRNAs predominantly act to decrease target mRNA levels. Nature *466*, 835–840.

Gysi, D.M., Voigt, A., Fragoso, T., de, M., Almaas, E., and Nowick, K. (2017). wTO: an R package for computing weighted topological overlap and consensus networks with an integrated visualization tool. Arxiv E-Prints. https://arxiv.org/abs/1711.04702.

Gysi, D.M., Fragoso, T.M., Almaas, E., and Nowick, K. (2018). CoDiNA: an RPackage for Co-Expression Differential Network Analysis in n Dimensions. https://arxiv.org/abs/1802.00828.

Hafner, M., Renwick, N., Brown, M., Mihailović, A., Holoch, D., Lin, C., Pena, J.T.G., Nusbaum, J.D., Morozov, P., Ludwig, J., et al. (2011). RNA-ligase-dependent biases in miRNA representation in deep-sequenced small RNA cDNA libraries. RNA *17*, 1697–1712.

Hawrylycz, M.J., Lein, E.S., Guillozet-Bongaarts, A.L., Shen, E.H., Ng, L., Miller, J.A., van de Lagemaat, L.N., Smith, K.A., Ebbert, A., Riley, Z.L., et al. (2012). An anatomically comprehensive atlas of the adult human brain transcriptome. Nature *489*, 391–399.

Hill, C.G., Matyunina, L.V., Walker, D., Benigno, B.B., and McDonald, J.F. (2014). Transcriptional override: a regulatory network model of indirect responses to modulations in microRNA expression. BMC Syst. Biol. *8*, 36.

Hou, Q., Ruan, H., Gilbert, J., Wang, G., Ma, Q., Yao, W.D., and Man, H.Y. (2015). MicroRNA miR124 is required for the expression of homeostatic synaptic plasticity. Nat. Commun. *6*, 10045.

Hsu, P.D., Scott, D.A., Weinstein, J.A., Ran, F.A., Konermann, S., Agarwala, V., Li, Y., Fine, E.J., Wu, X., Shalem, O., et al. (2013). DNA targeting specificity of RNA-guided Cas9 nucleases. Nat. Biotechnol. *31*, 827–832.

Hsu, S.D., Lin, F.M., Wu, W.Y., Liang, C., Huang, W.C., Chan, W.L., Tsai, W.T., Chen, G.Z., Lee, C.J., Chiu, C.M., et al. (2011). miRTarBase: a database curates experimentally validated microRNA-target interactions. Nucleic Acids Res. *39*, D163–DD169.

John, B., Enright, A.J., Aravin, A., Tuschl, T., Sander, C., and Marks, D.S. (2004). Human microRNA targets. PLoS Biol. *2*, e363.

Kohonen, T., Hynninen, J., Kangas, J., and Laaksonen, J. (1996). SOM_PAK: The Self-Organizing Map Program Package. Technical Report A31, Helsinki University of Technology, Laboratory of Computer and Information Science (Finland: FIN-02150 Espoo).

Krichevsky, A.M., Sonntag, K.-C., Isacson, O., and Kosik, K.S. (2006). Specific microRNAs modulate embryonic stem cell–derived neurogenesis. Stem Cells *24*, 857–864.

Lagos-Quintana, M., Rauhut, R., Yalcin, A., Meyer, J., Lendeckel, W., and Tuschl, T. (2002). Identification of tissue-specific microRNAs from mouse. Curr. Biol. *12*, 735–739.

Lam, R.S., Töpfer, F.M., Wood, P.G., Busskamp, V., and Bamberg, E. (2017). Functional maturation of human stem cell-derived neurons in long-term cultures. PLoS One *12*, e0169506.

Landgraf, P., Rusu, M., Sheridan, R., Sewer, A., Iovino, N., Aravin, A., Pfeffer, S., Rice, A., Kamphorst, A.O., Landthaler, M., et al. (2007). A mammalian microRNA expression atlas based on small RNA library sequencing. Cell *129*, 1401–1414.

Lewis, B.P., Burge, C.B., and Bartel, D.P. (2005). Conserved seed pairing, often flanked by adenosines, indicates that thousands of human genes are microRNA targets. Cell *120*, 15–20.

Li, G., and Ling, S. (2017). MiR-124 promotes newborn olfactory bulb neuron dendritic morphogenesis and spine density. J. Mol. Neurosci. *6*, 159–168.

Lim, L.P., Lau, N.C., Garrett-Engele, P., Grimson, A., Schelter, J.M., Castle, J., Bartel, D.P., Linsley, P.S., and Johnson, J.M. (2005). Microarray analysis shows that some microRNAs downregulate large numbers of target mRNAs. Nature *433*, 769–773.

Linsen, S.E.V., de Wit, E., Janssens, G., Heater, S., Chapman, L., Parkin, R.K., Fritz, B., Wyman, S.K., de Bruijn, E., Voest, E.E., et al. (2009). Limitations and possibilities of small RNA digital gene expression profiling. Nat. Methods *6*, 474–476.

Liu, J., Carmell, M.A., Rivas, F.V., Marsden, C.G., Thomson, J.M., Song, J.J., Hammond, S.M., Joshua-Tor, L., and Hannon, G.J. (2004). Argonaute2 Is the catalytic engine of mammalian RNAi. Science *305*, 1437–1441.

Love, M.I., Huber, W., and Anders, S. (2014). Moderated estimation of fold change and dispersion for RNA-seq data with DESeq2. Genome Biol. *15*, 550.

Lukiw, W.J. (2007). Micro-rna speciation in fetal, adult and Alzheimer's disease hippocampus. NeuroReport *18*, 297–300.

Makeyev, E.V., Zhang, J., Carrasco, M.A., and Maniatis, T. (2007). The MicroRNA miR-124 promotes neuronal differentiation by triggering brain-specific alternative pre-mRNA splicing. Mol. Cell *27*, 435–448.

Mali, P., Yang, L., Esvelt, K.M., Aach, J., Guell, M., DiCarlo, J.E., Norville, J.E., and Church, G.M. (2013). RNA-guided human genome engineering via Cas9. Science *339*, 823–826.

Malmevik, J., Petri, R., Klussendorf, T., Knauff, P., Åkerblom, M., Johansson, J., Soneji, S., and Jakobsson, J. (2015). Identification of the miRNA targetome in hippocampal neurons using RIP-seq. Sci. Rep. *5*, 12609.

Martinez, N.J., and Gregory, R.I. (2013). Argonaute2 expression is post-transcriptionally coupled to microRNA abundance. RNA *19*, 605–612.

Miller, J.A., Ding, S.L., Sunkin, S.M., Smith, K.A., Ng, L., Szafer, A., Ebbert, A., Riley, Z.L., Royall, J.J., Aiona, K., et al. (2014). Transcriptional landscape of the prenatal human brain. Nature *508*, 199–206.

Miranda, K.C., Huynh, T., Tay, Y., Ang, Y.S., Tam, W.L., Thomson, A.M., Lim, B., and Rigoutsos, I. (2006). A pattern-based method for the identification of microRNA binding sites and their corresponding heteroduplexes. Cell *126*, 1203–1217.

Moore, M.J., Scheel, T.K.H., Luna, J.M., Park, C.Y., Fak, J.J., Nishiuchi, E., Rice, C.M., and Darnell, R.B. (2015). miRNA-target chimeras reveal miRNA 3′-end pairing as a major determinant of Argonaute target specificity. Nat. Commun. *6*, 8864.

Nowick, K., Gernat, T., Almaas, E., and Stubbs, L. (2009). Differences in human and chimpanzee gene expression patterns define an evolving network of transcription factors in brain. Proc. Natl. Acad. Sci. U S A *106*, 22358–22363.

Nowick, K., Fields, C., Gernat, T., Caetano-Anolles, D., Kholina, N., and Stubbs, L. (2011). Gain, loss and divergence in primate zinc-finger genes: a rich resource for evolution of gene regulatory differences between species. PLoS One *6*, e21553.

Patrick, E., Rajagopal, S., Wong, H.-K.A., McCabe, C., Xu, J., Tang, A., Imboywa, S.H., Schneider, J.A., Pochet, N., Krichevsky, A.M., et al. (2017). Dissecting the role of non-coding RNAs in the accumulation of amyloid and tau neuropathologies in Alzheimer's disease. Mol. Neurodegener. *12*, 51.

Petri, R., Pircs, K., Jönsson, M.E., Åkerblom, M., Brattås, P.L., Klussendorf, T., and Jakobsson, J. (2017). let-7 regulates radial migration of new-born neurons through positive regulation of autophagy. EMBO J. *36*, 1379–1391.

Rajasethupathy, P., Fiumara, F., Sheridan, R., Betel, D., Puthanveettil, S.V., Russo, J.J., Sander, C., Tuschl, T., and Kandel, E. (2009). Characterization of small RNAs in Aplysia reveals a role for miR-124 in constraining synaptic plasticity through CREB. Neuron *63*, 803–817.

Ran, F.A., Hsu, P.D., Wright, J., Agarwala, V., Scott, D.A., and Zhang, F. (2013). Genome engineering using the CRISPR-Cas9 system. Nat. Protoc. *8*, 2281–2308.

Ravasz, E., Somera, A.L., Mongru, D.A., Oltvai, Z.N., and Barabási, A.L. (2002). Hierarchical organization of modularity in metabolic networks. Science *297*, 1551–1555.

Reinhardt, P., Glatza, M., Hemmer, K., Tsytsyura, Y., Thiel, C.S., Höing, S., Moritz, S., Parga, J.A., Wagner, L., Bruder, J.M., et al. (2013). Derivation and expansion using only small molecules of human neural progenitors for neurodegenerative disease modeling. PLoS One *8*, e59252.

Sanuki, R., Onishi, A., Koike, C., Muramatsu, R., Watanabe, S., Muranishi, Y., Irie, S., Uneo, S., Koyasu, T., Matsui, R., et al. (2011). miR-124a is required for hippocampal axogenesis and retinal cone survival through Lhx2 suppression. Nat. Neurosci. *14*, 1125–1134.

Sedlyarov, V., Fallmann, J., Ebner, F., Huemer, J., Sneezum, L., Ivin, M., Kreiner, K., Tanzer, A., Vogl, C., Hofacker, I., et al. (2016). Tristetraprolin binding site atlas in the macrophage transcriptome reveals a switch for inflammation resolution. Mol. Syst. Biol. *12*, 868.

Shaw, M.W. (1973). Uses of banding techniques for the identification of human diseases of cytogenetic origin. Environ. Health Perspect. *6*, 151–156.

Silber, J., Lim, D.A., Petritsch, C., Persson, A.I., Maunakea, A.K., Yu, M., Vandenberg, S.R., Ginzinger, D.G., James, C.D., Costello, J.F., et al. (2008). miR-124 and miR-137 inhibit proliferation of glioblastoma multiforme cells and induce differentiation of brain tumor stem cells. BMC Med. *6*, 14.

Tan, C.L., Plotkin, J.L., Venø, M.T., von Schimmelmann, M., Feinberg, P., Mann, S., Handler, A., Kjems, J., Surmeier, D.J., O'Carroll, D., et al. (2013). MicroRNA-128 governs neuronal excitability and motor behavior in mice. Science *342*, 1254–1258.

Taniguchi, K., Sugito, N., Kumazaki, M., Shinohara, H., Yamada, N., Matsuhashi, N., Futamura, M., Ito, Y., Otsuki, Y., Yoshida, K., et al. (2015). Positive feedback of DDX6/c-Myc/PTB1 regulated by miR-124 contributes to maintenance of the Warburg effect in colon cancer cells. Biochim. Biophys. *1852*, 1971–1980.

Tian, G., Yin, X., Luo, H., Xu, X., Bolund, L., Zhang, X., Gan, S.Q., and Li, N. (2010). Sequencing bias: comparison of different protocols of microRNA library construction. BMC Biotechnol. *10*, 64.

Trapnell, C., Roberts, A., Goff, L., Pertea, G., Kim, D., Kelley, D.R., Pimentel, H., Salzberg, S.L., Rinn, J.L., and Pachter, L. (2012). Differential gene and transcript expression analysis of RNA-seq experiments with TopHat and Cufflinks. Nat. Protoc. *7*, 562–578.

Visvanathan, J., Lee, S., Lee, B., Lee, J.W., and Lee, S.K. (2007). The microRNA miR-124 antagonizes the anti-neural REST/SCP1 pathway during embryonic CNS development. Genes Dev. *21*, 744–749.

Walsh, J.G., Cullen, S.P., Sheridan, C., Lüthi, A.U., Gerner, C., and Martin, S.J. (2008). Executioner caspase-3 and caspase-7 are functionally distinct proteases. Proc. Natl. Acad. Sci. U S A *105*, 12815–12819.

Xue, Q., Yu, C., Wang, Y., Liu, L., Zhang, K., Fang, C., Liu, F., Bian, G., Song, B., Yang, A., et al. (2016). miR-9 and miR-124 synergistically affect regulation of dendritic branching via the AKT/GSK3β pathway by targeting Rap2a. Sci. Rep. *6*, 26781.

Yang, L., Zhang, R., Li, M., Wu, X., Wang, J., Huang, L., Shi, X., Li, Q., and Su, B. (2014). A functional miR-124 binding-site polymorphism in IQGAP1 affects human cognitive performance. PLoS One *9*, e107065.

Yaworsky, P.J., and Kappen, C. (1999). Heterogeneity of neural progenitor cells revealed by enhancers in the nestin gene. Dev. Biol. *205*, 309–321.

Yoo, A.S., Staahl, B.T., Chen, L., and Crabtree, G.R. (2009). MicroRNA-mediated switching of chromatin-remodelling complexes in neural development. Nature *460*, 642–646.

Yoo, A.S., Sun, A.X., Li, L., Shcheglovitov, A., Portmann, T., Li, Y., Lee-Messer, C., Dolmetsch, R.E., Tsien, R.W., and Crabtree, G.R. (2011). MicroRNA-mediated conversion of human fibroblasts to neurons. Nature *476*, 228–231.

Yu, J.Y., Chung, K.H., Deo, M., Thompson, R.C., and Turner, D.L. (2008). MicroRNA miR-124 regulates neurite outgrowth during neuronal differentiation. Exp Cell Res. *314*, 2618–2633.

Zhang, B., and Horvath, S. (2005). A general framework for weighted gene co-expression network analysis. Stat. Appl. Genet. Mol. Biol. *4*. Article17. https://doi.org/10.2202/1544-6115.1128.

STAR★METHODS

KEY RESOURCES TABLE

REAGENT or RESOURCE	SOURCE	IDENTIFIER
Antibodies		
chicken anti-MAP2	Abcam	ab92434
rabbit anti-hAGO2	Grey et al. (2010)	N/A
mouse anti-AGO2	Sigma-Aldrich	SAB4200085
Brilliant Violet 421-anti-NCAM	Biolegend	318328
PE-anti-TRA-1-60	BD	560193
Chemicals, Peptides, and Recombinant Proteins		
BDNF	Peprotech	450-02
GDNF	Peprotech	450-10
dbcAMP	Sigma-Aldrich	D0627
Doxycycline	Sigma-Aldrich	D9891
mTeSR hES medium (complete kit)	StemCell Technologies	05850
BrainPhys	StemCell Technologies	05790
NeuroCult SM1 Neuronal Supplement	StemCell Technologies	05711
N2 Supplement-A	ThermoFisher Scientific	17502048
L-Ascorbic Acid	Sigma-Aldrich	A0278
Matrigel hESC qualified	Corning	354277
ROCK inhibitor (InSolution Y-27632)	Merck Millipore	688001
TrypLE Express	ThermoFisher Scientific	12604013
P3 Primary Cell 4D Nucleofector Kit L/Pulse CB-156	Lonza	V4XP-3024
Critical Commercial Assays		
ApoToxGlo Assay	Promega	G6320
Dual Luciferase Assay	Promega	E1910
hsa-miR-124-3p miRCURY LNA miRNA PCR Assay	Exiqon/Qiagen	YP00206026
5S rRNA (hsa) miRCURY LNA miRNA PCR Assay	Exiqon/Qiagen	YP00203906
Universal cDNA Synthesis Kit II	Exiqon/Qiagen	203301
nCounter human miRNA panel v3	NanoString	CSO-MIR3-12
Deposited Data		
Raw and analyzed data	This paper	ncbi.nlm.nih.gov/geo/query/acc.cgi?acc=GSE118316
GRCh38.p10 (Genome Reference Consortium Human Build 38), INSDC Assembly	Ensembl	ensembl.org/info/data/ftp/index.html
Allen BrainSpan Atlas RNA-Seq	Miller et al. (2014)	brainspan.org
Uniprot database		Uniprot.org
miRTarBase	Hsu et al. (2011)	mirtarbase.mbc.nctu.edu.tw
miRWalk	Dweep et al. (2011)	http://zmf.umm.uni-heidelberg.de/apps/zmf/mirwalk2/
Experimental Models: Cell Lines		
hiPSCs/iNGN	Busskamp et al. (2014a); encodeproject.org; based on PGP1, Coriell	ENCBS369AAA GM23338 (primary fibroblasts GM23248)
293T/17 cells	ATCC	CRL-11268
Oligonucleotides		
sgRNA oligo miR-124-5’-1/2_1: CACCGATCAAGGTCCGCTGTGAACA	This paper	N/A
sgRNA oligo miR-124-5’-1/2_2: AAACTGTTCACAGCGGACCTTGATC	This paper	N/A

(*Continued on next page*)

Continued

REAGENT or RESOURCE	SOURCE	IDENTIFIER
sgRNA oligo miR-124-5'-3_1: CACCGTCTATACAATTAAGGCACG	This paper	N/A
sgRNA oligo miR-124-5'-3_2: AAACCGTGCCTTAATTGTATAGAC	This paper	N/A
sgRNA oligo miR-124-3'-1_1: CACCGCGCGGTGAATGCCAAGAATG	This paper	N/A
sgRNA oligo miR-124-3'-1_2: AAACCATTCTTGGCATTCACCGCGC	This paper	N/A
sgRNA oligo miR-124-3'-2/3_1: CACCGCACGCGGTGAATGCCAAGAG	This paper	N/A
sgRNA oligo miR-124-3'-2/3_2: AAACCTCTTGGCATTCACCGCGTGC	This paper	N/A
Recombinant DNA		
Plasmid: pSpCas9(BB)-2A-Puro (PX459) V2.0	Ran et al. (2013)	Addgene #62988
Plasmid: PB09-TRE-EF1a-beta-globin-miR-124-GFP-TetO-Puro	This paper	Will be provided via Addgene
Plasmid: PB09-TRE-EF1a-beta-globin-miR-ctrl-GFP-TetO-Puro	This paper	Will be provided via Addgene
Plasmid: pmiRGLO-SMAD4	Eichelser et al. (2014)	Addgene #78128
Plasmid: PB09-TRE-ZNF787-V5-Blasti	This paper	Will be provided via Addgene
Plasmid: pSMPUW-miR-GFP-Puro	Cell Biolabs	322001
Plasmid: PB09-TRE-ZNF787-V5-Blasti	This paper	Will be provided via Addgene
Software and Algorithms		
Segemehl	N/A	http://www.bioinf.uni-leipzig.de/Software/segemehl/
Samtools	N/A	http://samtools.sourceforge.net/
FASTQC	N/A	http://www.bioinformatics.babraham.ac.uk/projects/fastqc/
GSNAP	N/A	https://github.com/juliangehring/GMAP-GSNAP
featureCounts	N/A	http://bioinf.wehi.edu.au/featureCounts/
TopHat2	Trapnell et al. (2012)	https://github.com/infphilo/tophat2
rnacounter	N/A	https://github.com/jdelafon/rnacounter
Cuffdiff	Trapnell et al. (2012)	http://cole-trapnell-lab.github.io/cufflinks/cuffdiff/
DESeq2	Love et al. (2014)	http://bioconductor.org/packages/release/bioc/html/DESeq2.html
Weighted topological overlap (wTO)	Gysi et al. (2017)	https://CRAN.R-project.org/package=wTO
Package SOM	N/A	https://CRAN.R-project.org/package=som
CoDiNA	Gysi et al. (2018)	https://CRAN.R-project.org/package=CoDiNA
REVIGO	N/A	http://revigo.irb.hr

CONTACT FOR REAGENT AND RESOURCE SHARING

Further information and requests for resources and reagents should be directed to and will be fulfilled by the Lead Contact, Volker Busskamp (volker.busskamp@tu-dresden.de). All plasmids generated within this study are available at Addgene.org.

EXPERIMENTAL MODEL AND SUBJECT DETAILS

Cell Lines

iNGN cells (https://www.encodeproject.org, accession number: ENCBS369AAA) were previously described (Busskamp et al., 2014a). hiPSCs were cultured in mTeSR 1 media (05850, StemCell Technologies) on standard tissue culture plates coated with hESC-qualified Matrigel matrix (354277, Corning, incubated 45 min at room temperature) under standard conditions (5 % CO_2, 37°C). For passaging, hiPSCs were dissociated with TrypLE Express (12604013, Thermo Fisher Scientific), washed with DPBS

(pH 7.2; 14190169, Thermo Fisher Scientific), spun down (400 × g) and replated using mTESR with 3 μg/ml InSolution Y-27632 rho kinase inhibitor (688001, Merck Millipore) or frozen in mFreSR media (05854, StemCell Technologies).

CRTD5 hiPSCs were generated from BJ fibroblasts (CRL-2522, ATCC) at the CRTD iPS facility. Reprogramming was perfomed using the CytoTune-iPS 2.0 Sendai Reprogramming Kit (Thermo Fisher Scientific) according to the manufacturer's guidelines for transduction. Following transduction, the cells were cultured with ReproTeSR medium (05920, StemCell Technologies) on hES-qualified Matrigel for 18 to 21 days. Once stem cell colonies were sufficiently grown, the clonal colonies were mechanically isolated and expanded using mTeSR medium. Passaging was carried out similarly to iNGN cells. ReLeSR (05872, StemCell Technologies) was used for detachment in clumps. Pluripotency of the hiPS cells was tested using flow cytometry with Alexa Fluor 488-coupled anti-Oct3/4 (560253), PE-coupled anti-Sox2 (560291), V450-coupled SSEA-4 (561156) and Alexa Flour 647-coupled anti Tra-1-60 (560850, all BD Pharmingen) according to the manufacturer's protocol (Figure S2K). Furthermore, CRTD5 cells were tested positive for their differentiation capability for all three germ layers (data not shown). Karyotyping was performed as stated for iNGN WT/ΔmiR-124 cells (Figure S2L).

Stable integration of an inducible Neurog1/2 cassette to generate CRTD5-iNGN cells was performed using the PiggyBac transposon system. All promoter elements and open reading frames between the 5' core insulator and the SV40 polyA of the PiggyBac vector backbone PB-TRE-dCas9-VPR13 (Addgene plasmid #63800; Chavez et al., 2015) were removed using SpeI/SalI and replaced with PCR-amplified Neurog1/2 after a doxycycline-inducible promoter from the lentiviral iNGN construct (Addgene plasmid #61471; Busskamp et al., 2014a). Plasmids were transformed into chemically competent bacteria for amplification (One Shot Stbl3, Thermo Fisher Scientific). 10 μg of the plasmid were mixed with 2 μg of Super PiggyBac Transposase Expression Vector (PB210PA-1-S, Biocat) and electroporated to CRTD5 cells with the Lonza 4D X-unit, pulse CB-156 and the P3 Primary Cell 4D-Nucleofector Kit L (V4XP-3024, Lonza).

293T/17 cells (CRL-11268, ATCC) were cultured with DMEM + 10 % FBS (41966052, 10100147, Thermo Fisher Scientific) and passaged with trypsin-EDTA (25300054, Thermo Fisher Scientific).

METHOD DETAILS

Neurogenin-1/2-Induced Differentiation

0.5 μg/ml doxycycline (D9891, Sigma-Aldrich) was used to induce Neurog1 and -2 genes. After 4 dpi, half of the mTeSR 1 media was replaced with supplemented BrainPhys media (05790, StemCell Technologies), replaced daily until 7 dpi and further once per week. The following BrainPhys supplements were used for 10 ml media: 200 μl NeuroCult™ SM1 Neuronal Supplement (05711, Stemcell Technologies); 100 μl N2 Supplement-A (17502048, Thermo Fisher Scientific); 20 μl of 10 μg/ml recombinant human BDNF to a final concentration of 20 ng/ml (450-02, Peprotech); 20 μl of 10 μg/ml recombinant human GDNF to a final concentration of 20 ng/ml (450-10, Peprotech); 98 μl of 50 mg/ml dibutryl-cAMP to a final concentration of 1 mM (D0627, Sigma-Aldrich); 50 μl of 40 mM ascorbic acid to a final concentration of 200 nM (A0278, Sigma-Aldrich); and 100 μl of 100× penicillin-streptomycin (15140122, Thermo Fisher Scientific). After stable integration of the Neurog1/2 cassette to CRTD5 cells, the cells were differentiated similarly to iNGN cells.

iPSC Differentiation into mDA Neurons

Midbrain dopaminergic (mDA) neurons were generated via a small-molecule-based protocol (Reinhardt et al., 2013). For neural induction, confluent iPSCs were disaggregated using Accutase and subsequently resuspended as clumps in N2B27 medium (Neurobasal-A (10888022), DMEM-F12 (11330032), N2 (17502048), B27 (12587010), GlutaMAX (35050061), 15 mM HEPES (15630080); Thermo Fischer Scientific) supplemented with 1 μM dorsomorphin (3093, Tocris), 10 μM SB431542 (ab120163, Abcam), 5 μM ROCK inhibitor (Y-27632, 688001, Merck), 3 μM CHIR (CHIR-99021, Tocris), 200 μM ascorbic acid (AA, A0278, Sigma-Aldrich), and 0.5 mM purmorphamine (PMA, sc-202785, Santa Cruz Biotechnology) via embryoid body formation. Six days after neural induction, embryoid bodies were dissociated into smaller pieces using a 1000 μl pipette and plated on Matrigel (Corning)-coated 12-well plates. For expansion, smNPCs were cultured using N2B27 medium supplemented with 3 μM CHIR, 0.5 μM PMA, and 200 μM AA. To differentiate mDA neurons, smNPCs were patterned by plating smNPCs into N2B27 medium containing 0.5 mM PMA, 2 ng/ml human BDNF (450-02), 1 ng/ml GDNF (450-10, both Peprotech), and 200 mM AA. Maturation was induced by switching the medium to N2B27 medium containing 2 ng/ml BDNF, 2 ng/ml GDNF, AA, 1 ng/ml TGF-b3 (100-36E, Peprotech), 100 μM dibutryl-cAMP (D0627, Sigma-Aldrich), and 2 ng/ml activin A (120-14, Peprotech, first 2-3 days 5 ng/ml). For immunocytochemistry, differentiating mDA neurons were replated as single cells on day 14 of the differentiation onto coverslips and fixed on day 21. NEUN expression was quantified by counting positively stained nuclei using Fiji. Data were tested for significance using an unpaired Student's t-test. Data are presented as mean ± SEM.

Knockout of miR-124 Loci in Human iPSCs

sgRNA were designed by the MIT's CRISPR web tool (http://crispr.mit.edu/; Hsu et al., 2013). sgRNA sequences and corresponding oligos were used according to Table S2. All oligos were cloned into the vector pSpCas9(BB)-2A-Puro (PX459) V2.0 (a kind gift from Feng Zhang, Addgene plasmid #62988) as described in Ran et al. (2013). Four different sgRNA-expressing constructs were co-electroporated (10 μg per vector) into iNGN and CRTD5 cells by the Lonza 4D X-unit, pulse CB-156, and the P3 Primary Cell 4D-Nucleofector Kit L (V4XP-3024, Lonza), according to the manufacturer's guidelines. Cells were plated sparsely, and selected for PX459

integration after 24 h with puromycin. Grown colonies, derived from single cells, were picked and genotyped with primers specific for all miR-124 loci (Table S3) using the Kapa Hifi Mastermix (KR0370, Kapa Biosystems) or Takara PrimeStar Max Mastermix (R045B, Takara Bio) according to the manufacturer's protocol.

Potential off-targets (score $\leq$ 0.9 according to design tool; http://crispr.mit.edu/; Hsu et al., 2013) were tested by Sanger sequencing of corresponding PCR products that were amplified from genomic DNA using specific primers for the iNGN cell line (Table S4). 20 metaphase states from each cell line were karyotyped using G-banding (Shaw, 1973) via the iPS facility of the Center for Molecular and Cellular Bioengineering (CMCB) at the Institute of Human Genetics, University Clinics Jena, Germany, to test for chromosomal alterations.

Imaging and Image Analysis

Fluorescence imaging was performed on a Zeiss Axio Observer Z1 inverted fluorescence microscope. Images were processed using Fiji; the images were adjusted in the same way for every picture and channel from each experiment. Microscopic characterization and quantification of phenotypic differences were performed using an EVOS FL system (ThermoFisher Scientific) and manual counting using Fiji. Qualitative Brightfield images and images showing GFP expression of live cells were corrected for equal illumination throughout the well. Statistical comparison of morphological parameters was performed using an unpaired Student's t-test for two groups. Data are presented as mean ± SEM.

miR-124 and ZNF787 Overexpression

For miR-124 overexpression, a doxycycline-inducible promoter, the genomic miRNA-124 locus (±100 bp) placed within the human beta-globin intron (from pCMV-MIR vector, Origene) and a GFP-puromycin reporter were inserted into the SpeI/SalI linearized PiggyBac vector backbone PB-TRE-dCas9-VPR13 (Addgene plasmid #63800; Chavez et al., 2015) using isothermal assembly (Gibson et al., 2009) leading to the miR-124 expressing construct PB09-TRE-EF1a-beta-globin-miR-124-GFP-TetO-Puro. The control construct PB09-TRE-EF1a-beta-globin-miR-ctrl-GFP-TetO-Puro (EGFP-ctrl) was identical to PB09-TRE-EF1a-beta-globin-miR-124-GFP-TetO-Puro, but lacked the miR-124 hairpin, which resulted in a less bipolar ground state of the neurons due to the high GFP expression. The respective constructs were electroporated into iNGN cells and after two days selected for stable integration using 3 μg/ml puromycin. ZNF787 overexpression was performed using the same PiggyBac vector (PB-TRE-dCas9-VPR13) with addition of a doxycycline-inducible promoter, PCR-amplified ZNF787 (variant 1), a V5 tag and a blasticidin resistance gene after via isothermal assembly (construct PB09-TRE-ZNF787-V5-Blasti). Stable integration was assured by administration of 15 μg/ml blasticidin for at least three days.

Quantitative Real-Time PCR

For gene expression analysis, 250,000 cells of each corresponding sample group were seeded onto Matrigel-coated 12-well plates and induced with doxycycline. At 4 dpi, cells were detached with TrypLE Express (12604013, Thermo Fisher Scientific), spun down, and lysed with Qiazol (miRNeasy Mini Kit, 217004, Qiagen). The phases were separated with Phase Lock Gel tubes (heavy, 2 ml, 733-2478, 5PRIME) and the aqueous phase was cleaned-up with the miRNeasy Kit. 500 ng of the resulting RNA was reverse transcribed with the High-Capacity cDNA Reverse Transcription Kit (4368814, Thermo Fisher Scientific) or, for miRNA-specific analysis, the Universal cDNA Synthesis Kit with 5 ng input, respectively (203301, Exiqon). Primers for miR-124 were purchased from Exiqon/Qiagen (YP00206026). At least three biological replicates were used per sample and normalized to 5S RNA (YP00203906, Exiqon/Qiagen). For ZNF787, the expression analysis was conducted with ZNF787-specific primers and normalized to beta-Actin (ZNF787_for, CCAGTCACGAGAACCCAGTG, ZNF787_rev, CTCTGCGAGAAGGTCTTGCC; ACTB_for, CCTCGCCTTTGCCGATCC; ACTB_rev, CGCGGCGATATCATCATCC). Quantitative measurements were performed with the StepOnePlus Real-Time PCR System (Thermo Fisher Scientific) and Power SYBR Green PCR Master Mix (4367659, Thermo Fisher Scientific). Statistical comparison was based on the delta-delta-Ct method and performed using unpaired Student's t-tests and Holm-Sidak correction for multiple comparisons. Data are presented as mean ± SEM.

SDS/Page Electrophoresis and Western Blot

For sample preparation, cells were lysed in RIPA buffer (R0278, Sigma-Aldrich) mixed with protease inhibitor cocktail (1:25, 118735, Sigma-Aldrich) for 45 min on ice. Cells were then centrifuged at 10,000 × g for 10 min at 4°C. The supernatants were collected and transferred to a fresh tube. Samples were boiled at 95°C for 5 min in Laemmli buffer (1610737, BioRad). Proteins were separated on a 4–12 % SDS/PAGE gel. Gels were then transferred using the Transblot-Turbo Transfer system (BioRad). After transfer, membranes were blocked for 1 h in Tris-buffered saline (T5912, Sigma-Aldrich) with 0.1 % Tween20 (TBST, P1379, Sigma-Aldrich) and 5 % (wt/vol) non-fat dry milk (70166, Sigma-Aldrich) followed by overnight incubation at 4°C. The primary antibody was mouse anti-AGO2 (1:1,000; SAB4200085, Sigma-Aldrich). After two TBST washing steps of 15 min, membranes were incubated for 1 h at room temperature with HRP-conjugated secondary antibodies: anti-mouse (1:5,000; sc-2005, Santa Cruz Biotechnology). Actin staining was done using a monoclonal mouse anti-β-actin HRP (1:100,000; A3854, Sigma-Aldrich). Membranes were developed with the ECL Prime Western Blotting Detection Reagent (RPN2232, GE Healthcare). The signal was measured using a Chemidoc MP system (BioRad) and band intensities were quantified by densitometry using the ImageJ software.

Neurite Outgrowth Assay

iNGN cells (WT or ΔmiR-124) were seeded at a density of approximately 50,000 cells/cm^2 and induced with 0.5 μg/ml doxycycline. At 4 dpi, clusters of neurons were mechanically detached with a pipette tip und transferred to a PDL-laminin-coated 96-well plate for imaging (655090, Greiner). The next day pictures were taken every 10 min over a total time of 1 h with a Leica DMI6000 live-cell chamber. Neurite length was manually quantified from frame to frame using Fiji. Statistical comparison was performed using an unpaired Student's t-test for two groups. Data are presented as mean ± SEM.

Flow Cytometry

Cells were detached using TrypLE Express (12604013, Thermo Fisher Scientific), washed with DPBS without calcium and magnesium (14190169, Thermo Fisher Scientific), stained with PE-anti-TRA-1-60 (560193, BD) or the isotype control (555584, BD) and Brilliant Violet 421-anti-NCAM (318328, Biolegend) or the isotype control (400157, Biolegend). After staining for 1 h on ice, the cells were resuspended in FACS buffer comprising DPBS, 10 % fetal bovine serum (26140-079, Thermo Fisher Scientific), and 10 mM EDTA (15575-038, Thermo Fisher Scientific). A BD LSR II flow cytometer and BD FACSDiva software were used for analysis. Median fluorescence signal was used as measure for marker expression. Statistical comparison was performed using unpaired Student's t-tests and Holm-Sidak correction for multiple comparisons. Data are presented as mean ± SEM.

Apoptosis and Viability Assay

WT and ΔmiR-124 were seeded and differentiated consecutively according to the time frame from 0 to 14 dpi and all samples were measured with a SynergyNeo2 plate reader (BioTek) on the same day. The ApoToxGlo assay (G6320, Promega) was conducted according to the manufacturer's protocol. Statistical comparison was performed using unpaired Student's t-tests and Holm-Sidak correction for multiple comparisons. Data are presented as mean ± SEM.

Luciferase Reporter Assays

3' UTRs of potential miR-124 targets from the RIP-Seq Analysis were cloned into the pmiRGLO-SMAD4 vector (a gift from Heidi Schwarzenbach, Addgene plasmid #78128) (Eichelser et al., 2014) using NheI and XbaI, together with specific primers for the 3' UTR region (Table S5). A reversed miR-124 sponge sequence – not binding miR-124 – served as negative control for the assay. These constructs were transfected into four independent replicates of 293T/17 cells together with a miR-124 overexpressing construct (pSMPUW-miR-GFP-Puro with integrated miR-124 according to the manufacturer's protocol; or a control construct (Cell Biolabs) using polyethylimine 'Max' (24765-2, Polyscience). 48 h after transfection, the cells were harvested using the dual-luciferase assay kit (E1910, Promega). Luciferase activity (firefly and renilla) was monitored using a SynergyNeo2 (BioTek) (Table S6), normalized to the negative controls and tested for significance using unpaired Student's t-test and Holm-Sidak correction for multiple comparisons. Data are presented as mean ± SEM.

Electrophysiological Measurements

During the experiment, single cover slips with WT or ΔmiRNA-124 neurons were kept in filtered (0.2 μm) extracellular solution of the following composition (mM): NaCl 130 (S3014, Sigma-Aldrich), HEPES 20 (15630056, ThermoFisher Scientific), glucose 10 (CRTD media kitchen), KCl 5 (1049360250, Merck), $CaCl_2$ 2.5 (CRTD media kitchen), $MgCl_2$ (CRTD media kitchen), pH 7.3 with NaOH (1064950250, Merck). Neurons were localized under an upright microscope (Zeiss Examiner.A1 Axio) equipped with a water-immersion 20× objective (420957-9900, Zeiss). Neurons were selected for single-cell patch-clamp using whole-cell configuration. Microelectrodes had resistances of ~6 MΩ, were pulled from borosilicate glass (GB150EFT-8P, Science Products) with a P-1000 programmable horizontal puller (Sutter Instruments), and contained a filtered (0.2 μm) intracellular solution of the following composition (mM): KCl 130, HEPES 20, EGTA 10 (324626, Merck), $CaCl_2$ 0.25, pH 7.3 with KOH. The head stage was attached to a digitally controlled micro-manipulator (Luigs & Neumann SM-7) and connected to a multi-clamp 700 amplifier (Molecular Devices): analogue electrophysiological data were low-pass filtered at 6 kHz and subsequently converted to digital values at a sample rate of 20 kHz using a Digidata 1440A (Molecular Devices). Data was transferred to a PC using pClamp software (10.3, Molecular Devices) and analyzed using Clampfit software (10.3, Molecular Devices) and custom Python code with the neo package (neo.readthedocs.io). Pipette capacitance was always compensated for. For each neuron, several −10 mV pulses were applied after opening the cell to measure the capacitance in voltage-clamp mode at −60 mV and subsequently averaged. Capacitance was calculated as the integral of the capacitive current (carried charge) at offset of pulse, divided by the pulse amplitude. Cell capacitance was compensated for before further voltage clamp recordings. Peak and steady-state currents were analyzed by applying incremental depolarizing voltage pulses from −60 to 55 mV in 5 mV steps. Membrane potential was taken as the median of a 30 s recording in current-clamp mode (I = 0). Spiking behavior was assessed by injecting constant current to hold cells at around −70 mV. To find a neuron's maximum number of spikes, 500 ms increasing current steps were injected from −30 to 150 pA in 10 pA steps. Action potentials were detected using a threshold at −10 mV. All membrane voltages were corrected for a liquid junction potential, which was calculated to be 0.4 mV. No cover slip was measured for longer than 1 h.

Immunocytochemistry

Cells were grown on Matrigel-coated glass coverslips and fixed for 10–15 min with 4 % paraformaldehyde (15713, Electron Microscopy Sciences) in PBS (14040-117, ThermoFisher Scientific) and subsequently washed twice with PBS. Permeabilization

and blocking was performed using 10 % NDS (S30-100ML, Merck-Millipore), 0.5 % Triton X-100 (Sigma Aldrich), and 1 % BSA (15260037, ThermoFisher Scientific) in PBS for 20 min. Primary antibodies were applied overnight at 4°C in 3 % NDS in PBS. Subsequently, the samples were washed (10 min) three times with PBS, the secondary antibodies and DAPI (10236276001, Sigma-Aldrich) were applied for 1 h at room temperature in 3 % NDS in PBS. Three washes (10 min) with PBS-T (0.05 % Tween-20, P1379-250ML, Sigma Aldrich) were performed. Cells were mounted in Prolong Gold Antifade (P36934, Thermo Fisher Scientific).

Primary antibodies used in this study were mouse anti-NESTIN (1:300, MAB1259, R&D), goat anti-SOX1 (1:300, AF3369, R&D), rabbit anti-PAX6 (1:300, 901301, Biolegend), goat anti-SOX2 (1:300, 17320, Santa Cruz), mouse anti-FOXA2 (1:300, 101060, Santa Cruz), rabbit anti-TH (1:300, AB152, Merck Millipore), chicken anti-TUBBIII (1:700, AB9354, Merck Millipore), chicken anti-MAP2 (1:700, ab92434, Abcam), mouse anti-NEUN (1:700, MAB377, Merck Millipore), mouse anti-NCAM (1:700, 610921, BD), goat anti-DCX (1:700, sc-8066, Santa Cruz), rabbit anti-GFAP (1:700, ab16997, Abcam), and rabbit anti-dopamine (1:300, AB122S, Merck Millipore). Secondary antibodies were obtained from Thermo Fisher Scientific and were conjugated to AlexaFluor fluorochromes (1:1,000). Anti-rabbit-Cy3 (1:1,000, 711-165-152, Jackson labs) was also used.

RNA-Interacting Protein Immunoprecipitation

Cell pellets from approximately 50 million cells were homogenized, lysed in ice-cold lysis buffer (10 mM HEPES (pH 7.3, ThermoFisher Scientific), 100 mM KCl (1049360250, Merck), 0.5 % NP40 (NP40S, Sigma-Aldrich), 5 mM $MgCl_2$, 0.5 mM dithiothreitol (DTT, DTT-RO, Sigma-Aldrich), protease inhibitors (118735, Sigma-Aldrich), recombinant RNase inhibitors (10777019, ThermoFisher Schientific), 1 mM PMSF (36978, ThermoFisher Scientific) using TissueLyser LT (50 Hz, 2 min, Qiagen). For lysate clearing, homogenates were centrifuged for 15 min at 16,200 × g, 4°C. A 50 μl sample was taken as a pre-IP sample. The RIP samples were incubated with Dynabeads Protein G beads (10009D, ThermoFisher Scientific) coated with anti-hAGO2 antibody (a gift from JA Nelson) (Grey et al., 2010) for 24h at 4°C with end-over-end rotation. After incubation, beads were collected on a Dynamagnet (1 min, on ice, ThermoFisher Scientific) and gently resuspended in low-salt NT2 buffer (50 mM Tris-HCl (pH 7.5, T1503, Sigma-Aldrich)), 1 mM $MgCl_2$, 150 mM NaCl (S3014, Sigma-Aldrich), 0.5 % NP40, 0.5 mM dithiothreitol, 1 mM PMSF, protease inhibitors, recombinant RNase inhibitors). The beads were transferred to a new collection tube and washed with low salt NT2 buffer, followed by two washes with high salt NT2 buffer (50 mM Tris-HCl, pH 7.5), 1 mM $MgCl_2$, 600 mM NaCl, 0.5 % NP40, 0.5 mM DTT, protease inhibitors, 1 mM PMSF, recombinant RNase inhibitors). The solution containing the RNA was then transferred to Qiazol (Qiagen). Subsequently, RNA was isolated from RIP and pre-RIP samples according to the miRNeasy micro kit protocol (217084, Qiagen). The size distribution of the samples and the quality was checked with a Bioanalyzer RNA 6000 Pico Chip (5067-1513, Agilent Genomics).

QUANTIFICATION AND STATISTICAL ANALYSIS

Statistical details for each experiment are described in the Results and corresponding Methods sections as well as in the Figure legends.

Sequencing and Analysis of RIP Samples

cDNA libraries of three independent WT whole-cell RNA, ΔmiR-124 whole-cell RNA, WT-AGO2 RIP, and ΔmiR-124-AGO2 RIP samples for 0 and 4 dpi were prepared using NEBNext Ultra I Directional RNA Library Prep Kit for Illumina (E7420, NEB) with 900 ng input at the CMCB Sequencing Facility at TU Dresden (Table S7). PolyA selection, fragmentation, first strand and second strand cDNA synthesis, and purification were performed in combination with Agencourt AMPure Kit (A63880, Beckman Coulter) and repair/dA-tailing of the cDNA. Adapters were ligated to dA-tailed cDNA and size-selected using AMPure XP Beads (Beckman Coulter). RIP samples were not polyA-selected. Index primers (Illumina) were used to index library constructs, and a PCR was performed using NEBNext Q5 2X PCR Master Mix (M0543S, NEB). Libraries were purified using Agencourt AMPure Kit. Libraries were pooled and sequenced on a HiSeq 2500 (Illumina), resulting in ca. 33-56 million single-end 75 bp reads.

RIP-Seq reads were pre-processed (includes demultiplexing, barcode trimming, and adaptor removal) using BBDuk from the BBMap toolkit (github.com/BioInfoTools/BBMap, v36.14). After quality control with FASTQC (bioinformatics.babraham.ac.uk/projects/fastqc/, v0.11.4), reads were mapped to the human genome assembly hg38/GRCh38 using Segemehl (bioinf.uni-leipzig.de/Software/segemehl/, v0.2.0-418). Uniquely mapped reads were extracted for further analysis.

For differential expression analysis of the whole-cell RNA-Seq data reads were mapped to the human reference genome (hg38) using GSNAP (research-pub.gene.com/gmap/) with ENSEMBL annotation 81 and counted using featureCounts (http://bioinf.wehi.edu.au/featureCounts/; v1.5.2). Differential expression analysis was performed using DESeq2 (bioconductor.org/packages/DESeq2/; Love et al., 2014) on uniquely mapped reads.

RIP-Seq Binding Site Definition and Filtering

RIP-Seq identifies target transcripts/genes, sacrificing the high resolution of CLIP-Seq for the advantage of not performing a crosslinking procedure. Higher resolution and crosslinking techniques such as CLASH or CLEAR-CLIP, will most likely identify additional and lower abundant targets. However, a key aspect of our study is the combination of native AGO2-IP with expression data derived from RNA-Seq. This allows to normalize IP signal to target expression, adding a quantitative layer to our data, which was used to compare relative levels of miRNAs and miRNAs associated with the RISC complex between WT and ΔmiR-124 cells. The resulting list of potential miR-124 targets identified by RIP-Seq was intersected with a set of databases, which contain experimentally

validated targets as well as predicted targets (Tables S7, S8, and S9). These target predictions were conducted with seed regions, starting from a length of 6 nts. The combination of validated and predicted targets leads to a list of annotated miR-124 targets, rendering this initial set of targets most likely too large. Our AGO2-RIP-Seq data allowed us to identify bona fide miR-124 targets in iNGN cells, and relate our findings to well-established databases: the results confirm that we had identified biologically relevant targets. A binding site is defined as a region with a significantly higher number of read pile-ups than would be expected by chance at a given genomic position. We used a custom filtering method to split potential binding regions once certain height thresholds were reached. Cutoffs were defined based on signals detected in known miR-124 targets. Regions with a summit signal below 10 read pile-ups are considered background and were discarded. With a sliding window approach, starting from the summit, a binding site is first split when its height falls below 20 % of the summit signal. Emerging sub-sites with a summit above this cutoff and 10 pile-ups are then recursively split when their signal falls below 5 % of their summit, which also preserves regions with only weak signal. Replicates of each experimental setup were analyzed separately, and only peaks occurring in all replicates were considered for further analysis. Binding sites derived from uniquely mapped reads were annotated using the ENSEMBL Perl API for human annotation v90. Data were filtered using R and Excel (Microsoft) using Targetscan, miRanda, RNA22, and miRWalk (Dweep et al., 2011; John et al., 2004; Lewis et al., 2005; Miranda et al., 2006). Furthermore, miRTarBase for experimentally validated miRNA-mRNA interactions was used to extract validated targets of miR-124 (Hsu et al., 2011).

RNA-Seq from Total RNA, Processing and Data Analysis

Samples were prepared as for qRT-PCR analysis in seven biological replicates for sequencing of total RNA using the miRNeasy mini kit (Qiagen) and Phase Lock Gel tubes (heavy, 2 ml, 733-2478, 5PRIME) for enhanced phase separation according to the manufacturer's guidelines. cDNA libraries were prepared as RIP-Seq samples. Fasta files were processed and controlled for quality as RIP-Seq. Counts were computed using rnacounter (https://github.com/jdelafon/rnacounter) using RPKM and raw counts, from the v25 gencode annotation (Tables S12 and S13). Data comparing WT, ΔmiR-124 and ZNF787 overexpression were mapped and counted similarly to RIP-Seq whole-cell samples (Table S14). Differential expression (DE) and principal component analysis was calculated using DESeq2 with raw counts. The contrast used was ΔmiR-124 versus WT. Normalized fold changes (nFC) of the significant genes (Benjamini & Hochberg adjusted p-values < 0.05; Benjamini and Hochberg, 1995) were used to construct the timecourse analysis. Z-scores were used to visualize expression analysis per gene. For the ZNF787-associated genes, counts were rlog-transformed with DESeq2 prior to standardization.

Small RNA Quantification and Analysis

For small RNA sequencing (sRNA-Seq), cDNA libraries were prepared using NEXTFlex Small RNA Library Prep V3 (NOVA-5132-06, BIOO Scientific) with 50 ng input and 22 PCR cycles. Sequencing was performed using a HiSeq 2500 (Illumina). A similar approach as used for RIP-Seq analysis was also applied to sRNA-Seq data. After pre-processing, quality control, and mapping, read profiles were filtered using the same custom approach as for RIP-Seq data (Table S10). Due to the nature of sRNA-Seq data, multi-mapped reads were considered, but were neglected for the differential expression analysis. Regions that passed the filtering steps and were called in all replicates were again annotated using the ENSEMBL Perl API v90; peaks overlapping miRNA annotation were selected for downstream processing. Differential expression analysis was performed using DESeq2 on uniquely mapped reads as with RNA-Seq data. Complementarily, the nCounter human miRNA panel v3 (CSO-MIR3-12, NanoString) from 100 ng total RNA isolated, as stated for RNA-Seq, was used in combination with the Prep Station and the Digital Analyzer according to the manufacturer's guidelines. nCounter-miRNA counts were analyzed similarly to sRNA-Seq counts using DESeq2 (Table S11). For the computation of expression profiles, nCounter-derived raw counts were normalized to the corresponding counts of housekeeping genes on a per-replicate basis. The mean-normalized counts were used for data visualization. Of note, the nCounter miRNA Expression Assay kit did not include probes for all human miRNAs and not all miRNA probes were optimal (Busskamp et al., 2014a). For our analysis, we only focused on miRNAs that passed an nCounter threshold of >200 raw counts (corresponding to ∼0.004 normalized counts to housekeeping genes) at least in one of the 12 samples.

Neuronal Profiling Using BrainSpan Atlas

To align the iNGN read data to the human genome (hg38/hg19), TopHat2 v2.1.0 (Tuxedo suite; Trapnell et al. (2012); ccb.jhu.edu/software/tophat) was used. We determined the gene expression levels (FPKM) and the differentially expressed (DE) genes using Cuffdiff (http://cole-trapnell-lab.github.io/cufflinks/cuffdiff/, v2.2.1; Gencode v26/v10). To identify the genes related to brain development, we calculated the 1,000 highest DE genes of the WT and ΔmiR-124 cells at 4, 7 and 14 dpi with respect to 0 dpi, respectively. Subsequently, the Pearson correlation coefficient was derived for the remaining FPKMs of each BrainSpan sample and our cell lines at 4, 7 and 14 dpi. The BrainSpan Atlas by the Allen Institute (www.brainspan.org; Miller et al., 2014) contains RNA-Seq data from 16 cortical and subcortical brain regions from both female and male human individuals. The samples span from 8 postconceptional weeks to 40 years (29 timepoints in total). In order to test whether a brain region had a higher correlation to any given age, we determined if the differences in means was larger than twice the standard deviation of samples with a similar age. For all analyses, we combined cerebellum and cerebellar cortex data from the BrainSpan Atlas since their timepoints did not overlap and calculated the mean of the FPKMs between individuals for each timepoint and region. Graphical brain illustrations were performed as previously described (Hawrylycz et al., 2012).

Self-Organizing Map Clustering

TFs that were differentially expressed at all timepoints from 1 to 4 dpi were clustered according to their nFC pattern over time using the Self Organizing Maps (SOM) algorithm (Kohonen et al., 1996), implemented in R using the package som (https://CRAN.R-project.org/package=som). The number of clusters was increased until the q-error of each group was reduced, with the average distortion measure under 10. The membership of the genes to each SOM cluster was used to color the genes in the (target-) TF-TF network analysis.

Weighted Topological Overlap (wTO)

The wTO is a measure of how a set of genes of interest is correlated. Having a high absolute wTO means that the expression of both genes is highly (positively or negatively) correlated. The wTO computes not only the signed weight of this relationship, but also the probability of the relationship being random. The R package wTO (https://cran.r-project.org/web/packages/wTO/index.html; Gysi et al., 2017) was used to calculate the wTO of the (target-) TF-TF networks (Table S15). The correlation between a set of genes was corrected using all the other genes present, thus reducing the noise and the false positives, and taking into account the commonalities of those genes. The parameters used in this calculation were Pearson product-moment correlation coefficient and 1000 bootstraps resampling. The final results were filtered to a probability of 0.10 for having random wTO. The wTO was calculated based on RPKM values. Genes with RPKM < 5 for each day were removed. From the total of 56269 mapped transcripts from the RNA-Seq dataset, 39,275 are considered to be expressed, using the criteria previously described. Only TFs from the RIP-Seq high probability target list (assembled from the Gene Regulatory Factor (GRF) Catalog (Berto et al., 2016) were considered for the network analysis: these are associated with gene ontology terms for regulation of transcription, DNA-dependent transcription, RNA polymerase II transcription co-factor and co-repressor activity, chromatin binding, modification, remodeling, or silencing, among others. The wTO R package was used to visualize the interactive plots. igraph (igraph.org/r/) and network (CRAN.R-project.org/package=network) R packages were used to visualize the developmental network. Human or primate specificity was judged according to the Uniprot database (uniprot.org).

GO Enrichment Analysis

GO enrichment analysis was conducted using the R package topGO (bioconductor.org/packages/topGO) using all expressed genes (TPM > 0.01 or average RPKM > 10) as background for each day. The GO enrichment analysis for days 7 and 14 was done separately for up- and down-regulated genes, according to the DE analysis. Semantic clustering was performed with REVIGO (revigo.irb.hr) using the SimRel measure and allowed similarity of 0.9).

Co-expression Differential Network Analysis (CoDiNA)

Identifying similarities and differences between the WT and ΔmiR-124 wTO-TF networks was performed using the CoDiNA method implemented in the R package CoDiNA (Table S1) (Gysi et al., 2018), a recently-developed method to classify links and nodes according to Φ categories, to its commonalities, differences and specificities. Links are considered to be common (α) if they belong to a set of networks (WT and ΔmiR-124 for each day) with the same sign and similar strength. If the sign changes from one network to another, the link is considered different (β). If a particular link belongs to one network only, it is considered to be specific to this network (γ). α and β categories are intersections of correlated genes between WT and ΔmiR-124: γ links are exclusive for one condition. The classification of the interactions according to these concepts is central to understand how the ΔmiR-124 networks are affected in their TF interactions during the time course. The distance of each link to the origin is calculated in order to score links that are more common, different or specific. Links with a normalized Φ distance greater than 0.5 were kept for further analysis.

In order to define the category a TF belongs to, a χ^2 goodness-of-fit test was used to test if the distribution of the links is different than 1/3 for each category ($p < 0.05$). Each TF is classified using the link category that appears most frequently for that particular TF. The correlation between TFs and genes was measured using a Pearson product-moment correlation coefficient. Only absolute correlations > 0.9 were considered to be correlated for the following analyses. CoDiNA was computed separately for each day. In order to compare WT and ΔmiR-124 networks, the TFs were distinguished according to category; the names of the genes correlated with the TF were retrieved. The GO enrichment analysis was performed as stated above.

DATA AND SOFTWARE AVAILABILITY

The RNA-Seq data discussed in this publication have been deposited in NCBI's Gene Expression Omnibus and are accessible through the GEO SuperSeries accession number GSE118316 (https://www.ncbi.nlm.nih.gov/geo/query/acc.cgi?acc=GSE118316). The wTO tool is available at https://CRAN.R-project.org/package=wTO and CoDiNA under https://CRAN.R-project.org/package=CoDiNA. Raw counts for RNA-Seq data are also provided in Tables S7, S10, S12, S13, and S14. AGO2 binding signal at the 3' UTR is provided in Table S8. nCounter raw data are listed in Table S11. The high-probability miR-124 target list is provided as Table S9. Luciferase assay raw data are listed in Table S6.

Cell Systems, Volume 7

Supplemental Information

Combined Experimental and System-Level Analyses

Reveal the Complex Regulatory Network

# of miR-124 during Human Neurogenesis

Lisa K. Kutsche, Deisy M. Gysi, Joerg Fallmann, Kerstin Lenk, Rebecca Petri, Anka Swiersy, Simon D. Klapper, Karolina Pircs, Shahryar Khattak, Peter F. Stadler, Johan Jakobsson, Katja Nowick, and Volker Busskamp

Supplemental Information

Supplemental Tables S2 – S5 (Tables S1, S6-15 are provided as separate Excel files).

Supplemental Figures S1 – S7 including legends.

Table S2. Oligos for sgRNA cloning. Related to Figure 1.

sgRNA	Sequence (PAM)	Oligos
**miR-124-5'-1/2**	ATCAAGGTCCGCTGTGAACA (CGG)	CACCGATCAAGGTCCGCTGTGAACA, AAACTGTTCACAGCGGACCTTGATC
**miR-124-5'-3**	GTCTATACAATTAAGGCACG (CGG)	CACCGTCTATACAATTAAGGCACG, AAACCGTGCCTTAATTGTATAGAC
**miR-124-3'-1**	CGCGGTGAATGCCAAGAATG (GGG)	CACCGCGCGGTGAATGCCAAGAATG, AAACCATTCTTGGCATTCACCGCGC
**miR-124-3'-2/3**	CACGCGGTGAATGCCAAGAG (AGG)	CACCGCACGCGGTGAATGCCAAGAG, AAACCTCTTGGCATTCACCGCGTGC

Table S3. Genotyping primers for miR-124 expressing loci. Related to Figure 1.

Primer/Locus	Sequence
**miR-124-1_For**	TTATCTCATTGTCTGTGTGATTGGG
**miR-124-1_Rev**	CCCTGAGTCTGTTTGCATCTCTAAG
**miR-124-2_For**	AGGCTTATGTATGTTTTTAGGCGTG
**miR-124-2_Rev**	AAGAGATGGGTTCCTTTTACTACCC
**miR-124-3_For**	GAAAGGGGAGAAGTGTGGGCTCCTC
**miR-124-3_Rev**	GCCATTTCCATGAGAAAGGAGCGG

Table S4. Off-target primers to detect top predicted off-targets. Related to Figure S1 and STAR Methods.

Gene	Alternative sgRNA (PAM)	Primers used
**SNAPC4, NM_003086**	GACCAGGTCCGCAGTGAACA (GAG)	For: CTTGGTTCCTGTTTGTCTGTCCC<br>Rev: TGTCCAGCTCCACAATTAAAAACA

**THRA, NM_199334**	ATCTAGAACCACTGTGAACA (GAG)	For: GGATATTCGAGTAACCACCGTCA<br>Rev: AATTTCCGCCTCTGTTTCCAATG
**ASB12, NM_130388**	ATCACGGGCAGCTGTGAGCA (CGG)	For: AAACGTTTCATCAACAGCAGGAG<br>Rev: GAAGCTATGTTTGATGCCCAGAC
**C1orf87, NM_152377**	GTCTATAGCAATAAGGCACA (GGG)	For: CTTGGGTGGATAACCTTTTCATAGC<br>Rev: TCCTTTTCTGCCTTGTTTTTCACTT
**PARD6B, NM_032521**	GTCTTTACAAATACGGCAC G(GAG)	For: TATTGCGTCCTGACAACCATAGAAA<br>Rev: TCTCACTGTTATGATGAGGTTACGG
**PCYOX1L, NM_024028**	GGCCGTGGCTGCCAAGAATG (TGG)	For: CTAAAGACCCTGTTCCGTTCCTATT<br>Rev: TGATACATGAGGGGTCAGAAAGAAG
**GIPR, NM_000164**	ACAGGAGAATGCCAAGAATG (CGG)	For: ATTTGGTGGATTATACGGACCCC<br>Rev: TGGAAGGAGCTGAGGAAGATCTCAA
**ZAN, NM_173059**	CAGGGTGACAGCCAAGAATG (AGG)	For: CAGTGGTAGTGGTTAGGGAGCA<br>Rev: GTGGAAAGGTTCAGGGATTGGAAGG
**ZNF540, NM_001172226**	CCCTATGAATGTCAAGAATG (TGG)	For: TCAACTGATAGAAAACGTCCCTCTT<br>Rev: CCCTTATCACATTTCTTACACATATAGGG
**ZNF331, NM_001253800**	CCCTATGAATGTCAAGAATG (TGG)	For: GGAAGACCTTTAGCCGTGTGT<br>Rev: TCTGTGCACTTGTACGGCTT
**CLN3, NM_001042432**	CAGCAGGTGAAGGCCAAGAG (GAG)	For: CTCTGCGGTCTCACTCTATTCTC<br>Rev: GACAGAATGAATCCCTTTCCTCTGG
**SNX20, NM_153337**	TACGGGTTGAAAGCCAAGAG (TGG)	For: ATAAGCATTAGAGACCTGCAATCAC<br>Rev: GGGAGGGCATTTTCTTGATCTTAC
**CCDC62, NM_201435**	CTTGCGATGAATGCAAAGAG (AAG)	For: CACCCAAAAGTCGATATTAAGAGGG<br>Rev: TGACATTTTGGTGATTTTGTGTGGA

Table S5. Luciferase assay primers for 3‘ UTR amplification. Isoforms are discriminated according to Ensembl. Related to Figure 4.

Gene	Primers used
**CTDSP1-201**	For: TGAGGGTGATGGGGCCAG<br>Rev: TAGGGGTCTGGCTTTCTCTTTAGTT
**CD164-201**	For: ACAGACCCATTGAATTAATAAGGACTG<br>Rev: TTCAATAAACATGTAGATTTATTTTAAGTCAGTTTG
**CERS2-201**	For: ACCATTACTCCAGCTGCCTCC<br>Rev: TGCAAGGAAGGCATAAGAAAGACTC
**GLIS2-201**	For: GCCCATCCTGCGGACAGTTGTGGTG<br>Rev: CATCTTTGCAACTCACTTTTATTGT
**TP73-211**	For: AGATCCCCGAGCAGTACCGCATGAC<br>Rev: TCATTGGAACATACACTTTATTATG
**BAX-201**	For: TCAATCCCCGATTCATCTACCCTGC<br>Rev: TGTGTCCCGAAGGAGGTTTATTACC
**PPM1F-201**	For: GTGGTTTCCAGGCCCCTGCCCTCCC<br>Rev: CAGAGTTCAGAACTTGTGGTTTATT
**CDIP1-201**	For: CGGAGCTGGGACTCGGGACTCCCCC<br>Rev: TTTAAACAATTCATTCTCTAAAAGA
**ADGRL1-201**	For: GGGCACCTCATGGACCAGGGG<br>Rev: TCTTAATTTCTTTATTGTTTGACTTTTTGAC
**ARF1-214**	For: ACGCGACCCCCCTCCCTCT<br>Rev: GAAATAGTTAAGAGATTTATTCTAATAGCTATAA
**ARHGAP4-203**	For: GGTGCCGCTGCTGGAGATGC<br>Rev: GGTGCCGCTGCTGGAGATGC

**ASIC1**	For: GCCCCGCAGGCCGCT<br>Rev: TGGTTGAAACCCAGTTTATTAGACCAGAGGCATG
**ATF5-201**	For: AAGGGCAGGGGTGTGGCTTCTG<br>Rev: CCAGAGGAAATGTTTCGTTTTATTTTTGCTCAT
**CALM1-201**	For: AGACCTACTTTCAACTCCTTTTTCCCC<br>Rev: TGAAAGGCTGCAGAAATGTTTATTGAATACAGTG
**CAMK2B-209**	For: AGCTGCGCCCTGGTTTCG<br>Rev: TGTCCTGTTTTTCTTTTATTTGCAGTTTCCCGAG
**CRTAP-201**	For: CCCACAGCAACCAAAGAGACTTC<br>Rev: TAAGCAATGTAAAACATTTAATGTGATTTAAAAAG
**FAM102A-203**	For: GGAGCAGGTGTCCGGGCTG<br>Rev: AAAGAAACAAAAACCGAAACATTTAATTTTTTCTCCCC
**FAM104A-202**	For: TGTGCCGGCAGTTTCTTGCCTTC<br>Rev: TGAATGACAAAATGCACTAATGCTGCTTTTAATG
**FKBP1A-204**	For: ATCTGCCATGGAGGGATCTGGTG<br>Rev: TGAGAAAAGCCGGCATAAAGCACTTTTATTG
**GRM4-202**	For: AGTCCATGGAGCTGAGCAGCAG<br>Rev: ATGGTGACATGCTTTAATTATCCACCGAG
**KIAA2013-201**	For: GTGAACTAACAGTCCTGCTTT<br>Rev: TGAAAGTTTGAAAGATATTTATTTAAAAACAGAA
**LEMD2-201**	For: GCCCCGGGCGGGGAC<br>Rev: GGCAAGAAACATCCGTTTTTAATAAATAGTTTATTC
**LFNG-201_UTR**	For: TGGCCATGGCTGAGACCCAA<br>Rev: TTGCTGCAAAGAGCACCTTTATTCACAG
**LFNG-203**	For: GGCACGGAGCAGCTGATGCC<br>Rev: TTTGAGGAAGGAAAAGCACCGCAGAGC
**MAFG-201**	For: GGACGCGCGTCTGC<br>Rev: CTCTTTAAAAGGTTTATTGATCATATACA
**MTMR10-201**	For: AATAGGGTGTTTTCTGAACATTTTGAG<br>Rev: AACAGTCGCTGTGGAATTTTATTAAG
**PDK2-203**	For: GGGCCGCCGTGCATCTG<br>Rev: TGGTCTTTGACCCATTCTCCAAGC
**PLEKHG5-212**	For: GCAGAGGGAGGCCCCCAAGAG<br>Rev: GCGGCCGCTGCTCCATAGCC
**POLR2E-213**	For: CCCCTAGAGGCGGACACACAG<br>Rev: TTCTTTCACAGGCACGTTTATTTTGCTGAAA
**PTPN18-201**	For: GTCTAACGCCAGTTCCTGCCTG<br>Rev: TGAATCGGAGAAATGGACAGTTTATTACTCACAG
**RASSF4-201**	For: CTGGCCAACACCTGCCTCTTCC<br>Rev: TTTGCTTTATCTTCGGATACGTTTATTACCTGAG
**RGS3-201**	For: GCAAACCCAAGGGCGGGAC<br>Rev: CCCTCTTAAAGAAATCTGTGTTTTTTCTTTC
**RPAIN-202**	For: AGCCAGCTTGGACTCACATC<br>Rev: GTTTTGTGTTCTCATTTATTATCATTTTTTTCTG
**RPLP1-201**	For: ACCTCTTTTATAACATGTTCAATAAAAAGC<br>Rev: GAACACACAATACCCACGGTTTATTATATAAATC
**SEMA5B-202**	For: TACCGCCGTCCTGGGGACT<br>Rev: GAGACTGGATATCATCTTTAATTAATAATGC
**SEMA5B-211**	For: TACCGCCGTCCTGGGGACT<br>Rev: GGAGACTGGATATCATCTTTAATTAATAATGC
**SERINC2-202**	For: GGCAGCCTCACAGCCTGC<br>Rev: GGTACACGCACTGGCTTGTTTATTAG
**SH3GLB2-203**	For: GCAGGTGCCCCCATCCC<br>Rev: TTTGCAGTTTTGGTAATTCTGTGGTCT
**SPRED2-201**	For: CTCAGTTTCCCTCCCTTCTCCC<br>Rev: TCTGATAGGATGTGTTTATATTTACACGGTAC
**SRM-201**	For: GCCCAGGCGCCACCACTG<br>Rev: TTTGCTATAAATACACGTGTTTGGTGAGTG

**STK11-201**	For: CCCGTGTCCAGGAGCCCC<br>Rev: CAGGTCCAAGCTTCCCAAGCTT
**SUMF1-201**	For: CAACCAAGGAAAGTCTTCCCCAG<br>Rev: CCTGATTTAGCAGATACGTTTATTCAACACA
**TACC3-201_UTR**	For: ACCTCCACGGAGCCGCTGT<br>Rev: TTTAAATTGAAAGGAAACTTTTATTGAGTC
**TMEM134-201**	For: TCGCGGCGCAGCGTGGACC<br>Rev: TGTGGCTATATCAGAAATTTTAATAAATAACAGTA
**TMEM63C-201**	For: CCGGGACCTGAGGCCTCCA<br>Rev: GAGGTTGGGAGGACTTGTTTATTCAAGG
**TNFRSF10B**	For: GTGTGATTCTCTTCAGGAAGTCAGAC<br>Rev: TTTTTATATAAGGTTTCATATTTAATTTGGTCATGG
**TRIT1-201**	For: GAGACATGTCCAGTGGCCTTTG<br>Rev: TCTTGTAAATTTTTTCTTTATTTAAACTTCAATAAAAATATAG
**TSPAN15-201**	For: GGCCCAGCCTGCCATGGCA<br>Rev: AAAAAAAAAAAAAAAAAACATGTTTTTATTGTTTGATTA
**UBE2G2-202**	For: GACCTGGCCTCGCACAGGC<br>Rev: TCACTGACACAGGAATCTGCCTCTTTATTGAC
**UBE2I-202**	For: GCAGCGACCTTGTGGCATCGT<br>Rev: GTCTGTGCAAATGCTTTAATGGTGG
**ZNF787-203**	For: GGGAGGGGCCCGGGGGGGGGG<br>Rev: GACATTTGGATAGTGCCGTTTATTG

A

iNGN **WT**, Karyotype: 46, XY [cp20] iNGN **ΔmiR-124**, Karyotype: 46, XY [cp20]

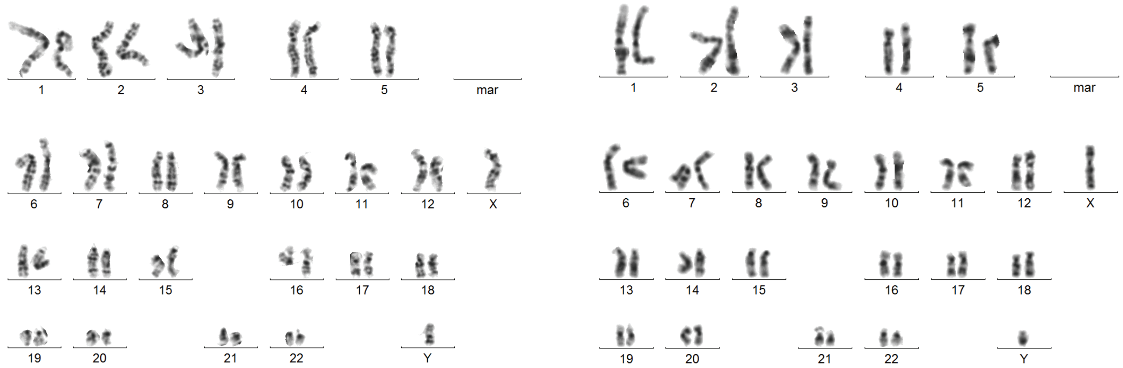

B

Gene	Score	Alternative sgRNA (PAM)	Sequence from Sanger sequencing
**SNAPC4, NM_003086**	0.6	GACCAGGTCCGCAGTGAACA (GAG)	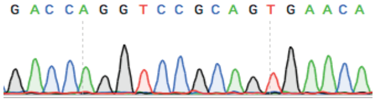
**THRA, NM_199334**	0.5	ATCTAGAACCACTGTGAACA (GAG)	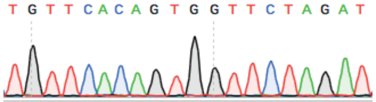
**ASB12, NM_130388**	0.3	ATCACGGGCAGCTGTGAGCA (CGG)	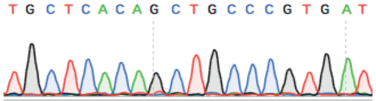
**C1orf87, NM_152377**	0.2	GTCTATAGCAATAAGGCACA (GGG)	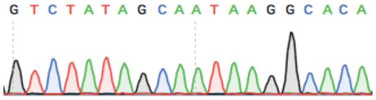
**PARD6B, NM_032521**	0.2	GTCTTTACAAATACGGCAC G (GAG)	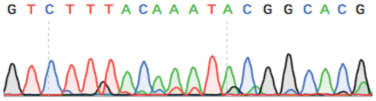
**PCYOX1L, NM_024028**	0.9	GGCCGTGGCTGCCAAGAATG (TGG)	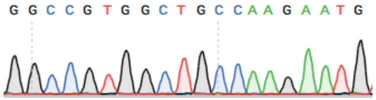
**GIPR, NM_000164**	0.8	ACAGGAGAATGCCAAGAATG (CGG)	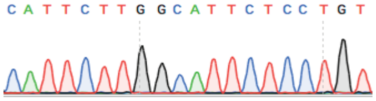
**ZAN, NM_173059**	0.7	CAGGGTGACAGCCAAGAATG (AGG)	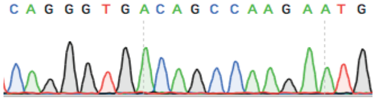
**ZNF540, NM_001172226**	0.7	CCCTATGAATGTCAAGAATG (TGG)	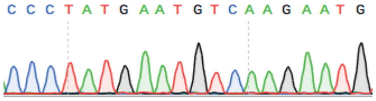
**ZNF331, NM_001253800**	0.7	CCCTATGAATGTCAAGAATG (TGG)	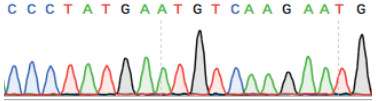
**CLN3, NM_001042432**	0.7	CAGCAGGTGAAGGCCAAGAG (GAG)	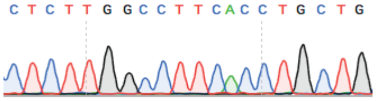
**SNX20, NM_153337**	0.5	TACGGGTTGAAAGCCAAGAG (TGG)	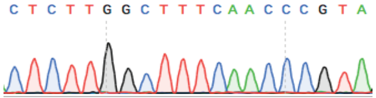
**CCDC62, NM_201435**	0.3	CTTGCGATGAATGCAAAGAG (AAG)	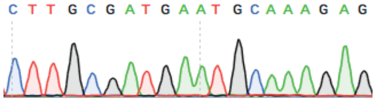

Figure S1. Karyotyping and off-target analysis of gene-edited human iPSCs. Related to Figure 1.

(A) The karyotypes were examined using G-banding of 20 metaphase chromosomal spreads.

(B) Top predicted off-targets and corresponding Sanger sequencing results indicate the lack of off-target gene editing. All on-target scores were > 80.

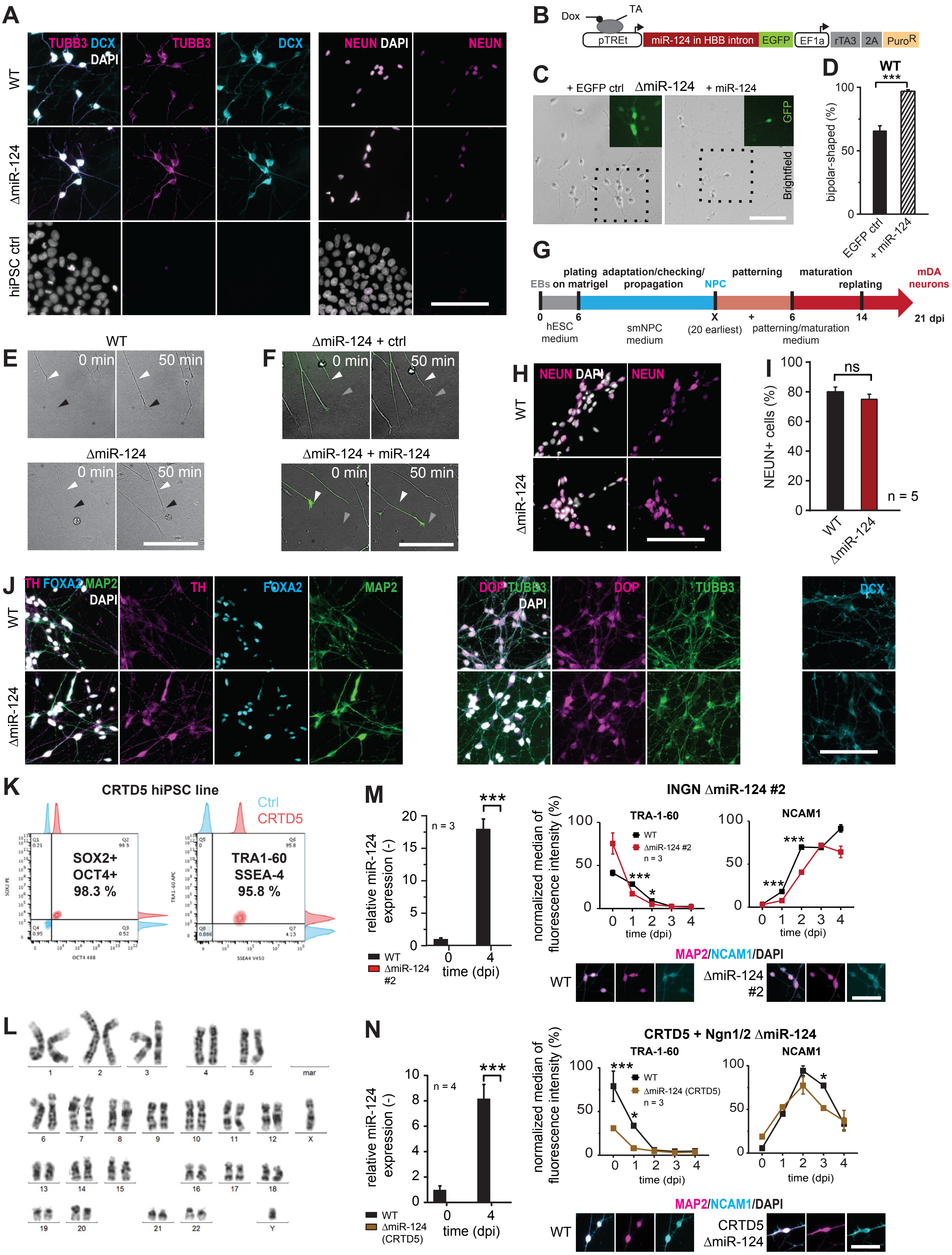

A
TUBB3 DCX DAPI
TUBB3
DCX
NEUN DAPI
NEUN
WT
ΔmiR-124
hiPSC ctrl
B
Dox
TA
pTREt
miR-124 in HBB intron
EGFP
EF1a
rTA3
2A
PuroR
C
+ EGFP ctrl
ΔmiR-124
+ miR-124
GFP
Brightfield
D
WT
***
bipolar-shaped (%)
EGFP ctrl
+ miR-124
G
EBs
plating on matrigel
adaptation/checking/ propagation
NPC
patterning
maturation
replating
mDA neurons
hESC medium
smNPC medium
(20 earliest)
patterning/maturation medium
21 dpi
E
WT
0 min
50 min
ΔmiR-124
F
ΔmiR-124 + ctrl
ΔmiR-124 + miR-124
H
NEUN DAPI
NEUN
I
ns
NEUN+ cells (%)
n = 5
J
TH FOXA2 MAP2 DAPI
TH
FOXA2
MAP2
DOP TUBB3 DAPI
DOP
TUBB3
DCX
K
CRTD5 hiPSC line
Ctrl
CRTD5
SOX2+ OCT4+ 98.3 %
TRA1-60 SSEA-4 95.8 %
M
n = 3
relative miR-124 expression (-)
time (dpi)
WT
ΔmiR-124 #2
INGN ΔmiR-124 #2
TRA-1-60
NCAM1
normalized median of fluorescence intensity (%)
MAP2/NCAM1/DAPI
L
mar
N
n = 4
ΔmiR-124 (CRTD5)
CRTD5 + Ngn1/2 ΔmiR-124
CRTD5 ΔmiR-124

Figure S2. Extended phenotypic analyses of WT and ΔmiR-124 neurons. Related to Figures 1 and 2.

(A) Immunofluorescence staining for the indicated neuronal markers at 4 dpi with hiPSC as control (0 dpi).

(B) Scheme of the miR-124 overexpression PiggyBac vector. miR-124 was expressed from a human β-globin intron that was upstream of an EGFP reporter sequence and driven by a TetOn promoter. Dox – doxycycline, TA – transcriptional activator. The EGFP control vector (EGFP ctrl) lacked the miR-124 sequence.

(C) Representative images of miR-124-overexpressing ΔmiR-124 cells at 4 dpi. Corresponding quantification is shown in Figure 2F. Of note, EGFP ctrl expression was very high.

(D) Bipolar shape quantification of EGFP ctrl and miR-124-overexpressing WT iNGN cells at 4 dpi. The EGFP ctrl expression led to decreased bipolar morphology that was rescued by miR-124 overexpression.

(E) Representative examples for neurite outgrowth capability at 5 dpi, as quantified in Figure 2E.

(F) Representative examples of neurite outgrowth capability of miR-124-overexpressing ΔmiR-124 cells at 5 dpi, as quantified in Figure 2G.

(G) Scheme of the small-molecule-based protocol. EB – embryoid bodies, NPC – neural precursor cells, mDA – midbrain dopaminergic neurons.

(H) Representative images showing NEUN expression in WT and ΔmiR-124 cells, induced by the small-molecule-based protocol shown in (G).

(I) Quantification of NEUN expression of WT and ΔmiR-124 cells, induced by the small-molecule-based protocol as percentage of NEUN-positive nuclei. Statistical analysis, unpaired Student's t-test. Data are represented as mean ± SEM.

(J) Alternative differentiation using small molecules yields dopaminergic neurons for both WT and ΔmiR-124. TH, dopamine, and FOXA2 as dopaminergic markers are expressed, as well as MAP2, TUBB3, and DCX as pan-neuronal markers. Scale bar, 100μm.

(K) Pluripotency flow cytometry analysis of CRTD5 hiPSCs with Alexa Flour 488 anti-Oct3/4, PE anti-SOX2, V450-SSEA-4 and Alexa Flour 647 anti TRA-1-60 antibody markers.

(L) The karyotype of CRTD5 human iPSCs was examined using G-banding of 20 metaphase chromosomal spreads (one example shown).

(M, N) Analysis of a second ΔmiR-124 iNGN clone (M) and a ΔmiR-124 iNGN within the CRTD5 hiPSC background (N). Left, qRT-PCR analysis indicated the lack of miR-124 expression in ΔmiR-124 cells, tested in iPSCs (0 dpi) and neurons (4 dpi). Right, loss of pluripotency (TRA-1-60) and neural lineage commitment (NCAM1) from 0 – 4 dpi measured by flow cytometry using the surface markers indicated. Bottom, representative immunostainings of indicated neuronal markers at 4 dpi reveal miR-124-independent neurogenesis. Scale bar, 50 μm.

Statistical analysis was performed using unpaired Student's t-tests with *$p \leq 0.05$, ***$p \leq 0.001$.

See also Figure S1.

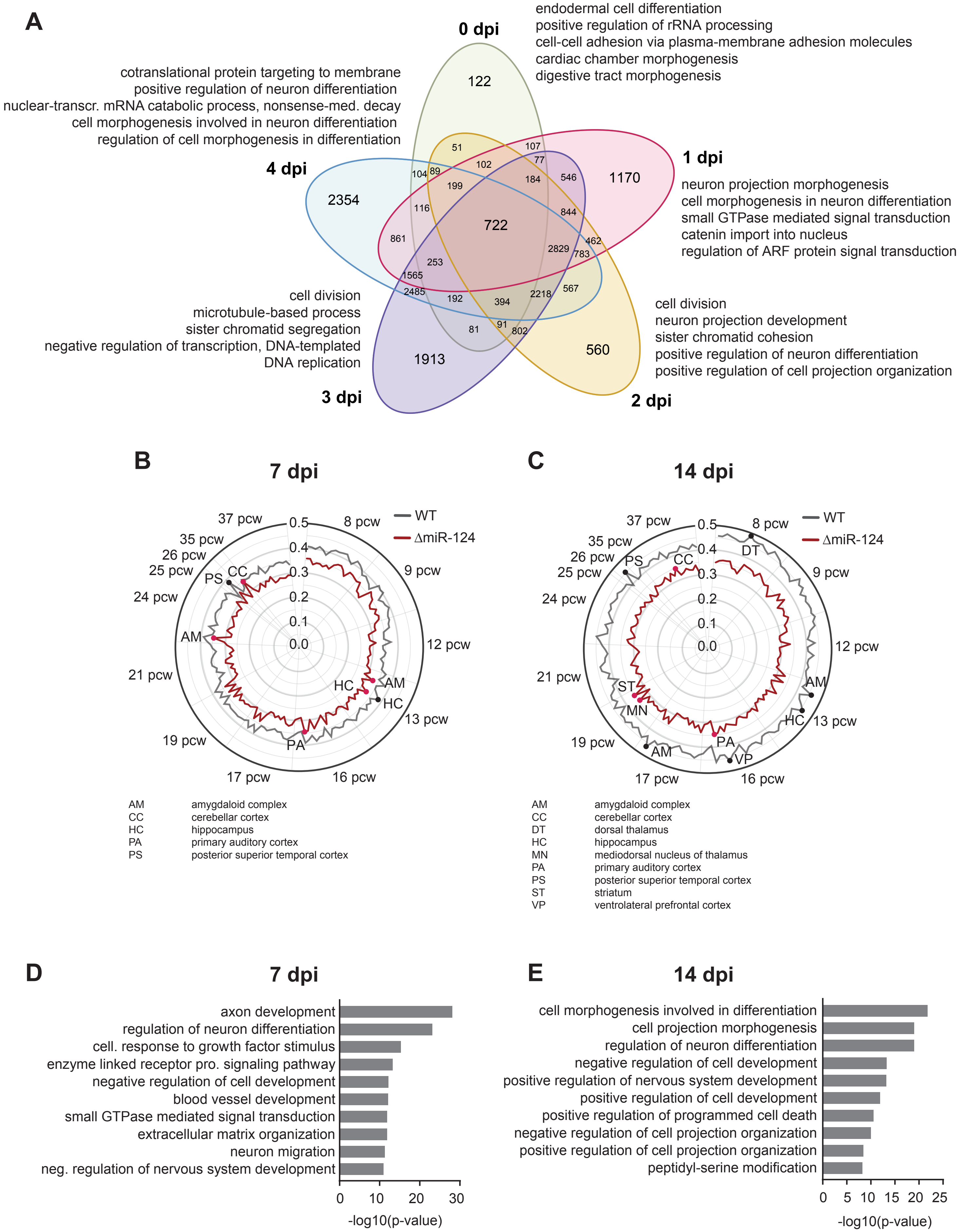
A
0 dpi
1 dpi
2 dpi
3 dpi
4 dpi
endodermal cell differentiation
positive regulation of rRNA processing
cell-cell adhesion via plasma-membrane adhesion molecules
cardiac chamber morphogenesis
digestive tract morphogenesis
cotranslational protein targeting to membrane
positive regulation of neuron differentiation
nuclear-transcr. mRNA catabolic process, nonsense-med. decay
cell morphogenesis involved in neuron differentiation
regulation of cell morphogenesis in differentiation
neuron projection morphogenesis
cell morphogenesis in neuron differentiation
small GTPase mediated signal transduction
catenin import into nucleus
regulation of ARF protein signal transduction
cell division
microtubule-based process
sister chromatid segregation
negative regulation of transcription, DNA-templated
DNA replication
cell division
neuron projection development
sister chromatid cohesion
positive regulation of neuron differentiation
positive regulation of cell projection organization
122
51
107
77
102
104
89
546
1170
184
199
2354
116
844
722
861
462
2829
783
253
1565
567
2485
2218
192
394
91
81
802
560
1913
B
7 dpi
C
14 dpi
WT
ΔmiR-124
8 pcw
9 pcw
12 pcw
13 pcw
16 pcw
17 pcw
19 pcw
21 pcw
24 pcw
25 pcw
26 pcw
35 pcw
37 pcw
0.5
0.4
0.3
0.2
0.1
0.0
PS
CC
AM
HC
PA
DT
ST
MN
VP
AM amygdaloid complex
CC cerebellar cortex
HC hippocampus
PA primary auditory cortex
PS posterior superior temporal cortex
AM amygdaloid complex
CC cerebellar cortex
DT dorsal thalamus
HC hippocampus
MN mediodorsal nucleus of thalamus
PA primary auditory cortex
PS posterior superior temporal cortex
ST striatum
VP ventrolateral prefrontal cortex
D
7 dpi
axon development
regulation of neuron differentiation
cell. response to growth factor stimulus
enzyme linked receptor pro. signaling pathway
negative regulation of cell development
blood vessel development
small GTPase mediated signal transduction
extracellular matrix organization
neuron migration
neg. regulation of nervous system development
0 10 20 30
-log10(p-value)
E
14 dpi
cell morphogenesis involved in differentiation
cell projection morphogenesis
regulation of neuron differentiation
negative regulation of cell development
positive regulation of nervous system development
positive regulation of cell development
positive regulation of programmed cell death
negative regulation of cell projection organization
positive regulation of cell projection organization
peptidyl-serine modification
0 5 10 15 20 25
-log10(p-value)

Figure S3. Extended transcriptome analysis. Related to Figure 3.

(A) Venn diagram for differential gene expression over the time course of differentiation (0 – 4 dpi) comparing WT and ΔmiR-124 with associated GO terms.

(B, C) Correlation analysis of WT and ΔmiR-124 neurons at 7 dpi (B) and 14 dpi (C) to the developing human brain. Pearson correlation of 1000 most differentially expressed genes compared to the transcriptomic Allen Brain Span Atlas dataset for the developmental stages from 8 to 37 postconceptional weeks (pcw).

(D, E) GO term enrichment analysis of differentially expressed genes between WT and ΔmiR-124 samples at 7 dpi (D) and 14 dpi (E) indicating their involvement in neuronal development, maturation and apoptosis.

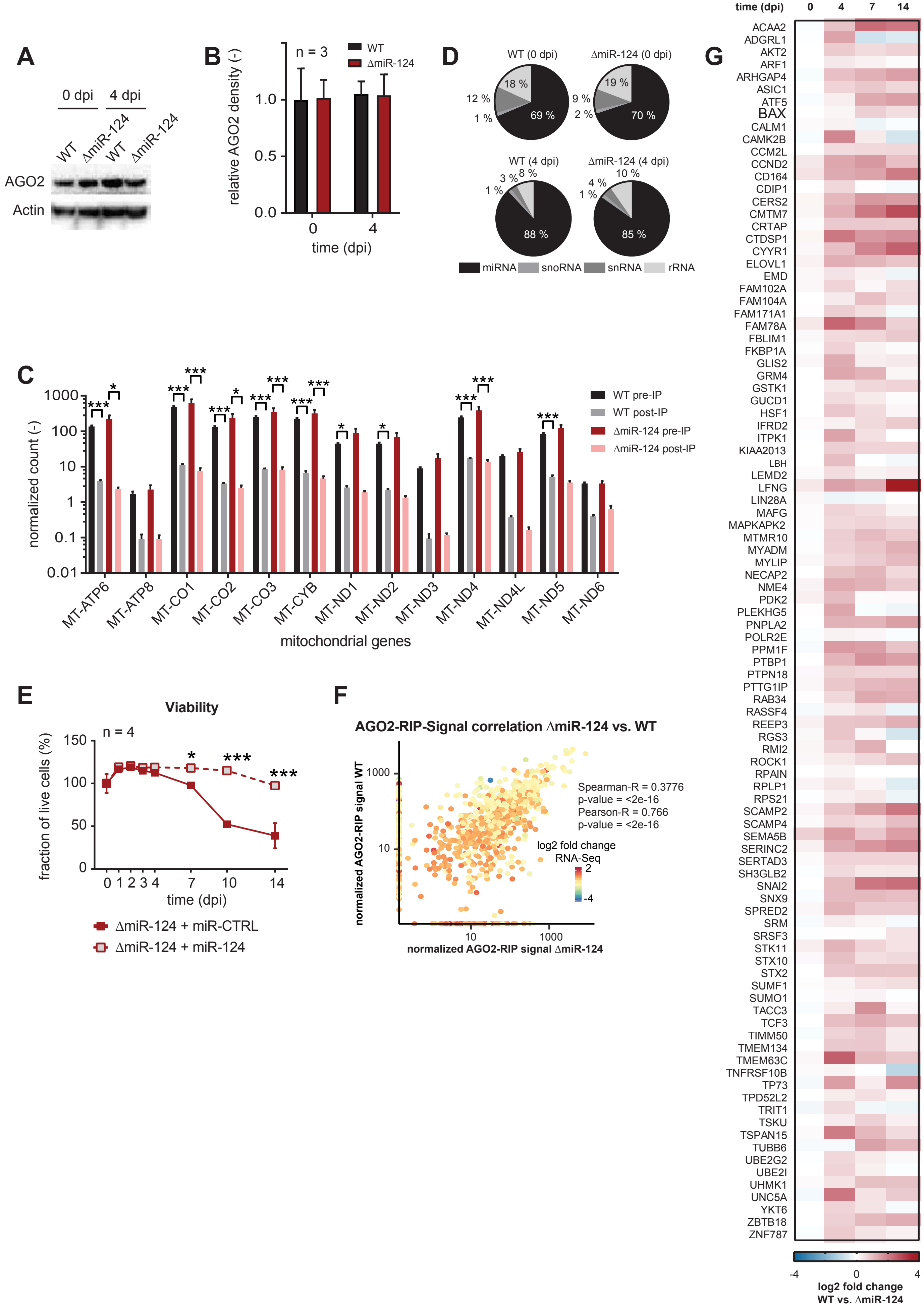

A
0 dpi
4 dpi
WT
ΔmiR-124
WT
ΔmiR-124
AGO2
Actin
B
n = 3
WT
ΔmiR-124
relative AGO2 density (-)
1.0
0.5
0.0
0
4
time (dpi)
D
WT (0 dpi)
18 %
12 %
1 %
69 %
ΔmiR-124 (0 dpi)
19 %
9 %
2 %
70 %
WT (4 dpi)
3 %
8 %
1 %
88 %
ΔmiR-124 (4 dpi)
4 %
10 %
1 %
85 %
miRNA
snoRNA
snRNA
rRNA
C
normalized count (-)
1000
100
10
1
0.1
0.01
WT pre-IP
WT post-IP
ΔmiR-124 pre-IP
ΔmiR-124 post-IP
MT-ATP6
MT-ATP8
MT-CO1
MT-CO2
MT-CO3
MT-CYB
MT-ND1
MT-ND2
MT-ND3
MT-ND4
MT-ND4L
MT-ND5
MT-ND6
mitochondrial genes
E
Viability
n = 4
fraction of live cells (%)
150
100
50
0
0 1 2 3 4
7
10
14
time (dpi)
ΔmiR-124 + miR-CTRL
ΔmiR-124 + miR-124
F
AGO2-RIP-Signal correlation ΔmiR-124 vs. WT
normalized AGO2-RIP signal WT
1000
10
Spearman-R = 0.3776
p-value = <2e-16
Pearson-R = 0.766
p-value = <2e-16
log2 fold change
RNA-Seq
2
-4
10
1000
normalized AGO2-RIP signal ΔmiR-124
G
time (dpi)
0
4
7
14
ACAA2
ADGRL1
AKT2
ARF1
ARHGAP4
ASIC1
ATF5
BAX
CALM1
CAMK2B
CCM2L
CCND2
CD164
CDIP1
CERS2
CMTM7
CRTAP
CTDSP1
CYYR1
ELOVL1
EMD
FAM102A
FAM104A
FAM171A1
FAM78A
FBLIM1
FKBP1A
GLIS2
GRM4
GSTK1
GUCD1
HSF1
IFRD2
ITPK1
KIAA2013
LBH
LEMD2
LFNG
LIN28A
MAFG
MAPKAPK2
MTMR10
MYADM
MYLIP
NECAP2
NME4
PDK2
PLEKHG5
PNPLA2
POLR2E
PPM1F
PTBP1
PTPN18
PTTG1IP
RAB34
RASSF4
REEP3
RGS3
RMI2
ROCK1
RPAIN
RPLP1
RPS21
SCAMP2
SCAMP4
SEMA5B
SERINC2
SERTAD3
SH3GLB2
SNAI2
SNX9
SPRED2
SRM
SRSF3
STK11
STX10
STX2
SUMF1
SUMO1
TACC3
TCF3
TIMM50
TMEM134
TMEM63C
TNFRSF10B
TP73
TPD52L2
TRIT1
TSKU
TSPAN15
TUBB6
UBE2G2
UBE2I
UHMK1
UNC5A
YKT6
ZBTB18
ZNF787
-4
0
4
log2 fold change
WT vs. ΔmiR-124

Figure S4. Target validation and AGO2 precipitation quality control. Related to Figure 4.

(A) Western blot for AGO2 from WT and ΔmiR-124 samples at 0 and 4 dpi.

(B) Quantification of AGO2 western blots (C) using densiometry. n = 3 biological samples.

(C) Normalized count of mitochondrial genes pre- and post-AGO2-IP as indication for IP quality.

(D) Quantification of miRNA, snoRNA, and snRNA species from whole-cell samples. The fraction of miRNAs is significantly increasing from 0 dpi to 4 dpi. Comparisons between WT and ΔmiR-124 are non-significant.

(E) Assessment of cell viability from 0 – 14 dpi for ΔmiR-124 and rescue with miR-124 substitution. The percentage of live cells was measured using the ApoToxGlo assay. The ratio between fluorescence signal for live cells (AFC) and total cell fluorescence (AFC + R110) was examined and normalized to 100 % for 0 dpi.

(F) Correlation of AGO2-RIP signal for ΔmiR-124 and WT indicating differential expression in whole-cell samples as color code (log2 fold change).

(G) Heatmap showing the log2 fold changes of filtered miR-124 targets at 0, 4, 7 and 14 dpi (See also Figure 4C).

Statistical analysis with unpaired Student's t-tests with Holm-Sidak correction for multiple comparisons or 2-way ANOVA with Tukey's multiple comparison test with $*p \leq 0.05$, $**p \leq 0.01$, $***p \leq 0.001$. Data are represented as mean ± SEM

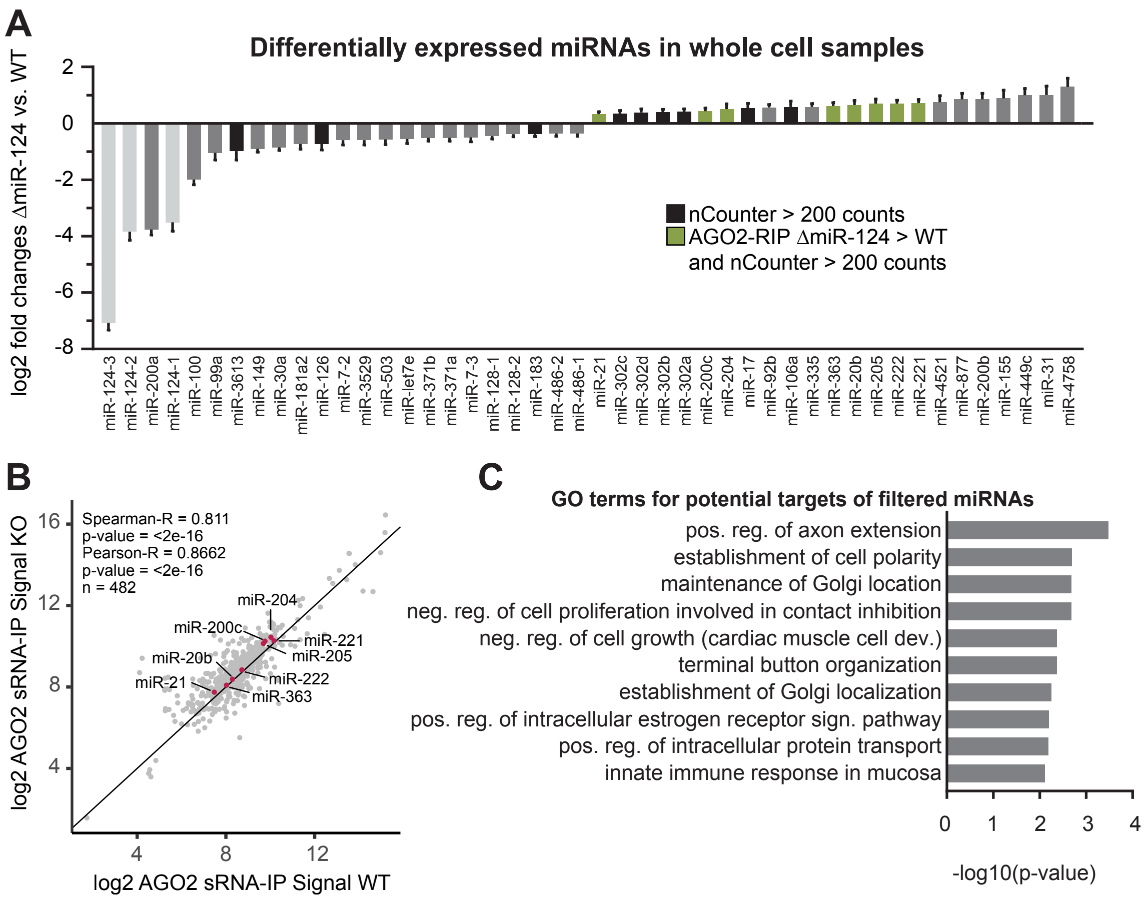
A
Differentially expressed miRNAs in whole cell samples
log2 fold changes ΔmiR-124 vs. WT
2
0
-2
-4
-6
-8
nCounter > 200 counts
AGO2-RIP ΔmiR-124 > WT
and nCounter > 200 counts
miR-124-3
miR-124-2
miR-200a
miR-124-1
miR-100
miR-99a
miR-3613
miR-149
miR-30a
miR-181a2
miR-126
miR-7-2
miR-3529
miR-503
miR-let7e
miR-371b
miR-371a
miR-7-3
miR-128-1
miR-128-2
miR-183
miR-486-2
miR-486-1
miR-21
miR-302c
miR-302d
miR-302b
miR-302a
miR-200c
miR-204
miR-17
miR-92b
miR-106a
miR-335
miR-363
miR-20b
miR-205
miR-222
miR-221
miR-4521
miR-877
miR-200b
miR-155
miR-449c
miR-31
miR-4758
B
Spearman-R = 0.811
p-value = <2e-16
Pearson-R = 0.8662
p-value = <2e-16
n = 482
log2 AGO2 sRNA-IP Signal KO
log2 AGO2 sRNA-IP Signal WT
16
12
8
4
miR-204
miR-200c
miR-221
miR-205
miR-20b
miR-21
miR-222
miR-363
C
GO terms for potential targets of filtered miRNAs
pos. reg. of axon extension
establishment of cell polarity
maintenance of Golgi location
neg. reg. of cell proliferation involved in contact inhibition
neg. reg. of cell growth (cardiac muscle cell dev.)
terminal button organization
establishment of Golgi localization
pos. reg. of intracellular estrogen receptor sign. pathway
pos. reg. of intracellular protein transport
innate immune response in mucosa
0
1
2
3
4
-log10(p-value)

Figure S5. Differentially expressed miRNAs. Related to Figure 5.

(A) Differentially expressed miRNAs from the whole-cell samples as revealed by small RNA-Sequencing at 4 dpi. Data are presented as mean ± lfcSE. Color code indicates miRNAs with more than 200 nCounter counts.

(B) Correlation of AGO2-RIP for ΔmiR-124 and WT at 4 dpi. The eight miRNAs derived from the analysis shown in Figure 5D are marked in red.

(C) GO term analysis for targets of the eight filtered miRNAs (Figure 5D) indicating an involvement in neuronal biological processes.

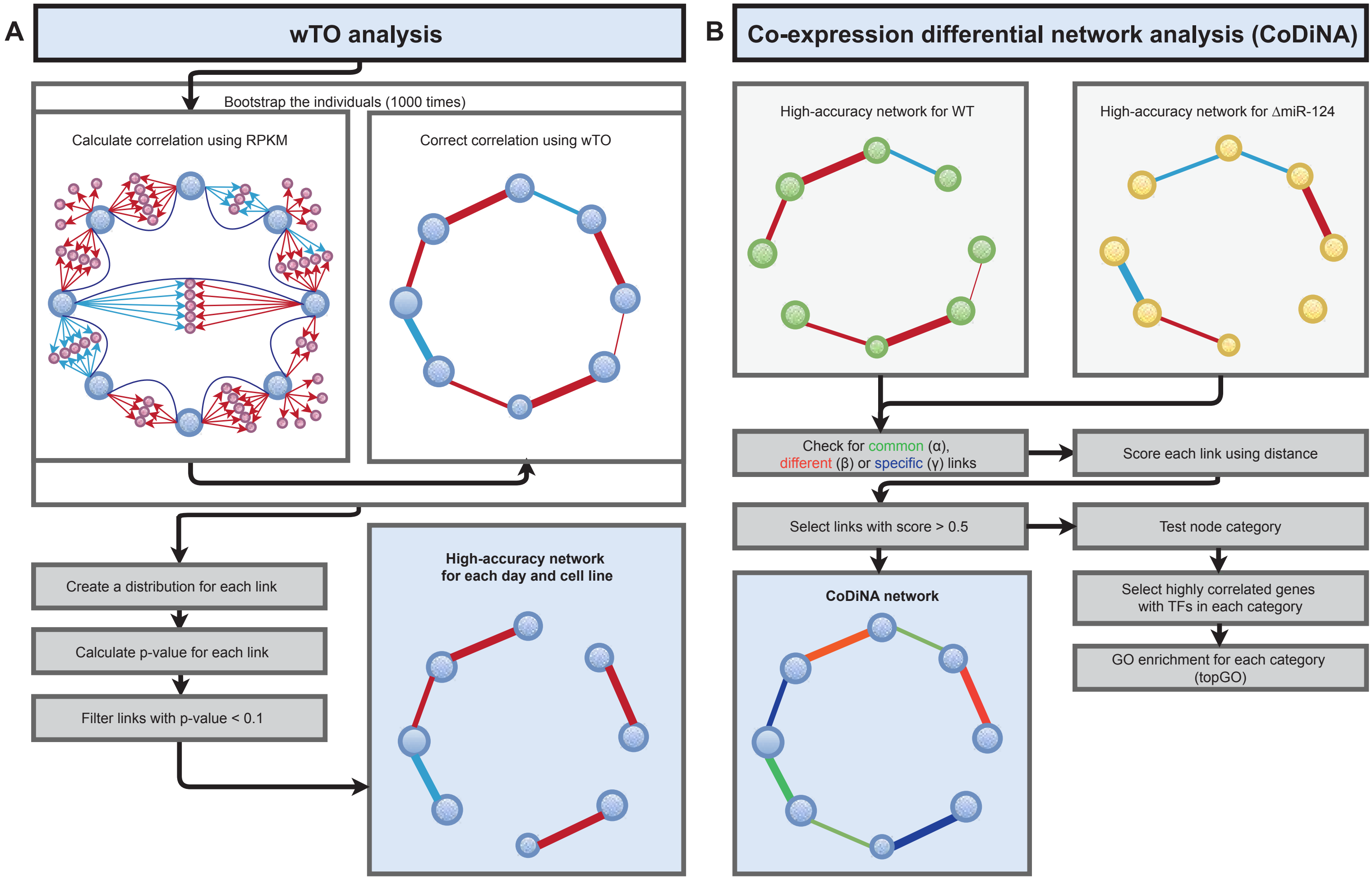
A
wTO analysis
Bootstrap the individuals (1000 times)
Calculate correlation using RPKM
Correct correlation using wTO
Create a distribution for each link
Calculate p-value for each link
Filter links with p-value < 0.1
High-accuracy network
for each day and cell line
B
Co-expression differential network analysis (CoDiNA)
High-accuracy network for WT
High-accuracy network for ΔmiR-124
Check for common (α),
different (β) or specific (γ) links
Score each link using distance
Select links with score > 0.5
Test node category
CoDiNA network
Select highly correlated genes
with TFs in each category
GO enrichment for each category
(topGO)

Figure S6. Illustration of computational network analyses. Related to Figures 6 and 7.

The workflows for the wTO (A) and co-expression differential network (CoDiNA) (B) analyses are shown.

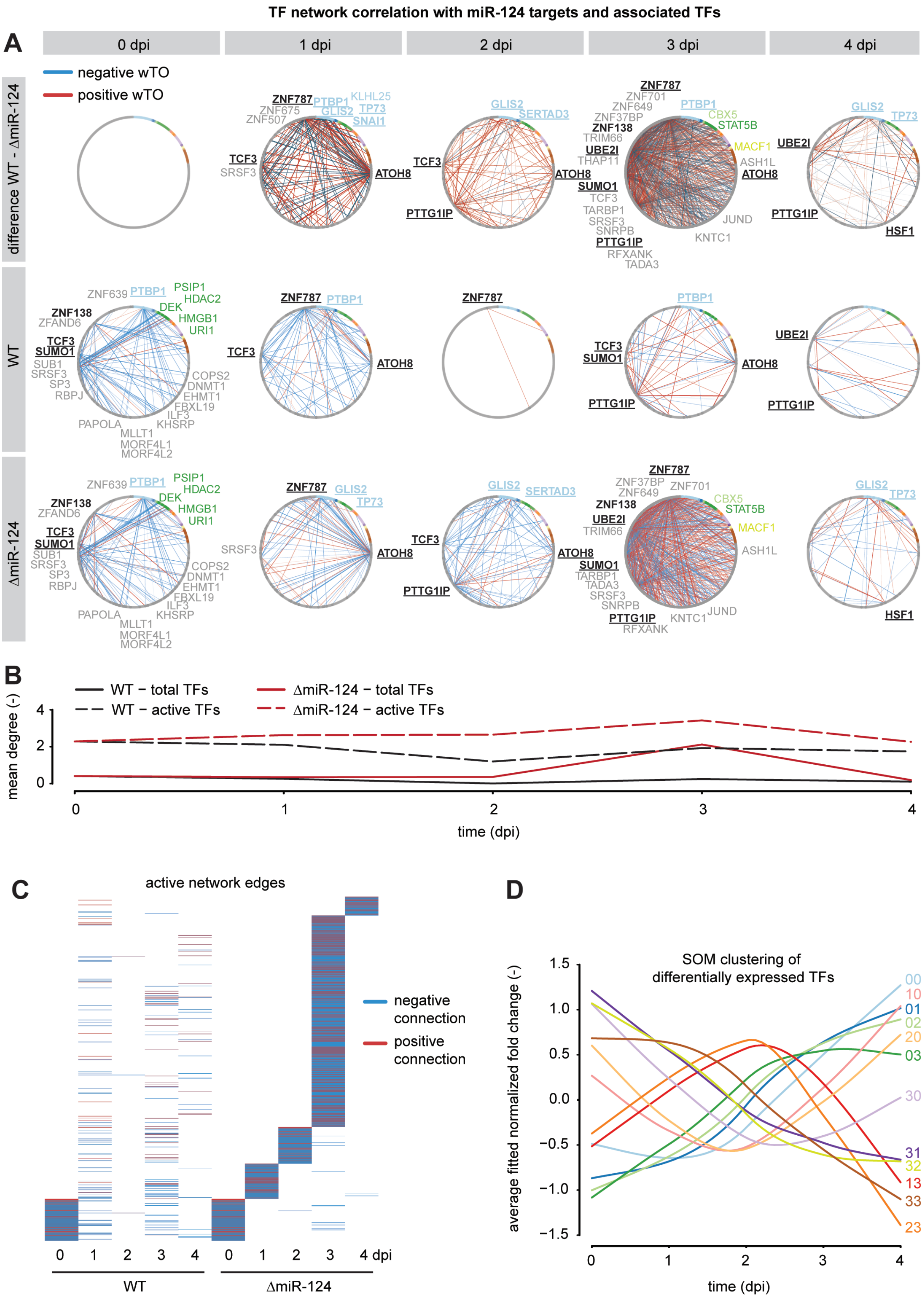

TF network correlation with miR-124 targets and associated TFs
A
0 dpi
1 dpi
2 dpi
3 dpi
4 dpi
negative wTO
positive wTO
difference WT - ΔmiR-124
WT
ΔmiR-124
B
WT − total TFs
WT − active TFs
ΔmiR-124 − total TFs
ΔmiR-124 − active TFs
mean degree (-)
time (dpi)
C
active network edges
negative connection
positive connection
WT
ΔmiR-124
D
SOM clustering of differentially expressed TFs
average fitted normalized fold change (-)
time (dpi)

Figure S7. Timecourse wTO analysis, edge activation and SOM classification of target-TF-network. Related to Figure 6.

(A) Expression correlation (as weighted topological overlap, wTO) between TFs that were differentially expressed on at least one day between 1 dpi and 4 dpi, but not on 0 dpi. Differences in interaction ($|wTO^{WT} - wTO^{\Delta miR\text{-}124}| > 0.2$) are shown in the top panel. Every panel shows the development of the network during differentiation for the difference (top), WT (middle), and ΔmiR-124 (bottom). The opacity of the line indicates the wTO value. Colored gene names represent a specific SOM cluster as shown in Figure S7D. Underlined TFs are miR-124 targets (Figure 4C).

(B) Degree of nodes for each network for active genes and full set of genes over time, as a measure for network strength.

(C) Edge activation of the network depicted gene relationships reappearing on different days.

(D) Loess regression from Self Organizing Maps calculated on the basis of normalized fold changes of permanently (1 dpi – 4 dpi) differentially expressed TFs. Color code represents the SOM categories.
